# Supplementary material for: Multinucleation resets human macrophages for specialized functions at the expense of their identity
Source: EMBO Rep. 2023 Jan 4;24(3):e56310. doi: 10.15252/embr.202256310 (PMC9986822; doi:10.15252/embr.202256310)
Supplement: Supplementary file 1 — Expanded View Figures PDF [file EMBR-24-e56310-s016.pdf]

Expanded View Figures

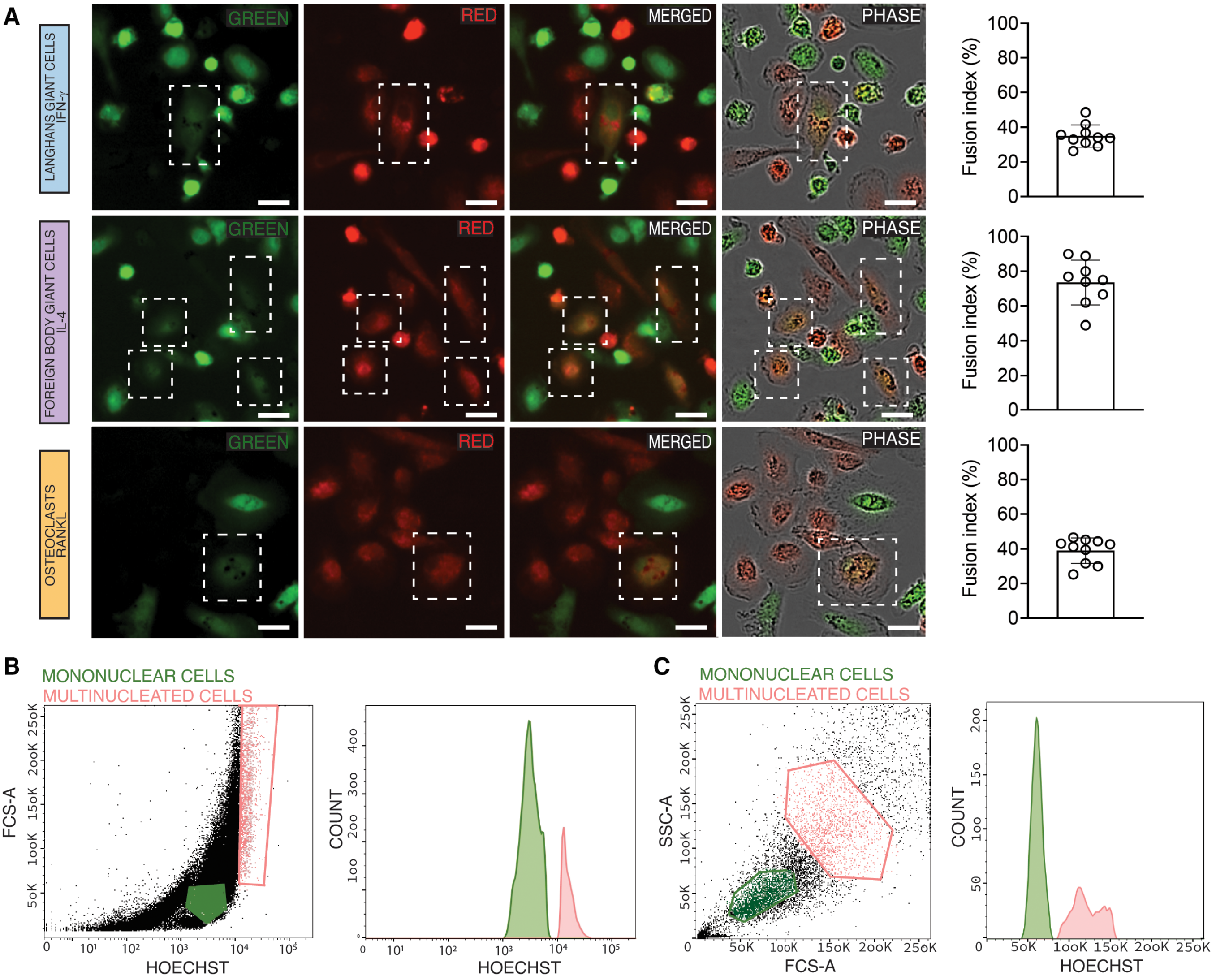

Figure EV1. Multinucleated giant cells are generated through cell–cell fusion.

A Isolated monocytes were labeled with either green or red dyes and stimulated with IFN-γ (LGCs), IL-4 (FBGC), or RANKL (osteoclasts) for giant cell formation. Cell–cell fusion was monitored with live cell imaging system (Incucyte). Orange dye-labeled giant cells (fusion between red and green) are shown within dotted white boxes. The fusion index is calculated for each cell type (right panel). The data are representative of three biological replicates (donors) and 3–4 technical replicates per donor.

B FACS sorting strategy for mononuclear and multinucleated (> 2 nuclei) LGCs and FBGCs based on size and Hoechst (DNA content as a readout of multinucleation).

C FACS sorting strategy for mononuclear and multinucleated (> 2 nuclei) osteoclasts based on size and Hoechst (DNA content as a readout of multinucleation).

Data information: Scale bar, 200 μm (A).

© 2023 The Authors

Figure EV2. Fusion and multinucleation maintain lineage-specific pathways and cause down-regulation of a shared macrophage gene signature.

A Venn diagram showing the trancriptomic comparison between genes upregulated in mononucleated IFN-γ vs. IL-4 stimulated cells (designated as UP [IFN-γ_{mono} vs. IL-4_{mono}]) and in multinucleated LGCs vs. FBGCs (designated as *UP* [IFN-γ_{multi} vs. IL-4_{multi}]). The most significant group-specific pathways (BioPlanet 2019) are shown with arrows. The right panel shows the 5 most significant pathways for commonly upregulated 1,286 transcripts. Relevant pathways to IFN- γ are shown in red.

B Venn diagram showing the trancriptomic comparison between genes upregulated in mononucleated IL-4 vs. IFN-γ stimulated cells (designated as *UP* [IL-4_{mono} vs. IFN-γ_{mono}]) and in multinucleated FBGCs vs. LGCs (designated as *UP* [IL-4_{multi} vs. IFN-γ_{multi}]). The most significant group-specific pathways are shown with arrows whereas. The right panel shows the 5 most significant pathways for commonly upregulated 1,027 transcripts. Relevant pathways to IL-4 are shown in red. Data information: $n = 6$ donors (LGCs), $n = 7$ donors (FBGCs).

C *MRC1*, *CSF1R*, *TLR2*, *STAB1 and SUCNR1* relative expression measured by qRT–PCR in LGCs (upper), FBGCs (middle) and osteoclasts (lower), in sorted mononuclear and multinucleated cells; at least $n = 6$ donors. Error bars are mean ± SD; significance tested by paired t-test; *$P < 0.05$; **$P < 0.01$; ***$P < 0.001$; ****$P < 0.0001$.

© 2023 The Authors

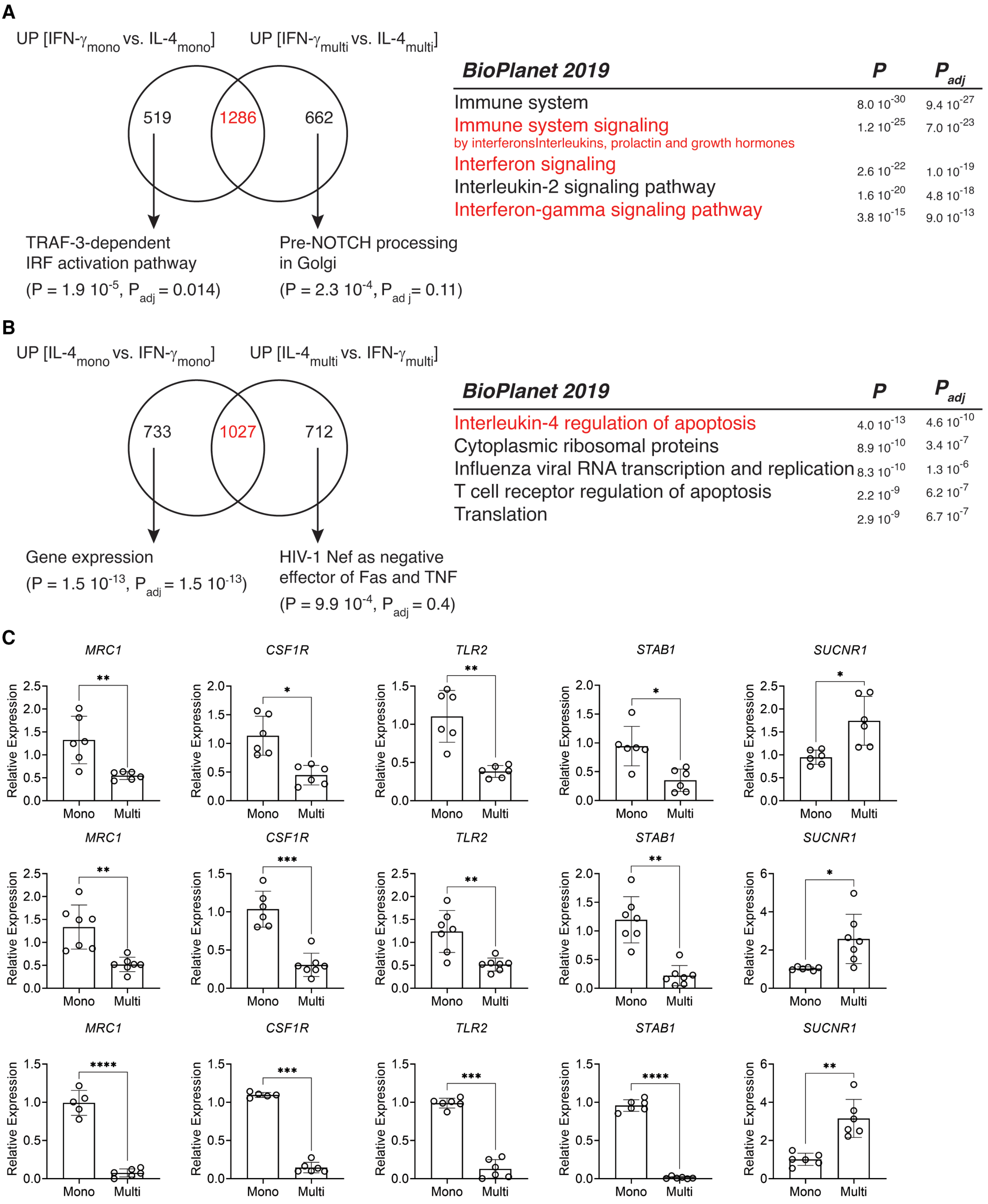

Figure EV2.

© 2023 The Authors

Figure EV3. Fusion and multinucleation causes the downregulation of a shared macrophage gene signature between LGCs, FBGCs and osteoclasts.

A Protein–protein interaction (PPI) network of 191 commonly downregulated genes in LGCs, FBGCs and osteoclasts illustrated by STRING (high confidence score = 0.9, only connected nodes are shown).

B ImageStream gating for mononuclear and multinucleated cells shown in LGCs. The gating strategy for FBGCs and osteoclasts is similar.

C ImageStream showing bright field, nuclei staining (Hoechst), and MRC1 (red) staining in mononuclear and multinucleated LGCs, FBGCs and osteoclasts.

D MRC1 and CSF1R immunofluorescence quantification in mononuclear and multinucleated LGCs (upper), FBGCs (middle) and osteoclasts (lower); $n = 2$ donors (biological replicates), $n > 48$ technical replicates per cell type, condition (mono or multi) and surface marker (MRC1 or CSF1R).

E DC-STAMP expression following its knockdown in LGCs (left) and FBGCs (right). si-Ctrl, scrambled siRNA; si-DC-STAMP, DC-STAMP siRNA; $n = 7$ donors.

F Fusion index following DC-STAMP knockdown in human LGCs (upper panel) and FBGCs (lower panel); $n = 4$ donors. Fusion was measured in cells stained with Giemsa in both conditions (right panel).

Data information: Error bars are mean ± SD; significance tested by unpaired (D) and paired (E, F) *t*-test; $^{**}P < 0.01$; $^{****}P < 0.0001$; scale bar, 20 μm (C), 100 μm (F).

© 2023 The Authors

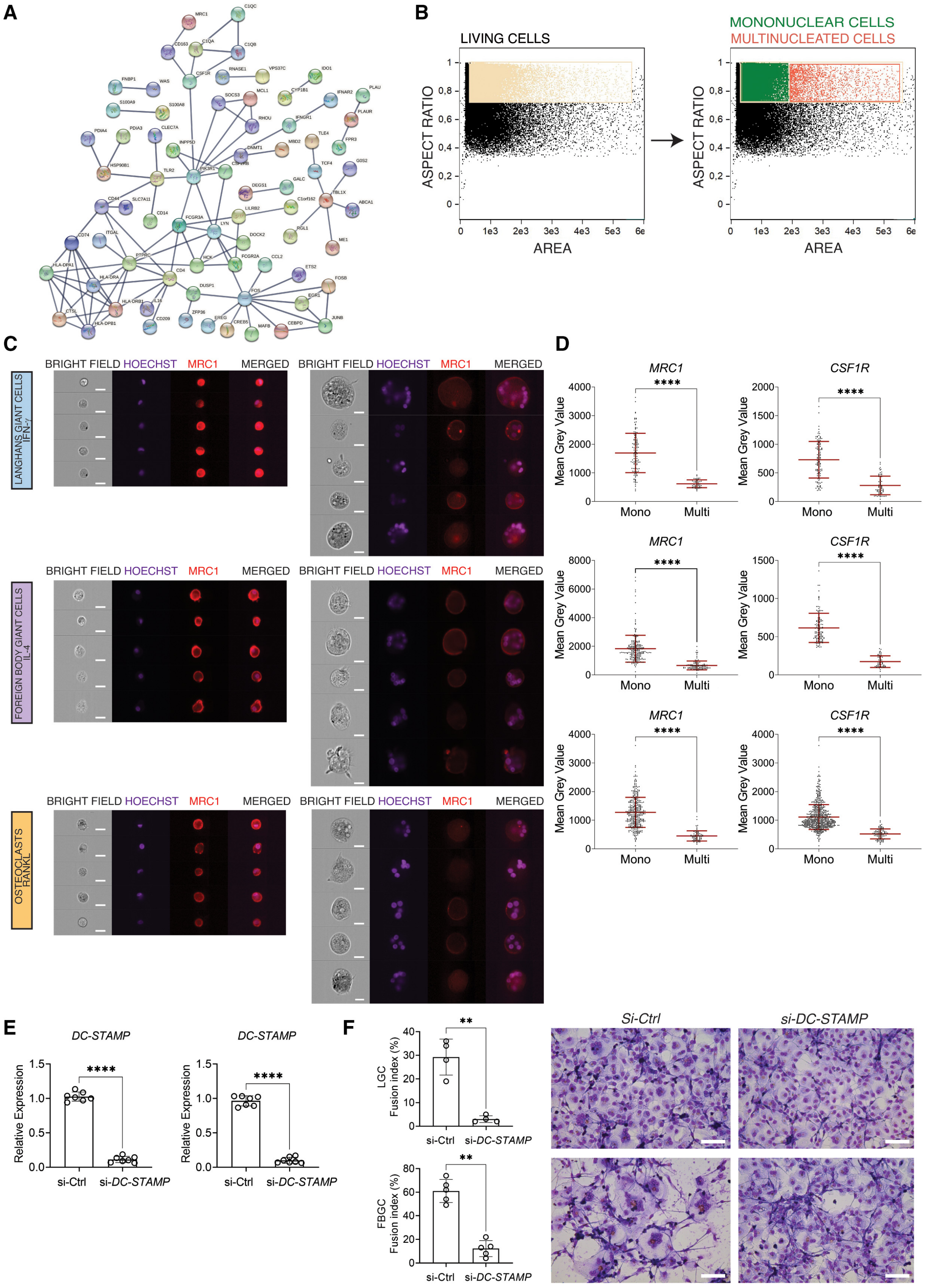

Figure EV3.

 © 2023 The Authors

Figure EV4. Multinucleation confers cluster-forming properties to LGCs *in vitro*.

A CD3 immunofluorescence in PBMC-derived FBGCs (upper panel) and osteoclasts (lower panel). Hoechst (gray) and phalloidin (red) staining show the nuclei and cytoskeleton, respectively.

B LGCs show increased membrane expression of B7-H3. ImageStream showing bright field, nuclei staining (Hoechst), and B7-H3 (red) staining in mononuclear and multinucleated LGCs, FBGCs and osteoclasts.

Data information: Scale bar, 20 μm.

© 2023 The Authors

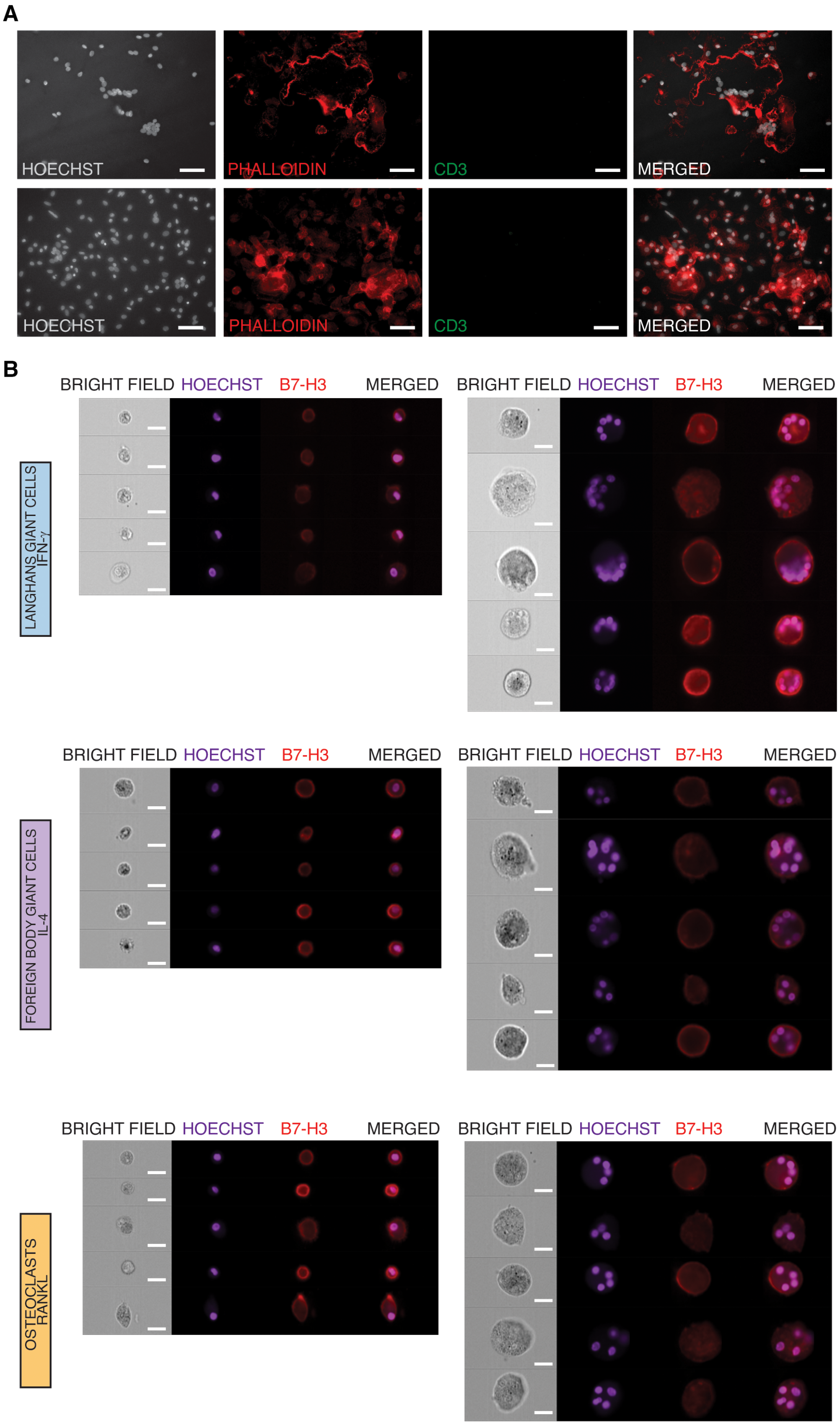

Figure EV4.

 © 2023 The Authors

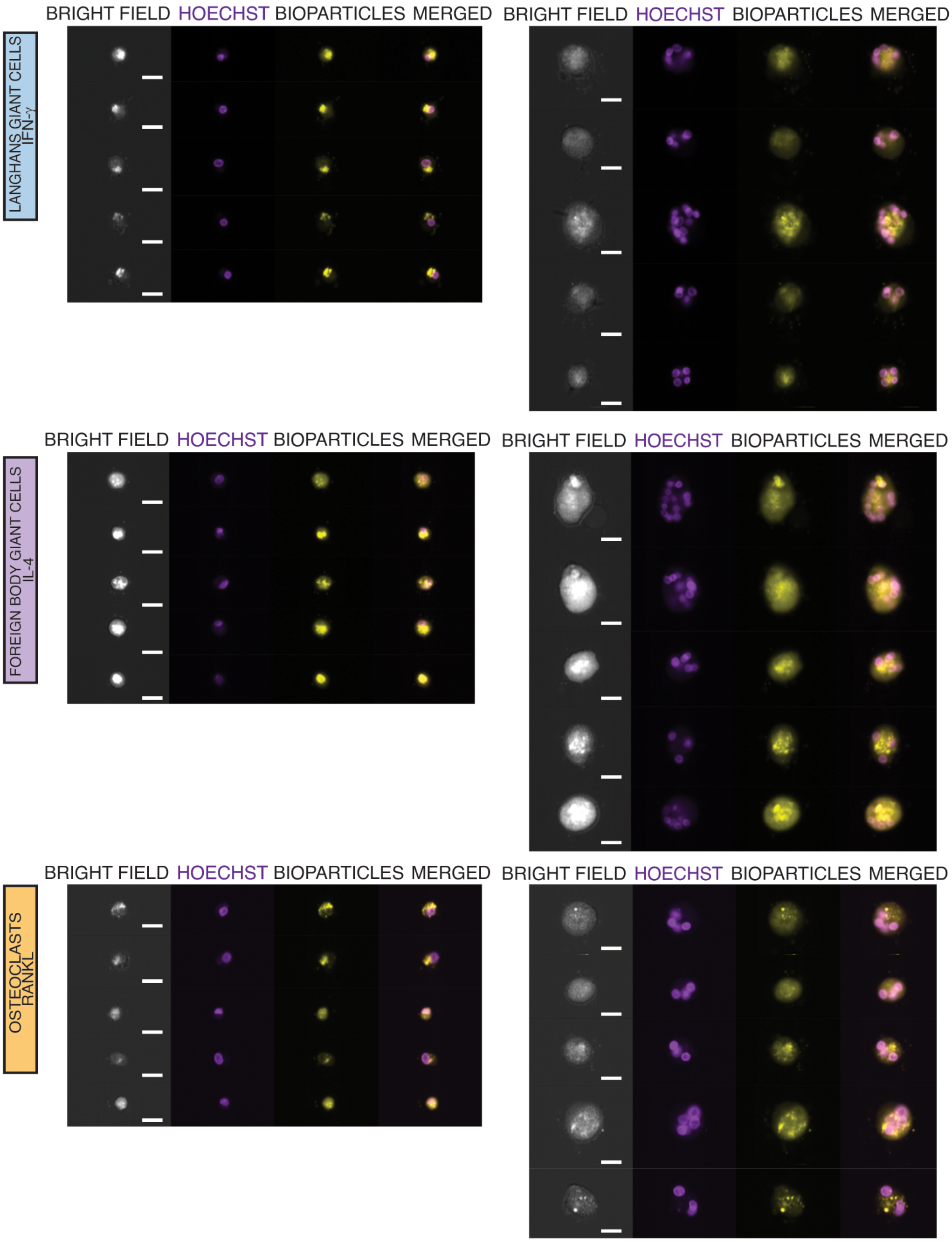

Figure EV5. Multinucleation causes enhanced phagocytosis in FBGCs.

FBGCs show a distinctively enhanced phagocytic capacity. ImageStream showing bright field, nuclei staining (Hoechst), and *S. aureus*-coated bioparticles (yellow) staining in mononuclear and multinucleated LGCs, FBGCs and osteoclasts. Scale bar, 20 µm.

© 2023 The Authors
