# Supplementary material for: Multinucleation resets human macrophages for specialized functions at the expense of their identity
Source: EMBO Rep. 2023 Jan 4;24(3):e56310. doi: 10.15252/embr.202256310 (PMC9986822; doi:10.15252/embr.202256310)
Supplement: Supplementary file 9 — PDF+ [file EMBR-24-e56310-s010.pdf]

Article

SOURCE DATA | TRANSPARENT PROCESS | OPEN ACCESS

EMBO reports

Multinucleation resets human macrophages for specialized functions at the expense of their identity

Kourosh Ahmadzadeh[1,†], Marie Pereira[2,†], Margot Vanoppen[1], Eline Bernaerts[1], Jeong-Hun Ko[2], Tania Mitera[1], Christy Maksoudian[3], Bella B Manshian[4], Stefaan Soenen[3], Carlos D Rose[5], Patrick Matthys[1,*,†], Carine Wouters[1,6,7,**,†] & Jacques Behmoaras[2,8,***]

Abstract

Macrophages undergo plasma membrane fusion and cell multinucleation to form multinucleated giant cells (MGCs) such as osteoclasts in bone, Langhans giant cells (LGCs) as part of granulomas or foreign-body giant cells (FBGCs) in reaction to exogenous material. How multinucleation *per se* contributes to functional specialization of mature mononuclear macrophages remains poorly understood in humans. Here, we integrate comparative transcriptomics with functional assays in purified mature mononuclear and multinucleated human osteoclasts, LGCs and FBGCs. Strikingly, in all three types of MGCs, multinucleation causes a pronounced downregulation of macrophage identity. We show enhanced lysosome-mediated intracellular iron homeostasis promoting MGC formation. The transition from mononuclear to multinuclear state is accompanied by cell specialization specific to each polykaryon. Enhanced phagocytic and mitochondrial function associate with FBGCs and osteoclasts, respectively. Moreover, human LGCs preferentially express B7-H3 (CD276) and can form granuloma-like clusters *in vitro*, suggesting that their multinucleation potentiates T cell activation. These findings demonstrate how cell–cell fusion and multinucleation reset human macrophage identity as part of an advanced maturation step that confers MGC-specific functionality.

Keywords foreign body giant cells; Langhans giant cells; macrophages; multinucleation; osteoclasts

Subject Categories Development; Immunology; Membrane & Trafficking

DOI 10.15252/embr.202256310 | Received 17 October 2022 | Revised 8 December 2022 | Accepted 9 December 2022 | Published online 4 January 2023

EMBO Reports (2023) 24: e56310

Introduction

Cell–cell fusion in monocyte/macrophage lineage leads to the formation of diverse multinucleated giant cells (MGCs), depending on the tissue microenvironment. MGCs can be classified into osteoclasts, Langhans giant cells (LGCs) and foreign body giant cells (FBGCs), on the basis of their anatomical site, morphology and function during homeostasis or inflammatory disease (Helming & Gordon, 2007; Pereira *et al*, 2018; Ahmadzadeh *et al*, 2022). Osteoclasts are macrophage-derived multinucleated cells specialized in vertebrate bone remodeling and can be considered as homeostatic MGCs that turn-over during adult life (Boyle *et al*, 2003). Conversely, LGCs and FBGCs are preferentially found in pathological sites during inflammatory processes. LGCs are the hallmark of infectious (tuberculosis) and non-infectious (sarcoidosis, Blau syndrome) granulomatous diseases (Ahmadzadeh *et al*, 2022). FBGCs can be found at the site of implanted prostheses or medical devices (Anderson *et al*, 2008) and are specialized in complement-mediated phagocytosis of large particles (Milde *et al*, 2015). To date, the precise role of LGCs remains unclear. Recent studies have provided the immune landscape of infectious granulomas at a single-cell resolution (Ma *et al*, 2021; McCaffrey *et al*, 2022) but whether LGC presence is beneficial or detrimental to the disease outcome is currently open to debate.

1 Laboratory of Immunobiology, Department Microbiology, Immunology and Transplantation, Rega Institute, KU Leuven—University of Leuven, Leuven, Belgium
2 Centre for Inflammatory Disease, Department of Immunology and Inflammation, Hammersmith Hospital, Imperial College London, London, UK
3 NanoHealth and Optical Imaging Group, Translational Cell and Tissue Research Unit, Department of Imaging and Pathology, KU Leuven, Leuven, Belgium
4 Translational Cell and Tissue Research Unit, Department of Imaging and Pathology, KU Leuven, Leuven, Belgium
5 Division of Pediatric Rheumatology Nemours Children's Hospital, Thomas Jefferson University, Philadelphia, PA, USA
6 Division Pediatric Rheumatology, UZ Leuven, Leuven, Belgium
7 European Reference Network for Rare Immunodeficiency, Autoinflammatory and Autoimmune Diseases (RITA) at University Hospital Leuven, Leuven, Belgium
8 Programme in Cardiovascular and Metabolic Disorders and Centre for Computational Biology, Duke-NUS Medical School Singapore, Singapore, Singapore
*Corresponding author. Tel: +32497122503: E-mail: patrick.matthys@kuleuven.be
**Corresponding author. Tel: +32475415459: E-mail: carine.wouters@uzleuven.be
***Corresponding author. Tel: +6566011137: E-mail: jacquesb@duke-nus.edu.sg
[†]These authors contributed equally to this work

© 2023 The Authors. Published under the terms of the CC BY 4.0 license.

Despite their divergent cell function, the common feature between osteoclasts, LGCs and FBGCs is their monocyte/macrophage origin and their multinucleated cell appearance following cell–cell fusion. In order to give rise to mature multinucleated cells, macrophages go through two key phases of cell differentiation. The first is governed by tissue-dependent signals that lead to fusion-competency (e.g. RANKL for bone osteoclasts; Takegahara *et al*, 2022). This step is followed by cell–cell fusion (Brukman *et al*, 2019) and multinucleation of mature macrophages and formation of MGCs with specialized functions (Helming & Gordon, 2009).

Among the three types of MGCs, studies on osteoclasts improved considerably our understanding of how lineage-specific signals followed by cell–cell fusion shape cell activity during homeostasis and disease. Indeed, functional osteoclasts capable of resorbing bone are multinucleated macrophages that differentiate through the concerted action of macrophage-colony-stimulating factor (M-CSF) and RANKL (Novack & Teitelbaum, 2008; Teitelbaum, 2011; Tsukasaki *et al*, 2020; Takegahara *et al*, 2022). Osteoclasts originate from embryonic erythro-myeloid progenitors and their postnatal maintenance is mediated by acquisition of new nuclei from circulating blood cells (Jacome-Galarza *et al*, 2019). Once mature, multinucleated osteoclasts can undergo fission and form transcriptionally distinct cells called osteomorphs (McDonald *et al*, 2021). Mature osteoclasts contain up to approximately 20 nuclei in normal human bones (Vignery, 2000; Bar-Shavit, 2007) and during pathological conditions, active osteoclasts may contain over 50 nuclei (Roodman, 1996), indicative of an association between the number of nuclei and cell activity. From a genetic point of view, conserved transcriptional gene networks in multinucleating mature osteoclasts control their resorption activity and the resulting bone mass (Kang *et al*, 2014; Pereira *et al*, 2018, 2020). In summary, fusion and multinucleation of osteoclasts give rise to a separate, specialized cell stage, which is absent in mononuclear cells. Paradoxically, even though extensive efforts went into understanding the biology of osteoclast differentiation (i.e. osteoclastogenesis), relatively less is known about the transition from a RANKL-induced mononuclear into a fused multinuclear osteoclast state and the resulting post-fusion transcriptional reprogramming in these cells.

The molecular consequences of cell–cell fusion and multinucleation remain poorly defined in mature human osteoclasts, LGCs and FBGCs. One obstacle for side-by-side comparison of fusing MGCs has been the identification of lineage determining factors and their respective efficacy to confer fusion-competency. RANKL is a well-established *in vitro* differentiation signal for osteoclasts but the soluble factors responsible for triggering cell fusion and multinucleation in LGCs and FBGCs are relatively less studied. Although previous evidence points toward IFN-γ and IL-4 in LGCs and FBGCs, respectively (Weinberg *et al*, 1984; McNally & Anderson, 1995), it is not understood to what extend these two stimuli recap the functional characteristics of LGCs and FBGCs in humans. Here we investigated the mechanisms governing macrophage fusion and multinucleation in mature LGCs, FBGCs and osteoclasts differentiated from healthy donors. We first showed that the usage of a single cytokine (RANKL for osteoclasts, IFN-γ for LGCs and IL-4 for FBGCs) in presence of M-CSF, can recap the typical multinucleated morphological appearance of these primary human cells. The isolation of purified mononuclear and multinucleated LGCs, FBGCs and osteoclasts allowed us to perform comparative transcriptomics and focus on shared and cell-type specific observations during macrophage multinucleation. As part of shared pathways, we show that multinucleation causes a drastic downregulation of macrophage identity meanwhile lysosome-dependent iron homeostasis is enhanced. Cell type-specific features include FBGCs showing improved ability of phagocytosis and osteoclasts maximizing their mitochondrial activity following multinucleation. Furthermore, human LGCs can form granuloma-like clusters *in vitro*. Our results show that macrophage fusion and multinucleation reprograms the cell for specialized functions and causes loss of a core myeloid gene signature.

Results

Fusion and multinucleation reshapes the myeloid transcriptome in differentiated human MGCs

We generated three types of multinucleated giant cells (MGCs) using macrophages isolated from human donor-derived peripheral blood mononuclear cells (PBMCs) (Fig 1A). In addition to RANKL used for osteoclasts, IFN-γ and IL-4 were added to generate mature LGCs and FBGCs, respectively (Fig 1A). As expected, IFN-γ induced LGCs were characterized by a ring of nuclei along the cell border, while IL-4-induced FBGCs showed scattered nuclei throughout the cytoplasm (Fig 1A and B) (Adams, 1976; McNally & Anderson, 1995; Pagan & Ramakrishnan, 2018). The distinct morphological appearance of these three types of MGCs (Fig 1B) suggested cell type-specific functional properties and shared mechanisms underlying macrophage multinucleation. Macrophage fusion and multinucleation include a differentiation step from progenitor cells that is achieved with lineage-specific signals (e.g. RANKL, IFN-γ, IL-4). Following the differentiation step, cell fusion and multinucleation lead to mature LGCs, FBGCs and osteoclasts (Pereira *et al*, 2018; Ahmadzadeh *et al*, 2022). Here, we aimed to investigate the functional consequences of human macrophage fusion/multinucleation post-cell differentiation. We first confirmed that MGCs are generated through cell membrane fusion in all 3 cell types (Fig EV1A). Next, we set-up a cell sorting strategy (Fig EV1B and C, and Materials and Methods), resulting in > 85% purity in mononuclear and multinucleated LGCs, FBGCs and osteoclasts after their respective differentiation with lineage-specific soluble factors (Fig 1B). Before performing a comprehensive RNA-seq analysis between mononucleated and multinucleated macrophages, we verified existence of lineage-dependent pathways and markers of LGCs and FBGCs as these cells are relatively less well-defined compared to osteoclasts. As expected, and in line with the lineage-dependency, the transcriptomics comparison between mononucleated/multinucleated IFN-γ and IL-4-differentiated macrophages showed predominance of IFN-γ and IL-4-related pathways, respectively (Fig EV2A and B; Dataset EV1). Among known LGC and FBGC markers, we confirmed upregulation of *CCL7* (Chen *et al*, 2022) and *CD86* (McNally & Anderson, 2011), respectively (Dataset EV1). Interestingly, although less significant, this analysis also showed pathways that were specific to the mononucleated or multinucleated state in IFN-γ-differentiated macrophages when compared to IL-4-differentiated ones and *vice versa* (Fig EV2A and B). Following this verification step, we performed RNA-seq analysis between mononuclear and multinucleated cells. This allowed us to minimize cofounding lineage signal effects

 © 2023 The Authors

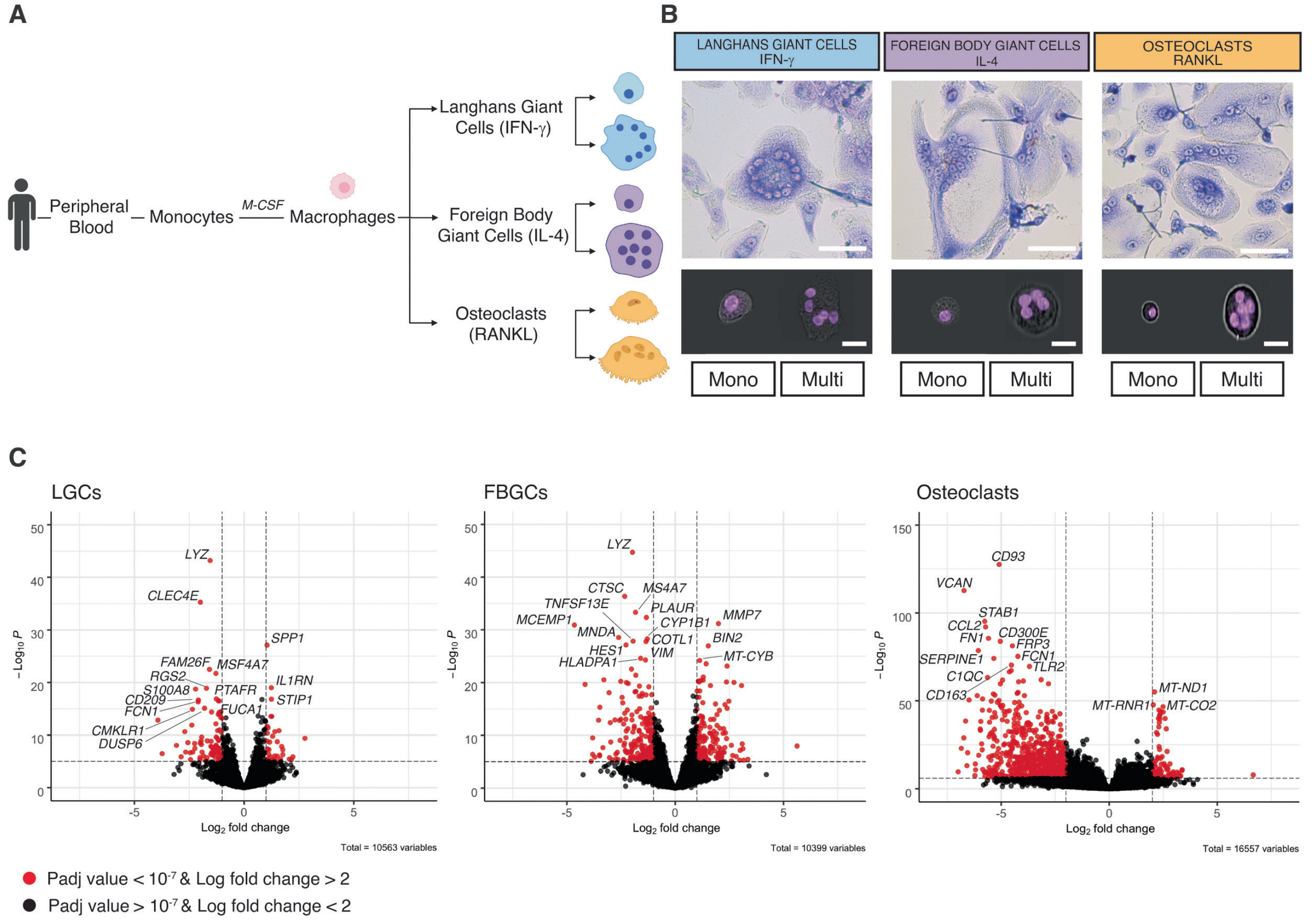

Figure 1. Human macrophage fusion and multinucleation reprograms the transcriptome in differentiated Langhans giant cells (LGCs), foreign body giant cells (FBGCs) and osteoclasts.

A Schematic overview of the PBMC-derived generation of differentiated mononuclear and multinucleated LGCs, FBGCs and osteoclasts using IFN-γ, IL-4 and RANKL, respectively.

B Light microscopy images of LGCs, FBGCs and osteoclasts stained with Giemsa staining (upper panel). Representative Hoechst dye images acquired by ImageStream (lower panel) of sorted cells showing mononuclear (mono) and multinucleated (multi) populations for each cell type.

C RNA-seq volcano plots highlighting the top 15 differentially expressed genes between mononuclear and multinucleated populations for each cell type.

Data information: Scale bar, 100 μm (Panel B, upper panel); 20 μm (Panel B, lower panel); $n = 6$ donors (LGCs), $n = 7$ donors (FBGCs) and $n = 6$ donors (osteoclasts). Source data are available online for this figure.

(IFN-γ, IL-4, RANKL) and focus on transcriptional pathways arising from multinucleation *per se*. The top differentially expressed genes between mononuclear and multinucleated cells included markers of tissue-resident macrophages such as *LYZ* (MacParland *et al*, 2018), *MSA4A7* (Van Hove *et al*, 2019), *S100A8* (Mulder *et al*, 2021) and of pattern recognition receptors (*TLR2*, *CLEC4E*, *PTAFR*, *FCN1*; Fig 1C), suggesting a transcriptional reprogramming of myeloid identity, which we set out to investigate in more detail.

Multinucleation suppresses a shared mononuclear phagocyte gene signature in humans

We first analyzed the commonly downregulated genes as a result of multinucleation in LGCs, FBGCs and osteoclasts (Fig 2A). These 191 transcripts (Dataset EV2) belong to pathways that include tuberculosis, phagosome, osteoclasts and more generally the immune system (Fig 2B). Remarkably, these commonly downregulated genes contain a set of transcripts that defines mononuclear phagocytes and we confirmed their multinucleation-induced suppression by qRT–PCR (Fig EV2C). Among these 191 transcripts, pathogen recognition (*TLR2*, *CD14*, *CLEC7A*), antigen presentation (*HLA-DPA1*, *HLA-DRA*, *HLA-DRB1*, *HLA-DPB1*, *CD74*), tissue resident macrophage (*CSF1R*, *MRC1*, *CD163*, *MAFB*, *C1QA/B/C*) transcripts form a protein–protein interaction network (PPI, Fig EV3A). Osteoclast lineage determinant transcription factor *FOS* (Grigoriadis *et al*, 1994), macrophage-specific transcription factor *MAFB* (Aziz *et al*, 2009; Lavin *et al*, 2014) and LGC multinucleation inducer *TLR2* (Hertwich *et al*, 2016) are among the transcripts forming the core

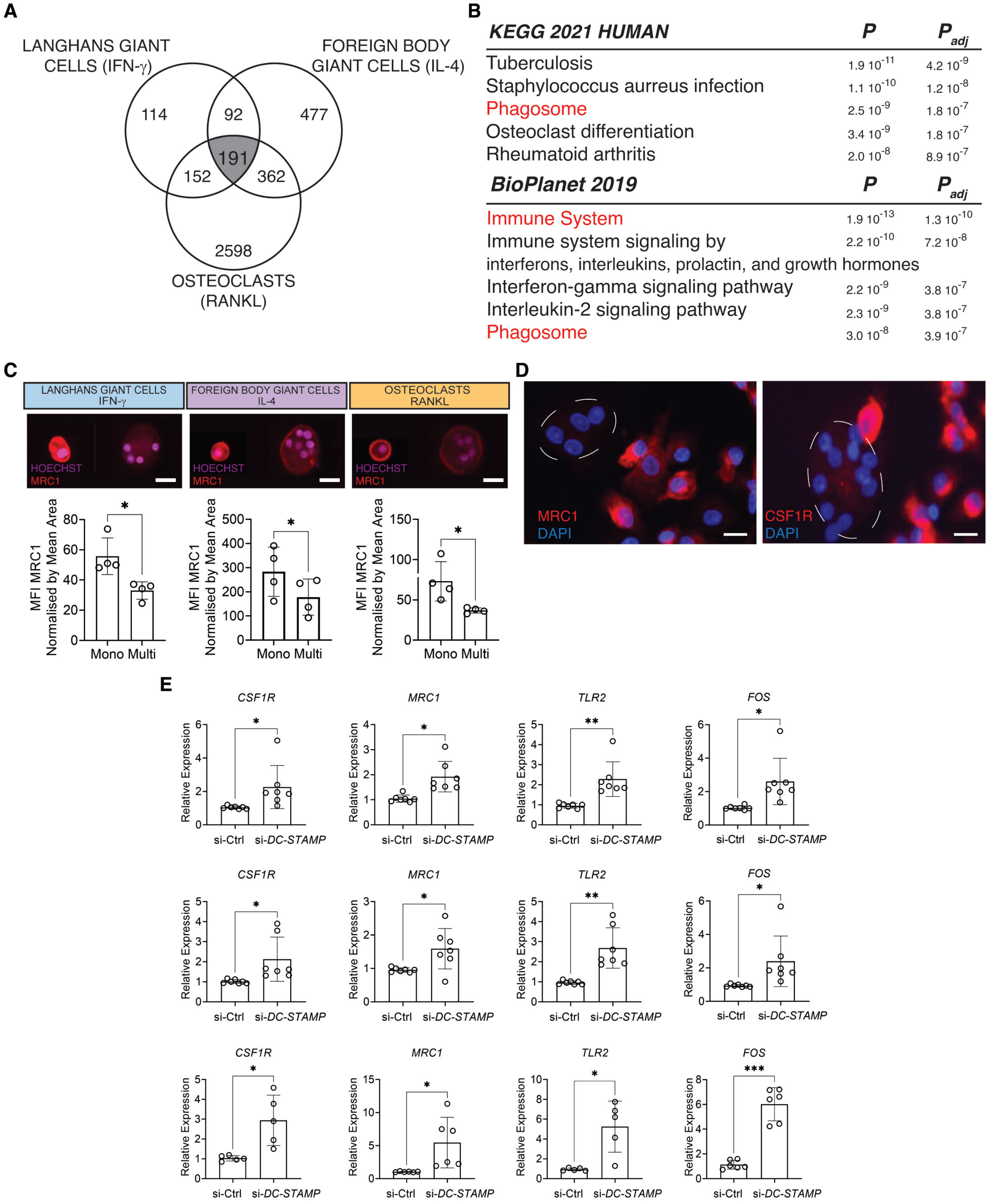

Figure 2.

© 2023 The Authors

Figure 2. Fusion and multinucleation causes the downregulation of a shared mononuclear phagocyte gene signature between the LGCs, FBGCs and osteoclasts.

A Venn diagram showing the commonly downregulated transcripts ($n = 191$) as a result of multinucleation in LGCs, FBGCs and osteoclasts (in gray).
B KEGG and BioPlanet 2019 pathway analyses on the 191 commonly downregulated genes. Pathways in red are shared between KEGG and BioPlanet.
C MRC1 surface marker expression (red) acquired by ImageStream in mononuclear (mono) and multinucleated (multi) LGCs, FBGCs and osteoclasts stained for Hoechst. Bar graphs (lower panel) represent normalized MRC1 mean fluorescence intensity (MFI), measured by ImageStream; $n = 4$ donors.
D Representative MRC1 (left panel) and CSF1R (right panel) immunofluorescence in mononuclear and multinucleated osteoclasts stained for DAPI (blue). Note the dim MRC1 and CSF1R in multinucleated osteoclasts (dashed lines) compared to surrounding mononucleated ones.
E *CSF1R*, *MRC1*, *TLR2* and *FOS* relative expression measured by qRT–PCR for LGCs (upper), FBGCs (middle) and osteoclasts (lower), following the cell membrane fusion regulator DC-STAMP knockdown. si-Ctrl, scrambled siRNA; si-DC-STAMP, DC-STAMP siRNA; at least $n = 5$ donors.

Data information: Error bars are mean ± SD; significance tested by paired *t*-test; $^{*}P < 0.05$; $^{**}P < 0.01$, $^{***}P < 0.001$. Scale bars, 20 μm (C) and 100 μm (D). Source data are available online for this figure.

mononuclear phagocyte signature that is suppressed with multinucleation in differentiated LGCs, FBGCs and osteoclasts.

To validate this fusion-induced suppression of mononuclear phagocyte transcriptome at the protein level, we next focused on two markers of tissue macrophages: CSF1R (Hume & MacDonald, 2012; Stanley & Chitu, 2014) and MRC1 (CD206) (Taylor *et al*, 2005). Surface expression of MRC1 and CSF1R was measured by ImageStream and immunofluorescence (Fig 2C and D). To assess cell surface MRC1 levels, an ImageStream gating strategy was applied to mononuclear and multinucleated cells (Fig EV3B and C). In accordance with the transcriptomic data, multinucleation significantly suppressed the protein levels of both tissue macrophage markers (Fig 2C and D and EV3D). Multinucleation-induced suppression of a core mononuclear phagocyte signature suggests that cell–cell fusion but not lineage-determinants factors are responsible for the loss of macrophage identity. To strengthen these findings, we tested whether direct disruption of cell fusion can recapitulate *MRC1*, *CSF1R*, *TLR2* and *FOS* expression dynamics in multinucleating LGCs, FBGCs and osteoclasts. We thus performed transient RNAi for *DCSTAMP* (Fig EV3E), a plasma membrane regulator of cell fusion in MGCs (Yagi *et al*, 2005, 2006; Pereira *et al*, 2020), and reduced significantly multinucleation in macrophages (Fig EV3F). Strikingly, *DCSTAMP* knockdown reversed the expression of *MRC1*, *CSF1R*, *TLR2* and *FOS* in LGCs, FBGCs and osteoclasts (Fig 2E), confirming that cell fusion and multinucleation trigger the suppression of mononuclear phagocyte gene signature.

Lysosome-dependent iron homeostasis drives human macrophage multinucleation

We next investigated the commonly upregulated genes as a result of multinucleation in LGCs, FBGCs and osteoclasts (Fig 3A). These 66 gene transcripts (Dataset EV3) belong to pathways that include lysosome, iron uptake and transport (Fig 3B). Optimal lysosomal function is a prerequisite for osteoclast function (Lacombe *et al*, 2013) and iron regulates a broad range of macrophage effector functions, including mitochondrial activity and ATP generation (Behmoaras, 2021). Specifically, the upregulated genes in the three cell types include *ATP6V1H*, *ATP6V1D*, *TFRC* and *SLC11A2*. These genes encode for the two subunits of the V1 domain of v-ATPase responsible for ATP-dependent lysosomal acidification (*ATP6V1H* and *ATP6V1D*), transferrin receptor (*TFRC*) and endosomal iron transporter (SLC11A2, also known as NRAM2). Recent evidence suggests that when vacuolar or lysosomal acidification is perturbed, this creates a cytoplasmic iron deficiency and defects in mitochondrial metabolism (Yambire *et al*, 2019; Hughes *et al*, 2020; Weber *et al*, 2020). We thus hypothesized that macrophage multinucleation requires an enhanced lysosomal biogenesis that maintains cytoplasmic iron pools needed to sustain the metabolically demanding process of plasma membrane fusion (Fig 3C and D). To test this hypothesis, we induced lysosomal dysfunction through four different pharmacological means. Ammonium Chloride (NH_4Cl) and hydroxychloroquine sulfate (Hydroxy S) are pH-disrupting lysosomotropic agents, while Bafilomycin A1 (Baf A1) and Concanamycin A (Con A) are vATPase inhibitors (Bowman *et al*, 1988; Drose *et al*, 1993). The use of all four inhibitors of lysosomal function caused a decrease in macrophage fusion in LGCs, FBGCs and osteoclasts (Fig 3E). Importantly, iron replenishment through plasma membrane but not lysosomal compartment ($FeCl_3$ addition) partially rescued the defect in macrophage fusion resulting from lysosomal dysfunction (Fig 3E).

Human LGCs preferentially express B7-H3 and can form granuloma-like clusters *in vitro*

The results above show that multinucleation causes a suppressed macrophage gene signature while lysosomal function upstream iron homeostasis is induced in LGCs, FBGCs and osteoclasts. Having established these shared mechanisms of fusion among these three types of multinucleated macrophages, we next investigated cell type-specific pathways. The uniquely differentially expressed transcripts in LGCs (Fig 4A; Dataset EV4) were enriched for antigen presentation and adaptive immune system pathways (Fig 4B), which are prominent features of infectious granulomas (Pagan & Ramakrishnan, 2018). We found that genes belonging to lipid and atherosclerosis pathway also characterize LGCs (Fig 4B) and these were recently linked to LGC formation (Losslein *et al*, 2021). Given the multicellular immune environment of infectious granulomas and its link with adaptive immune responses, we reasoned that LGCs, but not FBGCs and osteoclasts, can be organized in granuloma-like clusters *in vitro*. In order to test this hypothesis, donor-derived total PBMCs instead of purified monocytes were stimulated with either IFN-γ or IL-4 or RANKL. At day 7 of differentiation of monocyte-lymphocyte co-cultures, MGCs were stained for immunofluorescence analysis. Importantly, granuloma-like clusters were formed in LGC culture conditions, and were absent in FBGCs and osteoclasts cultures (Fig 4C). These LGC-specific granuloma-like clusters contained $CD3^{+}$ T cells, which were absent in FBGCs and osteoclasts (Figs 4D and EV4A). To further strengthen LGC-T cell link, we focused on the LGC-expressed transcript encoding B7-

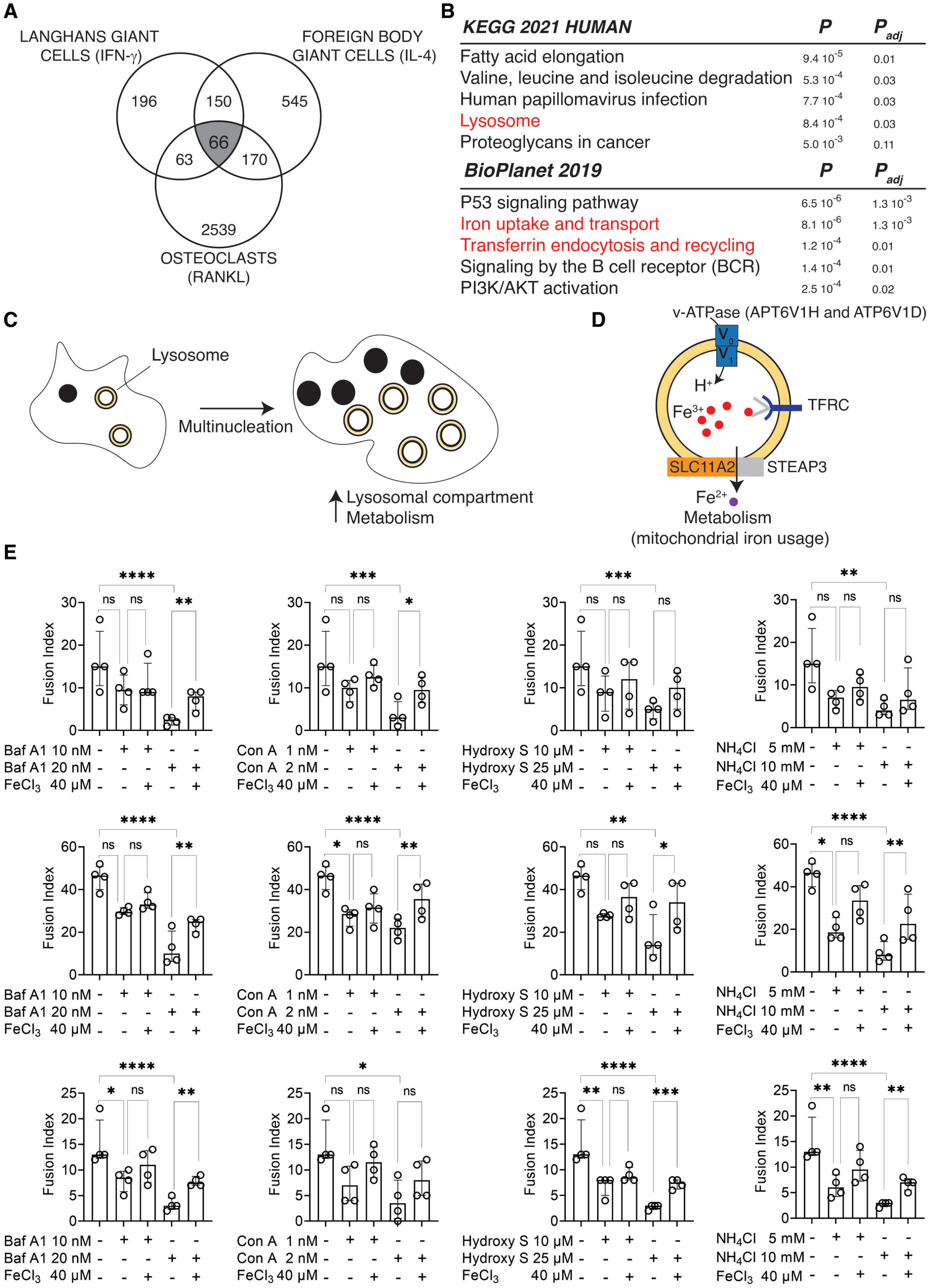

Figure 3.

© 2023 The Authors

Figure 3. Acidic lysosomal pH upstream iron homeostasis is necessary for macrophage fusion.

A Venn diagram showing the commonly upregulated transcripts ($n = 66$) in LGCs, FBGCs and osteoclasts (in gray).
B KEGG and BioPlanet 2019 pathway analyses on the 66 commonly upregulated genes. Pathways in red are connected.
C Macrophage fusion and multinucleation causes an increased lysosomal compartment and cell metabolism.
D The commonly upregulated genes in LGCs, FBGCs and osteoclasts (*ATP6V1H*, *ATP6V1D*, *TFRC* and *SLC11A2*) and their role in the lysosome-iron pathway.
E The effect of lysosomotropic agents (hydroxychloroquine, hydroxy S; ammonium chloride, NH_4Cl) and v-ATPase inhibitors (Bafilomycin A1, Baf A1; Concanamycin A, Con A) on fusion and multinucleation in LGCs (upper panel), FBGCs (middle panel) and osteoclasts (lower panel). To test the effect of lysosome dysfunction on cellular iron, $FeCl_3$ was supplemented. Error bars are median with interquartile range; significance tested by one-way Anova followed by Sídák's multiple comparisons on log transformed data; $n = 4$ donors; *$P < 0.05$; **$P < 0.01$; ***$P < 0.001$; ****$P < 0.0001$.

Source data are available online for this figure.

H3 (also known as CD276), a member of the human B7 family and type I transmembrane protein necessary for T cell activation and IFN-γ production (Chapoval *et al*, 2001). Although *CD276* transcript levels are upregulated in fusing LGCs and osteoclasts (Dataset EV5), we found that multinucleation of LGCs, but not FBGCs and osteoclasts, significantly increased the surface expression of B7-H3 (Fig 4E and EV4B). In summary, IFN-γ-induced macrophage multinucleation leads to the formation of granuloma-like cell clusters containing $CD3^+$ T cells. Human LGCs upregulate B7-H3 upon multinucleation, which may confer them a distinctive advantage for T cell regulation within granulomas.

FBGCs show improved ability of phagocytosis while osteoclasts maximize their mitochondrial activity following multinucleation

We next focused on FBGC-specific transcripts (Fig 5A; Dataset EV6) and interrogated whether they are indicative of functional adaptation in these multinucleated cells. Unsurprisingly, FBGCs were enriched for IL-4 signaling pathway together with VEGF-associated genes (Dondossola *et al*, 2016; Fig 5B). Furthermore, pathways like shigellosis which overlap with the regulation of actin cytoskeleton were suggestive of specialized phagocytic activity of FBGCs (Milde *et al*, 2015). We thus quantified phagocytosis of *staphylococcus aureus*-coated beads in mononuclear and multinucleated human LGCs, FBGCs and osteoclasts. Despite the previously identified loss of macrophage identity, multinucleation significantly induced phagocytosis when normalized to cell size in FBGCs, but not in the other two types of polykaryons (Figs 5C and EV5). As expected, osteoclasts showed a transcriptomic signature robustly associated with mitochondrial activity (i.e. oxidative phosphorylation, TCA cycle and respiratory electron transport) with increased expression of functional markers such as *CTSK* (Fig 6A and B; Dataset EV7). These ATP-generating pathways are indispensable for osteoclasts' unique and energy-consuming bone resorptive function, which we confirmed (Fig 6C). Notably, all 3 types of MGCs were $TRAP^+$ but only osteoclasts showed hydroxyapatite resorption (Fig 6C). To further confirm that multinucleation boosts oxidative phosphorylation in osteoclasts, we quantified oxygen consumption rate (OCAR) and extracellular acidification rate (ECAR) in mononuclear vs. multinucleated osteoclasts and observed that the spare respiratory capacity and ATP production but not glycolysis measurements are increased significantly with multinucleation (Fig 6D and E). Furthermore, osteoclast mitochondria-encoded transcripts were among the genes that showed the largest fold-change upon multinucleation (Fig 1C). The multinucleation-induced increase in *MT-ND1*, *MT-CYTB*, *MT-CO2*, *MT-CO3* expression was not due to increased mitochondrial copy number as normalized expression level of these transcripts show significant upregulation in multinucleated versus mononuclear osteoclasts (Fig 6F and G). Thus, multinucleation induces maximal respiration in osteoclasts, confirming the unique transcriptional signature in mitochondria-related pathways such as oxidative phosphorylation/TCA cycle and respiratory electron transport chain. In summary, macrophage multinucleation causes a shared downregulation of mononuclear phagocyte identity while the lysosome-iron pathway is induced (Fig 6H). These shared pathways coincide with each polykaryon acquiring lineage-dependent specialized functions (Fig 6H).

Discussion

Understanding the consequences of cell–cell fusion in terms of macrophage effector functions has been challenging. A multinucleation-induced 'gain of function' is a theory put forward to explain why osteoclasts, FBGCs and most likely MGCs of adipose tissue are multinucleated (Olona *et al*, 2021). This hypothesis specifies that multinucleated cell state reprograms the cell for a specific effector function that is not present or is less effective in the mononuclear cell state. Supporting evidence for this concept is the increased lysosome numbers and plasma membrane surface in fused cells, allowing proficient degradative and phagocytic activities, respectively. Indeed, osteoclast multinucleation correlates with the resorptive activity of the cell and regulates bone mass (Pereira *et al*, 2020). Similarly, MGCs of the adipose tissue (or adipoclasts) can phagocytose lipid remnants more efficiently when they are multinucleated (Braune *et al*, 2021), a feature that is shared with FBGCs which are also specialized in the uptake of relatively large particles (Milde *et al*, 2015). However, these findings do not explain why LGCs are multinucleated in granulomas as these MGCs have been described to allow persistence to *mycobacterium* rather than exerting bactericidal activities (Gharun *et al*, 2017). Regarding osteoclasts, although fusion confers resorptive function, recent evidence points toward a previously unappreciated plasticity of mature osteoclasts during inflammation (Madel *et al*, 2019, 2020). Hence the functional advantage of macrophage fusion and the underlying pathways are complex in osteoclasts and remain incompletely understood in LGCs and FBGCs.

One reason for this gap in knowledge is the lack of consensus for determining cell culturing conditions involving LGCs and FBGCs *in vitro*. Furthermore, the reliable isolation of pure populations of mononuclear and multinucleated mature macrophages has hindered the side-by-side comparison of MGC post-fusion events in LGCs,

FBGCs and osteoclasts in humans. Here we compared mature and purified mononuclear and multinucleated macrophages, in order to minimize the combined effects of M-CSF and the lineage inducer (IFN-γ, IL-4, RANKL) and to study pathways resulting from cell–cell fusion. We show that LGCs, FBGCs and osteoclasts are formed through cell–cell fusion, though we cannot exclude incomplete cytokinesis events (Takegahara *et al*, 2016). We propose that macrophage cell–cell fusion and multinucleation triggers common effector

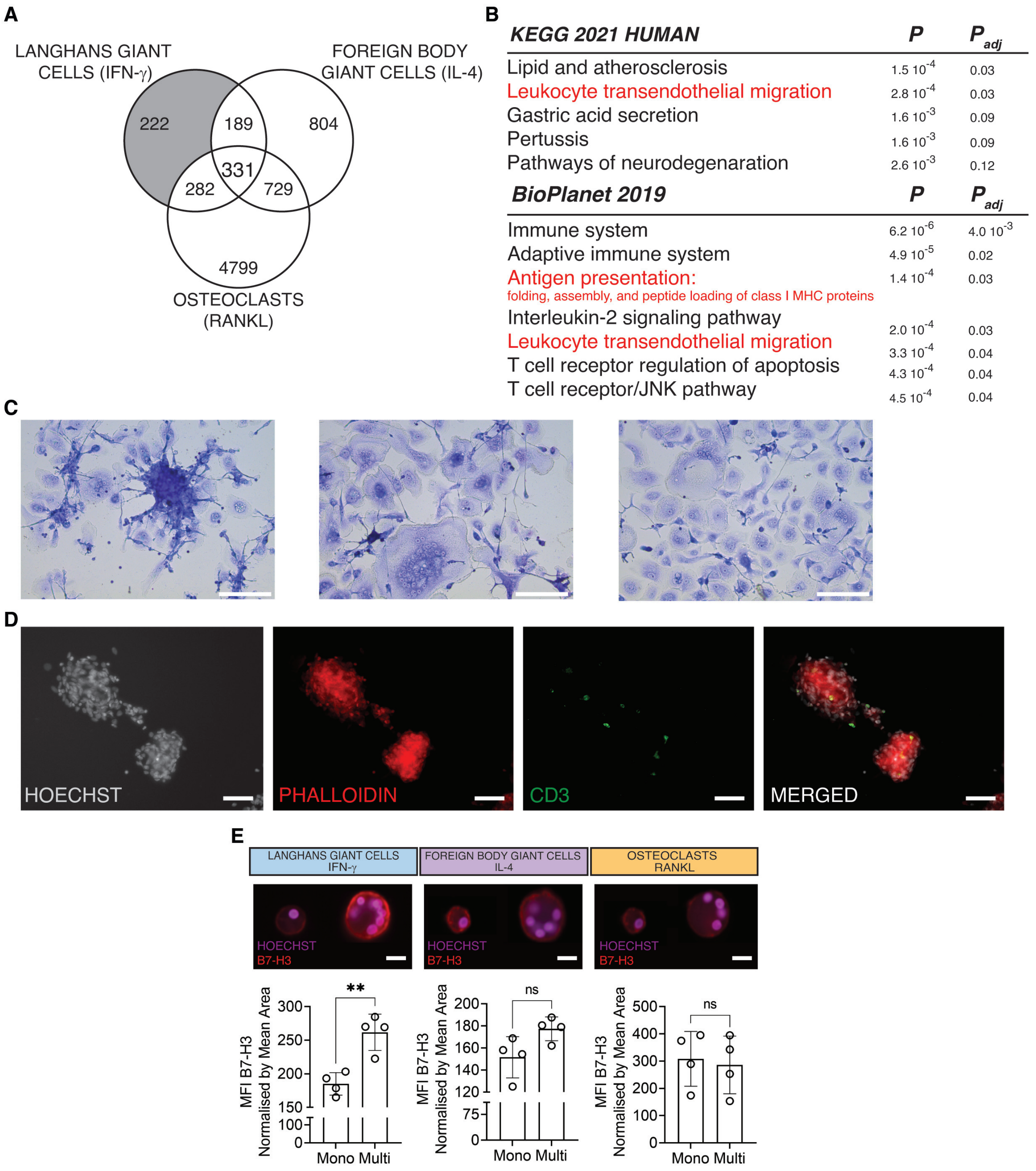

Figure 4.

 © 2023 The Authors

Figure 4. LGCs form granuloma-like clusters and show increased membrane expression of B7-H3.

A Venn diagram showing the uniquely differentially expressed transcripts in LGCs (in gray).
B KEGG and BioPlanet 2019 pathway analyses on the differentially expressed transcripts in LGCs only. Pathways in red are in common in KEGG and BioPlanet or relevant for LGC function.
C Giemsa stained LGCs (left), FBGCs (middle) and osteoclasts (right) differentiated from PBMCs. Note the cell cluster formation in LGCs only.
D CD3 immunofluorescence (green) in PBMC-derived LGCs. Hoechst (gray) and phalloidin (red) staining show the nuclei and cytoskeleton, respectively. Merged (CD3, Hoechst, phalloidin) image shows $CD3^+$ T cells in the granuloma-like clusters.
E B7-H3 surface marker expression (red) acquired by ImageStream in mononuclear and multinucleated LGCs, FBGCs and osteoclasts stained for Hoechst. Bar graphs (lower panel) show normalized B7-H3 mean fluorescence intensity (MFI), measured by ImageStream; $n = 4$ donors. Error bars are mean ± SD; significance tested by paired t-test; $^{**}P < 0.01$; ns, non-significant.

Data information: Scale bars, 100 μm (C and D) and 20 μm (E).
Source data are available online for this figure.

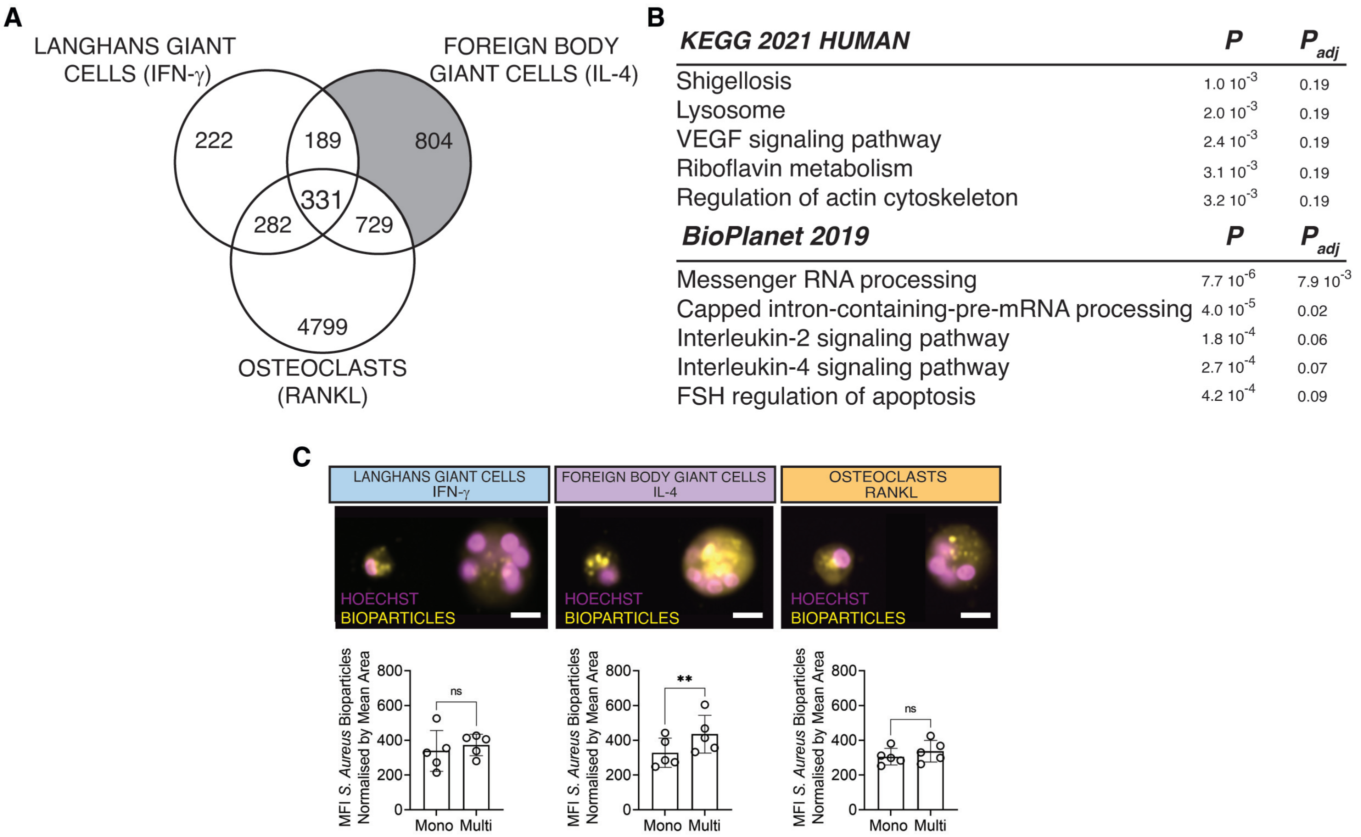

Figure 5. FBGCs show a distinctively enhanced phagocytic capacity.

A Venn diagram showing uniquely differentially expressed transcripts in FBGCs (in gray).
B KEGG and BioPlanet 2019 pathway analyses on differentially expressed transcripts in FBGCs only.
C Phagocytosis quantified by ImageStream analysis in LGCs, FBGCs and osteoclasts. Representative images showing the uptake of *S. aureus* bioparticles (yellow) in mononuclear (mono) and multinucleated (multi) cells. Bar graphs (lower panel) show normalized *S. aureus* bioparticles mean fluorescence intensity (MFI) measured by ImageStream; $n = 5$ donors. Error bars are mean ± SD; significance tested by paired t-test; $^{**}P < 0.01$; ns, non-significant. Scale bars, 20 μm.

Source data are available online for this figure.

pathways alongside activation of MGC-specific reprogramming that is intimately linked to the lineage-determining factors.

TNF and TNF receptor super-families include RANKL, RANK and osteoprotegerin, and are experimentally proven regulators of osteoclast formation *in vitro* and *in vivo* (Walsh & Choi, 2014; Takegahara *et al*, 2022). On the other hand, IFN-γ is a T-helper cell type 1 (Th1) cytokine, previously demonstrated to be an efficient inducer of LGCs from human monocytes (Weinberg *et al*, 1984; Sakai *et al*, 2012). *In vivo*, IFN-γ is found in human tuberculosis and leprosy granulomas (Ma *et al*, 2021; Gideon *et al*, 2022) and in non-infectious granulomatous disease such as sarcoidosis (Ramstein *et al*, 2016; Damsky *et al*, 2022) and Blau syndrome (Wouters

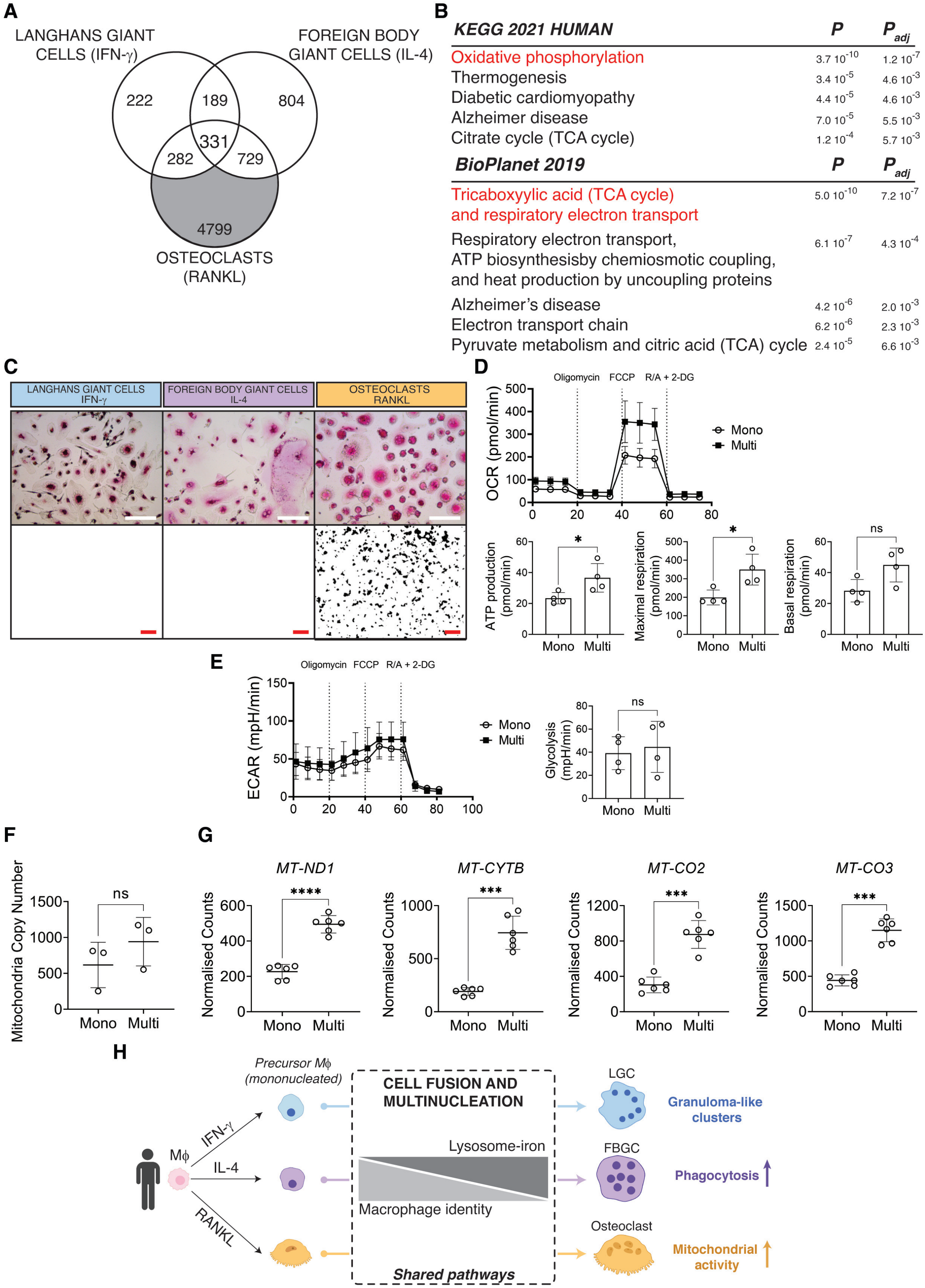

Figure 6.

© 2023 The Authors

Figure 6. Osteoclasts show a distinctively increased mitochondrial activity.

A Venn diagram showing uniquely differentially expressed transcripts in osteoclasts (in gray).
B KEGG and BioPlanet 2019 pathway analyses on differentially expressed transcripts in osteoclasts only. Pathways in red overlap between KEGG and BioPlanet.
C TRAP immunohistochemistry (upper panel) and hydroxyapatite resorption (lower panel) in LGCs, FBGCs and osteoclasts. Note the presence of $TRAP^+$ MGCs in all cell types and hydroxyapatite resorption only in osteoclasts.
D Oxygen consumption rate (OCR, upper panel) and its related readout (lower panel) measured by extracellular flux analysis in sorted mononuclear and multinucleated osteoclasts; $n = 4$ donors.
E Extracellular acidification rate (ECAR, left) and glycolysis (right) in mononuclear and multinucleated osteoclasts; $n = 4$ donors.
F Mitochondria copy number measured by qPCR in mononuclear and multinucleated osteoclasts; $n = 3$ donors.
G Expression of mitochondrial transcripts in sorted mononuclear and multinucleated osteoclasts after normalization by mitochondrial copy number; $n = 6$ donors.
H Cartoon illustrating human macrophage multinucleation, its shared and lineage-specific pathways.

Data information: Error bars are mean ± SD; significance tested by paired *t*-test; $*P < 0.05$; $***P < 0.001$; $****P < 0.0001$; ns, non-significant. Scale bars, 40 μm (C, upper panel) and 1 mm (C, lower panel).
Source data are available online for this figure.

et al, 2014). All these conditions are associated with the presence of LGCs and T cells (either Th1 or Th17) within the granulomas. Human subjects with defects in the IFN-γ signaling pathway develop mycobacterial infections without formation of granulomas, demonstrating the need of IFN-γ signaling for LGC-containing granuloma formation *in vivo* (Saunders & Britton, 2007). Similarly, IL-4 has been shown to induce FBGCs from human monocytes *in vitro* and *in vivo* (Kao *et al*, 1995; McNally & Anderson, 1995; Sheikh *et al*, 2015) even though single cell transcriptomics analyses of foreign body reactions have shown that the immune cell landscape may depend on whether the biomaterial is biological matrix or synthetic (Sommerfeld *et al*, 2019); the latter being associated with a prominent role of IL-17 (Chung *et al*, 2020). Thus, both IFN-γ and IL-4 have been shown to promote MGC formation *in vitro* and the two cytokines show niche-specific presence in human tissues during inflammatory disease involving MGCs. While macrophage polarization as a result of RANKL, IFN-γ and IL-4 stimulation is well-documented (Gordon, 2003; Ivashkiv, 2018; Takayanagi, 2021), our results show an additional cell reprogramming triggered by multinucleation, suggesting that this advanced maturation step is essential for the cell-specific activity of MGCs *in vivo*.

We report that macrophage multinucleation causes a downregulation of mononuclear phagocyte identity in LGCs, FBGCs and osteoclasts. This process consists of suppression of expression of major macrophage receptors (*CSF1R*, *MRC1*, *CD163*, *TLR2*, *CD74*, *IFNGR1*) and transcription factors (*MAFB*, *FOS*, *JUNB*). AP-1 transcription factors (*FOS*, *JUNB*) are involved in monocyte lineage specification (Friedman, 2007; Heinz *et al*, 2010) and FOS is a well-described osteoclast lineage determinant transcription factor (Grigoriadis *et al*, 1994). Given the role MAFB in tissue-resident macrophage lineage commitment (Geissmann *et al*, 2010), the results overall suggest a downregulation of monocyte/macrophage lineage identity caused by multinucleation. The intracellular signaling pathways leading to this 'extinction' phenotype remains to be determined. Since multinucleation of macrophages is associated with de-phosphorylation of kinases belonging to cJun NH2-terminal kinase (JNK) pathway such as Map3k1 (Rotival *et al*, 2015), one can hypothesize the involvement of JNK and downstream transcriptional activity of AP-1 as a common suppressed signaling pathway associated with multinucleation in LGCs, FBGCs and osteoclasts. Multinucleation-induced suppression of core macrophage identity corroborates with previous findings in mice. First, the suppression of macrophage-specific transcripts such as Mafb and Csf1r have been shown in multinucleated mouse macrophages when compared to mononuclear cells cultured with M-CSF and a synthetic ligand for TLR2/6 (Herrtwich *et al*, 2016). Second, the downregulation of AP-1 was shown in BMDMs infected with Mtb (Roy *et al*, 2018). Finally, work performed more than 50 years ago by Siamon Gordon and Zanvil A. Cohn on mouse macrophage-melanocyte heterokaryons and macrophage–macrophage homokaryons showed alteration of macrophage phagocytosis in fused cells, though the changes were more drastic in heterokaryons (Gordon & Cohn, 1970, 1971). In that sense, one limitation of our study is the lack of comparison between homologous and heterologous macrophage fusion since the latter can be relevant *in vivo* with the finding showing fusion of circulating blood monocytic cells with long-lived osteoclast syncytia in mice (Jacome-Galarza *et al*, 2019). In cancer, the relevance of fusion between neoplastic cells and macrophages has been linked to tumor heterogeneity with acquired phenotypes in fused cells (Gast *et al*, 2018).

Macrophage multinucleation activates the lysosome-controlled cellular iron homeostasis in LGCs, FBGCs and osteoclasts. Our results suggest that multinucleation induces lysosomal biogenesis and capitalize on cytoplasmic and mitochondrial iron pools to sustain mitochondrial activity for increased energy demands. MGCs have been described having increased numbers of lysosomes decades ago (Sutton & Weiss, 1966) and several lines of evidence link v-ATPase-dependent lysosomal function to osteoclast multinucleation and function (Lee *et al*, 2006; Duan *et al*, 2016). Similarly, TFRC-mediated iron uptake induces mitochondrial respiration regulating osteoclast differentiation, mature osteoclast function and bone mass (Ishii *et al*, 2009; Das *et al*, 2022). Hence, lysosomal biogenesis coupled with iron homeostasis is likely to be a 'metabolic booster' for specialized functions in multinucleated macrophages. Iron is essential for mitochondrial OXPHOs and TCA cycle activity in macrophages (Pereira *et al*, 2019) and further studies on the lysosome-iron axis and its regulatory pathways will clarify the shared mechanistic insights of post-fusion metabolic reprogramming in MGCs.

Our comparative analysis identified MGC-specific transcriptomic signatures and MGC-associated phenotypes *in vitro*. The cross-lineage analysis of IFN-γ and IL-4 differentiated macrophages revealed that multinucleated LGCs and FBGCs maintain a large

© 2023 The Authors

transcriptomic signature related to IFN-γ and IL-4 when compared to their mononuclear counterparts. This suggests that lineage-specific gene expression and epigenetic landscape define the niche-specific activities of MGCs. For instance, IFN-γ-related pathways favor T cell interaction in LGCs, while IL-4 signaling seems to potentiate phagocytic properties of FBGCs. Importantly, both the transcriptomics analysis and functional assays confirm the key known characteristics of each cell type: granuloma-like cluster formation (LGCs), enhanced phagocytosis (FBGCs), heightened mitochondrial activity that fuels bone resorption (osteoclasts). These findings combined with the specific morphological features of these cells (e.g. ring-shaped nuclei in LGCs) validate the usage of IFN-γ and IL-4 for the *in vitro* generation of human LGCs and FBGCs, respectively. Whether MGC-specific nuclear arrangements and/or numbers are indicative of specialized function is currently unclear. Intracellular nuclei arrangement is likely to be important for the sealing zone formation in a polarized bone-resorbing osteoclast. Furthermore, whether distinct transcriptional activities are assigned to different nuclei of the MGC also remain to be tested. Recent elegant work performed in multinucleated skeletal myofibers suggest transcriptional heterogeneity among the different nuclei of the polykaryon (Petrany *et al*, 2020).

The specific upregulation of surface B7-H3 by LGCs suggest that this transmembrane protein is also involved in LGC-T cell crosstalk in granulomatous disease. B7-H3 mRNA is expressed by human mature osteoclasts (Oh *et al*, 2021) and our results showed that multinucleation of LGCs but not osteoclasts showed an upregulation of membrane levels of B7-H3. Hence one explanation for the functional relevance of multinucleation in LGCs could be the spatial positioning of these MGCs expressing B7 family members such that they regulate T cells within granulomas. FBGCs have been previously shown to have minimal resorptive activity but they cannot resorb bone (ten Harkel *et al*, 2015). Regarding the enhanced phagocytic activity of FBGCs when compared to osteoclasts and LGCs, the exact surface receptors used by FBGCs in phagocytosis of *S. aureus* bioparticles remain to be identified. Given that all three types of MGCs show a downregulation of transcripts that belong to Fc receptors and complement C1Q family, the enhanced phagocytosis in these cells seems paradoxical. FBGC phagocytosis is a complex process including non-canonical phagocytosis pathways involving contribution of membrane area and endoplasmic reticulum (McNally & Anderson, 2005; Milde *et al*, 2015). Mitochondrial function, and more specifically electron transport chain activity regulates osteoclast function (Jin *et al*, 2014; Lemma *et al*, 2016). Our results suggest that cell–cell fusion and multinucleation enhance the mitochondrial activity required for resorptive activity in osteoclasts. Since glycolysis remain unchanged, the energy sources for the increased maximal respiration in multinucleated osteoclasts remain to be determined. As previously reported, we found that, in addition to osteoclasts, MGCs are also TRAP$^+$ (Brooks *et al*, 2019) but among the three types of human MGCs, only osteoclasts can show resorptive activity toward hydroxyapatite.

From a clinical perspective, since lysosome-regulated intracellular iron homeostasis appears to be a general condition for macrophage multinucleation across different tissues, its blockade may hold therapeutic potential. However, it is still unclear whether granulomatous disease can benefit from targeting LGC fusion. For non-granulomatous inflammatory diseases, inhibiting MGC formation by targeting lysosomes may be a therapeutic avenue. This approach would have the benefit of avoiding FBGC-related adverse effects during foreign body reaction. v-ATPase inhibitors have been previously proposed to inhibit osteoclast activity and bone resorption (Qin *et al*, 2012) so their selective targeting in the lysosomal compartment may be generalized to other MGCs such as FBGCs. Furthermore, iron depletion from macrophages can be therapeutically beneficial given the association between white adipose tissue (WAT) macrophage mitochondrial iron and systemic metabolism (Joffin *et al*, 2022). Considering the presence of MGCs in the WAT (known as crown-like structures or adipoclasts; Olona *et al*, 2021), targeting iron to prevent fusion of WAT macrophages hold potential. Similarly, iron-rich Kupffer cells form hepatic crown-like structures, driving inflammation during non-alcoholic steatohepatitis (Kanamori *et al*, 2021), though liver crown-like structures seem to be more macrophage aggregates rather than MGCs (Ioannou *et al*, 2013; Itoh *et al*, 2013). Finally, the *in vitro* results presented here require *in situ* confirmation in the context of human pathology involving MGCs.

In summary, we showed that cell–cell fusion and multinucleation of LGCs, FBGCs and osteoclasts reset differentiated macrophages by at least two means: loss of monocyte/macrophage signature and triggering of lysosome-regulated intracellular iron metabolism. These common pathways coincide with cell type-specific gain of function that characterizes each polykaryon. We thus propose that macrophage fusion is a major determinant in shaping the context-dependent activity of MGCs.

Materials and Methods

MGC generation from human donors

Human monocyte-derived macrophages were separated from healthy donor buffy coats by centrifugation through Histopaque 1077 (Sigma-Aldrich, Cat # H8889) gradient. Following Histopaque separation, peripheral blood mononuclear cells (PBMCs) were re-suspended in RPMI for osteoclasts (Life Technologies) and MEM-REGA 3 (1×) + 10% FBS (Life Technologies) for LGCs and FBGCs. For LGCs and FBGCs, monocytes were purified using a CD14 positive selection kit (StemCell Technologies, Cat # 17858). Monocytes (3.6 × 10^6 cells) were seeded in 1 well Labtek chamber slides (ThermoFisher Scientific, Cat # 177372) and stimulated with 20 ng/ml M-CSF (PeproTech) for 3 days. At day 3, cells were differentiated with M-CSF (20 ng/ml, PeproTech) and IFN-γ (25 ng/ml, PeproTech, LGC differentiation) or IL-4 (10 ng/ml, PeproTech, FBGC differentiation) for further 4 days with the cell culture media changed every 2 days. For osteoclast generation, monocytes were purified by adherence for 1 h at 37°C, 5% CO_2. The monolayer was washed 3 times with HBSS to remove non-adherent cells and monocytes were differentiated into macrophages for 3 days in RPMI media +10% FBS containing 30 ng/ml M-CSF (PeproTech) and for further 4 days in media containing 30 ng/ml RANKL (PeproTech) and M-CSF. For any treatment, samples were randomly allocated into controls and treated cells. Studies on human donors were approved by Imperial College London (NHS Blood and Transplant—NCI R148) and KU Leuven (Rode Kruis Mechelen—RKOV19006). The rules and legislations were based on the Declaration of Helsinki and the Department of Health and Human Services

© 2023 The Authors

Belmont Report (informed consent for participation of human subjects in medical and scientific research).

Mononuclear and multinucleated macrophage sorting and purity

IFN-γ, IL-4 and RANKL-generated respective LGCs, FBGCs and osteoclasts were detached using Accutase (StemCell Technologies, Cat # 07920). Cells were labeled with 10 μg/ml Hoechst 33342 (Sigma-Aldrich, Cat # B2261) in cell sorting buffer containing PBS, 1% FBS (Sigma-Aldrich, Cat # F7524) and 2 mM EDTA for 40 min at 37°C to stain the DNA. Cells were then briefly centrifuged and re-suspended in ice-cold sorting buffer and kept on ice. Before FACS sorting, cells were filtered through a 70 μm nylon mesh (VWR, Cat # 732-2758). In order to sort mononuclear and multinucleated LGCs and FBGCs, FSC-A (logarithmic) versus Hoechst was plotted (Fig EV1B) and gating purity was further assessed by ImageStream (Fig 1B, lower panel). After sequential purity checks in at least 10 donors, a gating for mononuclear and multinucleated LGCs and FBGCs was selected (Fig EV1B) based on 85–95% purity achieved by ImageStream in mononucleated and multinucleated cells. Cells were sorted on a BD FACSAria Fusion (BD Bioscience) using a 100 μm nozzle at a flow rate of 2,000 events. Sorted cells were collected in FBS. For osteoclasts, after doublet exclusion, singlets with 1 nucleus (mononuclear cells) and more than 2 nuclei (multinucleated cells) were selected by FSC-A and SSC-A and the number of nuclei based on the Hoechst staining (Fig EV1C). Sorted mononuclear and multinucleated cells from each donor were run through the ImageStream that allowed direct visualization of the number of nuclei; each cell population showed > 95% purity.

RNA extraction, library preparation and data analysis

Total RNA was extracted from sorted mononuclear and multinucleated LGCs, FBGCs and osteoclasts using Trizol (Invitrogen) and RNeasy mini kit (Qiagen) according to manufacturer's instructions. Total RNA quality and concentration was analyzed using a NanoDrop 1000 spectrophotometer (ThermoFisher Scientific) and verified using Qubit meter (Invitrogen). Total RNA was analyzed by Agilent 2100 Bioanalyzer (Agilent Tech Inc.) and RNA integrity number (RIN) values were ≥ 9.0 for all samples. Sequencing libraries for osteoclasts were prepared using NEBNext Ultra II Directional RNA Library Prep kit (Illumina). Briefly, RNA was purified and fragmented using poly-T oligo-attached magnetic beads using two rounds of purification followed by the first and second cDNA strand synthesis. Next, cDNA 3′ ends were adenylated and adapters ligated followed by 11 cycles of library amplification. The libraries were size selected using AMPure XP Beads (Beckman Coulter), purified and library quality was checked using Agilent 2100 Bioanalyzer. Samples were randomized to avoid batch effects and multiplexed libraries were run on a single lane (8 samples/lane) of the HiSeq 2500 platform (Illumina) to generate 100 bp paired-end reads. Sequencing libraries for LGCs and FBGCs were prepared with the Lexogen QuantSeq 3′ mRNA-Seq Library prep kit according to the manufacturer's instructions. Samples were indexed to allow for multiplexing. Library quality and size range was assessed using Agilent 2100 Bioanalyzer. Samples were randomized to avoid batch effects and multiplexed libraries were run on a single lane (8 samples/lane) of the HiSeq 4000 platform (Illumina) to generate 50 bp single-end reads. Sequencing adapters were removed using Trimmomatic (v.0.36) and the reads quality was checked using FastQC (v.0.11.2) before and after trimming. Reads were aligned to the human genome (GRCh38.primary_assembly.genome.fa; annotation: gencode.v26.annotation.gtf) using HISAT2 package (v.2.1.0). Reads mapping to multiple loci in the reference genome were discarded. Mapping quality, read distribution, gene body coverage, GC content and rRNA contamination, were checked using picard (v.2.6.0) software. Gene level read counts were computed using HT-Seq-count (v2.7.14, annotation: gencode.v26.annotation.gtf). Genes with < 10 aligned reads across all samples were filtered out as lowly expressed genes. Differential gene expression analysis between groups was performed using DESeq2 (v.1.14.1) and significantly differentially expressed genes were reported using fold-change at 1.5 times and below 5% Benjamini-Hochberg (BH) adjusted *P*-value. In order to visualize the similarities between samples, unsupervised hierarchical clustering and principal component analysis (PCA) were performed using pcaExplorer (v.2.6.0, https://github.com/federicomarini/pcaExplorer) and pheatmap (v 1.0.10, https://CRAN.R-project.org/package=pheatmap) packages respectively. Volcano plots of differentially expressed genes were generated using enhanced volcano plot (v. 1.14.0, https://github.com/kevinblighe/EnhancedVolcano) package. All raw RNA-seq data processing steps were performed in Cx1 high-performance cluster computing environment, Imperial College London. Further analyses were conducted in R/Bioconductor environment v.3.4.4 (http://www.R-project.org/). Transcripts that are commonly up/down-regulated or uniquely differentially expressed were used for pathway analysis (EnrichR https://maayanlab.cloud/Enrichr/) with corresponding *P*-values (Fisher exact test) and adjusted *P*-values (Benjamini-Hochberg method).

IncuCyte live cell imaging

At day 0, monocytes were resuspended in PBS and labeled with Incucyte Cytolight rapid green or red reagent for live cell cytoplasmic labelling (0.750 μM, Sartorius, Cat # 4705 and Cat # 4706) for 20 min at 37°C, 5% CO_2 and washed with cold medium (MEM-REGA 3 (1X), Life Technologies). Labeled monocytes (0.5 × 10^6 cells) were cultured at a ratio of red:green (1:1) in 8-well chamber slides (ThermoFischer Scientific, Cat # 154534) and stimulated with M-CSF (20 ng/ml, PeproTech, UK) for 3 days. Next, cells were stimulated with M-CSF (20 ng/ml, PeproTech, UK) in combination with IFN-γ (25 ng/ml, PeproTech, LGCs) or IL4 (10 ng/ml, PeproTech, FBGCs) or RANKL (30 ng/ml, PeproTech, osteoclasts). Imaging was performed with Incucyte live cell imaging system (Essen Biosciences) every 4 h for the first 3 days and every 3 min after stimulation with either IFN-γ or IL-4 or RANKL with a magnification of 20× (37°C, 5% CO_2). Phase images were acquired for every experiment and for fluorescent imaging, the acquisition times were 300 and 400 ms for green and red channels, respectively.

ImageStream analysis

At day 7 of MGC formation, differentiated LGCs, FBGCs and osteoclasts, were detached using Accutase (StemCell Technologies, Cat # 07920). Cells were stained with 10 μg/ml Hoechst 33342 (Sigma-Aldrich, Cat # B2261) in PBS supplemented with 1% FBS and 2 mM EDTA for 40 min at 37°C to stain for DNA. Cells were then briefly centrifuged, re-suspended in ice-cold sorting buffer, incubated with FcR block (Miltenyi Biotec, Cat # 130059901) and stained with human anti-MRC1 or anti-B7-H3 (APC, Biolegend, MRC1, Cat #

© 2023 The Authors

321110 and B7-H3, Cat # 351006). Stained cells were filtered through a 70 μm nylon filter (VWR, Cat # 732-2758) and analyzed with ImageStream (Amnis Corporation) with fluorescence measurements at 375 nm and 642 nm for Hoechst 33342 and MRC1/B7-H3, respectively. For each blood donor, a number of 50,000–100,000 cells were acquired. Based on their size (Area and Aspect Ratio) and number of nuclei, cells were divided into mononuclear and multinucleated cells (2+ nuclei) (Fig EV3B). The mean fluorescence intensity of the specific markers was calculated and normalized by the mean area of the gated populations. Results were analyzed with Ideas v5 Software (Amnis Corporation).

Immunofluorescence

MGCs were culture in 8 well-plates (ThermoFisher Scientific, Cat # 177402) for 7 days. Cells were fixed in ice-cold methanol at −20 for 15 min, washed three times with PBS and blocked for 1 h in PBS containing 5% normal goat serum (Cell signaling, Cat # #5425) and 0.3% Triton X-100. The cells were then incubated overnight at 4°C with the primary antibody in blocking buffer. The antibodies used are as follows: rabbit monoclonal anti-CSF1R antibody (Cell Signaling Technologies, Cat # 67455S; 1:100), rabbit polyclonal anti-MRC1 antibody (Abcam, Cat # ab64693; 1:500). For visualization, the secondary Alexa 455-conjugated anti-rabbit IgG (Cell signaling Technologies, Cat # 4413S; 1:500) was added to the blocking buffer for 1 h at RT. Nuclei were stained in the Prolong Gold AntiFade Reagent with DAPI mounting medium (Cell Signaling Technologies, Cat # 8961S) and mounted under glass. Images were taken using epi-fluorescent Leica DM4B microscope and the raw fluorescence intensity was acquired using the ImageJ software 1.53. Multinucleated and mononuclear cells were visually identified based on their DAPI staining and multinucleated cells were delineated manually and their relative intensity of fluorescence (CSF1R and MRC1) was measured.

For CD3 immunofluorescence, prior to fixation, cells were washed two times with PBS to remove non-sticky cells. Next, cells were fixed in PBS, Hank's balanced salt solution (HBSS Ca^{2+}/ Mg 10×), BSA, sodium bicarbonate ($NaHCO_3$) supplemented with 4% Paraformaldehyde (PFA, 40%, carl roth®) in 8-well plates (ThermoFisher Scientific, Cat # 177402). Cells were then washed twice and incubated 1 h at RT in PFA (40%, carl roth®). Next, cells were rinsed thoroughly in HBSS buffer (PBS, HBSS Ca^{2+}/ Mg 10×, BSA, $NaHCO_3$) for 30 s and left to dry overnight at RT. Cells were then incubated 1 h at RT in permeabilization buffer containing HBSS and 0.1% Triton-X. After washing, the fixed cells were incubated in blocking buffer (HBSS, FCS [10%], fragment crystallizable receptor [FcR] block [5%]) at RT for 2 h. Following the blocking step, CD3-PE (Biolegend, Cat # 300456) was added and the cells were left for 2.5 h in the dark. Hoechst 33342 (Sigma Aldrich) and Phalloidin (Invitrogen) were used to stain the nuclei and cytoskeleton, respectively. The plates were incubated for 1 h at RT and covered from light. Thereafter, the slides were rinsed in HBSS buffer, dried and covered with few drops of diamond mounting medium (Invitrogen, Cat # P36961) were added before microscopy analysis (20× magnification, Zeiss Axiovert 200 M, Carl Zeiss).

DC-STAMP siRNA transfection

siRNA transfection of primary human macrophages was performed based on the previously published protocols (Papathanassiu et al, 2017; Pereira et al, 2019). Monocytes (3.6 × 10^6 cells) were first seeded and stimulated with M-CSF for 3 days (20 ng/ml, PeproTech) in 1-well chamber slides (ThermoFisher Scientific, Cat # 177372) (72 h, 37°C, 5% CO_2). Macrophages were then transfected using Dharmafect 1 (Dharmacon, Cat # T-2001-03) diluted in OPTIMEM medium (1:50, Invitrogen) with human *DC-STAMP* siRNA (100 nM, Dharmacon) and non-targeting scrambled siRNA used as a control. After 8 h, cells were washed and differentiated into MGCs with stimulatuion with either IFN-γ (25 ng/ml, PeproTech) or IL-4 (10 ng/ml, PeproTech) or RANKL (30 ng/ml, PeproTech) in presence of M-CSF (20 ng/ml, PeproTech) containing media for 2 days. The effect of *DC-STAMP* knockdown was subsequently measured by quantitative RT–PCR (qRT–PCR) in LGCs and FBGCs (Fig EV3E). For osteoclasts, DC-STAMP si-RNA transfection was performed in Pereira et al (2020) and the cDNA was used for the described qPCR measurements.

Quantitative RT–PCR

Total RNA was extracted using the Trizol reagent (Invitrogen) according to the manufacturer's instructions. For osteoclasts, complementary DNA (cDNA) was synthesized using iScript cDNA Synthesis Kit (Bio-Rad). A total of 10 ng cDNA for each sample was used and all qRT–PCR reactions were performed on a ViaA 7 Real-Time PCR System (Life Technologies) using Brilliant II SYBR Green QPCR Master Mix (Agilent). Results were analyzed by the comparative Ct method using ViiA 7 RUO software, and each sample was normalized relative to *HPRT* expression. For LGCs and FBGCs, complementary DNA (cDNA) was prepared using Superscript II reverse transcriptase (Invitrogen, Cat # 4311235) and random primers (Invitrogen, Cat # 4319979). qRT–PCR was performed using a TaqMan gene expression assay (Applied Biosystems) on a 7500 Real-Time PCR System Apparatus. Results were analyzed by the comparative C_t method and each sample was normalized to *HPRT* expression levels. Primers used are listed in Table 1.

Lysosomal function, iron supplementation and MGC fusion

Monocytes were seeded and stimulated with M-CSF (20 ng/ml, PeproTech) in 8-well chamber slides (ThermoFisher Scientific, Cat # 177402) for 3 days (37°C, 5% CO_2). Cells were the re-incubated with M-CSF (20 ng/ml, PeproTech) in combination with either IFN-γ (25 ng/ml, PeproTech) or IL-4 (10 ng/ml, PeproTech) or RANKL (30 ng/ml, PeproTech). Simultaneously, cells were incubated with either Bafilomycin A1 (Baf A1, Sanbio B.V., Cat # 11038-500), Concanamycin A (Con A, Sigma-Aldrich, Cat # 27689), Hydroxychloroquine sulfate (Hydroxy Sulfate, Sigma-Aldrich, Cat # 379409) and Ammonium chloride (NH4Cl, Sigma-Aldrich, Cat # A9434). As part of the rescue experiment, iron chloride ($FeCl_3$, Sigma-Aldrich, Cat # 451649) was added to some cells at day 4 and cells were incubated for further 2 days at 37°C, 5% O_2, after which Giemsa staining was performed to measure fusion index using ImageJ. The fusion index (quantified in a blinded way) equals to the percentage of [number of nuclei (> 3)]/[total number of nuclei] in randomly selected microscopy fields.

Phagocytosis by ImageStream

Differentiated LGCs, FBGCs and osteoclasts were detached using Accutase (StemCell Technologies, Cat # 07920) and were filtered

© 2023 The Authors

Table 1. Primers used in qRT–PCR experiments.

CSF1R	PROBE	5′–/56-FAM/CATGGAGGC/ZEN/CCTGTACTGGTCA/3IABkFX:-3′
	PRIMER 1	5′-GTCCGAGCTGAAGATCATGAG-3′
	PRIMER 2	5′-TCGCCATAGCAACAGTACTC-3′
DCSTAMP	PROBE	5′–/56-FAM/AGTGCAGAC/ZEN/AAGTCATGAGAGACCC/3IABkFQ/–3′
	PRIMER 1	5′-CGCTGAATAAGGAGGAAAGGA-3′
	PRIMER 2	5′-CGATGTGGCTGAGGAGTAGT-3′
FOS	PROBE	5′–/56-FAM/CAGACTCCT/ZEN/TCTCCAGCATGGGC/3IABkFQ/–3′
	PRIMER 1	5′-AGCCTCTCTTACTACCACTCAC-3′
	PRIMER 2	5″GGAATGAAGTTGGCACTGGA-3′
HPRT1	PROBE	5′–/56-FAM/TGGTGAAAA/ZEN/GGACCCCACGAAGT/3IABkFQ/–3′
	PRIMER 1	5′-AGATGGTCAAGGTCGCAAG-3′
	PRIMER 2	5′-GTATTCATTATAGTCAAGGGCATATCC-3′
MRC1	PROBE	5′–/56-FAM/TTCATGAGT/ZEN/AGGTTTAGCATCAATAATTTTTGGTCTTT/3IABkFQ/–3′
	PRIMER 1	5′-GGTTTTGGAGTAATATTCACTGTTCT-3′
	PRIMER 2	5′-TCCATCTTCCTTGTGTCAGC-3′
STAB1	PROBE	5′–/56-FAM:CTCCTGTGT/ZEN/GGACTGCCAAGCC/3IABkFQ/–3′
	PRIMER 1	5′-ACCATCCTGCCCATCCT-3′
	PRIMER 2	5′-TCCAGCTTCACACTGTTGG-3′
SUCNR1	PROBE	5′-/56FAM/ATCCCCAGC/ZEN/ATGTCGTAGTTGTTCAA/3IABkFQ/–3′
	PRIMER 1	5′-GGATCAAGTCTTCCAACAGAATG-3′
	PRIMER 2	5′-CCAGCCAGTTTTTGCAAGTTG-3′
TLR2	PROBE	5′–/56-FAM/CCAGTGCTT/ZEN/CAACCCACAACTACCA/3IABkFQ/–3′
	PRIMER 1	5′-CCATTGCTCTTTCACTGCTTTC-3′
	PRIMER 2	5′-ATGACCCCCAAGACCCA-3′

through a 70 μm nylon mesh (VWR, Cat # 732-2758). For DNA staining, cells were labeled with 10 μg/ml Hoechst 33342 (Sigma-Aldrich, Cat # B2261) in PBS supplemented with 1% FBS and 2 mM EDTA. Cells were then centrifuged and re-suspended in cold PBS. Phrodo red *S. aureus* bioparticles (Invitrogen, Cat # A10010) were added according to the manufacturer's instructions and incubated for 30 min at 37°C. Cells were re-centrifuged, fixed in 0.4% formaldehyde and analyzed with ImageStream (Amnis Corporation). Fluorescence was measured by ImageStream at 375 nm (Hoechst) and 581 nm (Phrodo red *S. aureus* bioparticles). Based on their size and Hoechst staining, cells were categorized as mononuclear and multinucleated cells. The mean fluorescence of Phrodo red *S. aureus* bioparticles was measured as a readout of phagocytic activity of the cells. Results were analyzed with Ideas v5 Software (Amnis Corporation).

Granuloma-like cluster formation

To generate granuloma-like cell clusters, cell differentiation was achieved by using PBMCs instead of isolated monocytes from blood donors. PBMCs (0.7×10^6) were incubated with conditioned media (MEM-REGA 3 [1×] + 10% FBS (Life Technologies)) containing M-CSF (20 ng/ml, PeproTech) and 10% FBS (Sigma-Aldrich, Cat # F7524) for 3 days. At day 3, cells were supplemented with either IFN-γ (25 ng/ml, PeproTech) or IL-4 (10 ng/ml, PeproTech) or RANKL (30 ng/ml, PeproTech). At day 7 of differentiation, cell culture media was removed and cells were washed twice in PBS prior to Giemsa staining and immunofluorescence analysis.

Extracellular flux analysis

Real-time measurements of oxygen consumption rate (OCR) and extracellular acidification rate (ECAR) were performed using a Seahorse XF96 Extracellular Flux Analyzer (Agilent Technologies). Donor-derived human osteoclasts were cultured for 7 days and sorted as mononuclear and multinuclear cells (see description above). Cells were then washed and 5×10^5 osteoclasts were seeded onto a XF96 plate containing Seahorse XF RPMI medium (Agilent Technologies). The cells were left for 1 h at 37°C after which the different metabolic drugs were injected (oligomycin 1 μM, FCCP 2 μM, rotenone/antimycin 1 μM, 2-DG 50 mM) during real-time measurements of OCR and ECAR, using the Seahorse XF Cell Mito Stress Test Kit (Agilent Technologies). Basal respiration was calculated as [the last measurement before addition of oligomycin—non-mitochondrial respiration (minimum rate measurement after Rot/AntA)]. Maximal respiration is shown as [the maximum rate measurement after addition of FCCP—non-mitochondrial respiration]. Estimated ATP production designates [the last measurement before addition of oligomycin—minimum rate after oligomycin]. Glycolysis refers to ECAR values before the addition of oligomycin.

Mitochondrial copy number

Total DNA was extracted from cell samples using the DNeasy Blood and Tissue kit (Qiagen, Valencia, CA, USA) with proteinase K and RNase treatment, according to the manufacturer's instructions. To quantify mtDNA copy number, real-time qPCR was performed using the ViiA 7 (Life Technologies) to measure gene expression against external standards for mitochondrial (tRNA Leu) and nuclear DNA (β2-microglobulin), as previously described (Amaral *et al*, 2007). Mitochondrial copy number was calculated using the formula (mt copy number = 2 × 2^(nuclear copy number − mitochondrial copy number)) for mononuclear and multinuclear macrophage population for each donor. The quantification of mitochondrial transcripts

© 2023 The Authors

in Fig 6G was then normalized by the mitochondria copy number (Fig 6F) to account for mitochondria numbers.

Bone resorption assay

The resorption activity of human osteoclasts and other MGCs was measured *in vitro* using Osteo Assay Surface 96-well plates (Corning) which are made of hydroxyapatite surfaces. MGCs were cultured until day 4 and incubated with cell dissociation buffer (Sigma; Catalogue # C5914). A total of 10^5 cells/well were seeded onto Osteo Assay Surface Plates. After 2 days of culture with the MGC-specific cytokines, the wells were rinsed twice in PBS and incubated with 10% bleach solution for 30 min at RT. The wells were then washed twice in PBS and allowed to dry at room temperature. Individual resorption pits were imaged by light microscopy. Images were inverted and processed using Photoshop to yield high-contrast images and show the resorbed areas in black against a white background. Binary images of each individual well were then subjected to automated analysis (ImageJ), using constant 'threshold' and 'minimum particle' levels, to determine the number and surface area of resorbed pits.

Giemsa and TRAP staining

Cells were fixed with methanol for 5 s and stained with Giemsa (1:10 dilution with MiliQ, VWR) for 17 min. After staining, cells were washed two times with water. For TRAP staining, cells were stained for tartrate-resistant acid phosphatase (TRAP), as previously described (De Klerck *et al*, 2004).

Statistical analysis

Data were analyzed using GraphPad Prism software, version v.9.0.1 (GraphPad Software). Data are presented as means + SD, indicated for each graph. The statistical test is indicated in the corresponding figure legend together with the number of biological replicates used. A paired *t*-test was used throughout except Fig EV3D (unpaired) and Fig 3E. Fusion index data (Fig 3E) were log transformed and significance was tested by one-way ANOVA followed by Sídàk's multiple comparison test. P value of < 0.05 was considered significant throughout. For microscopy images, representative images are shown.

Data availability

The RNA-sequencing datasets produced in this study are available in the following database: Gene Expression Omnibus GSE219114. https://www.ncbi.nlm.nih.gov/geo/query/acc.cgi?acc=GSE219114.

Expanded View for this article is available online.

Acknowledgements
We thank Carla Rios Luci from the NanoHealth and Optical Imaging Group for her support with the ImageStream experiments. Illustrative cartoons were designed with BioRender.com. This research paper was funded by the Medical Research Council UK (MR/N01121X/1) and Fonds Wetenschappelijk Onderzoek Vlaanderen (Research Foundation Flanders, FWO-Vlaanderen; G0C3420N and 1S75320N). We acknowledge additional funding support from Duke-NUS Medical School to J.B.

Author contributions
Kourosh Ahmadzadeh: Conceptualization; formal analysis; investigation; methodology; writing—original draft; writing—review and editing. **Marie Pereira:** Conceptualization; data curation; formal analysis; investigation; methodology; writing—original draft; writing—review and editing. **Margot Vanoppen:** Investigation; methodology. **Eline Bernaerts:** Investigation; methodology. **Jeong-Hun Ko:** Investigation; methodology. **Tania Mitera:** Investigation; methodology. **Christy Maksoudian:** Investigation; methodology. **Bella B Manshian:** Investigation; methodology. **Stefaan Soenen:** Investigation; methodology. **Carlos D Rose:** Writing—review and editing. **Patrick Matthys:** Conceptualization; supervision; funding acquisition; writing—review and editing. **Carine Wouters:** Conceptualization; supervision; funding acquisition; writing—review and editing. **Jacques Behmoaras:** Conceptualization; formal analysis; supervision; funding acquisition; investigation; writing—original draft; project administration; writing—review and editing.

Disclosure and competing interests statement
C.W. obtained unrestricted grants to KU Leuven from Novartis, Roche, GSK immuno-inflammation and Pfizer. The remaining authors state they have no competing interests or disclosures.

References

Adams DO (1976) The granulomatous inflammatory response. A review. *Am J Pathol* 84: 164–192

Ahmadzadeh K, Vanoppen M, Rose CD, Matthys P, Wouters CH (2022) Multinucleated Giant cells: current insights in phenotype, biological activities, and mechanism of formation. *Front Cell Dev Biol* 10: 873226

Amaral A, Ramalho-Santos J, St John JC (2007) The expression of polymerase gamma and mitochondrial transcription factor a and the regulation of mitochondrial DNA content in mature human sperm. *Hum Reprod* 22: 1585–1596

Anderson JM, Rodriguez A, Chang DT (2008) Foreign body reaction to biomaterials. *Semin Immunol* 20: 86–100

Aziz A, Soucie E, Sarrazin S, Sieweke MH (2009) MafB/c-Maf deficiency enables self-renewal of differentiated functional macrophages. *Science* 326: 867–871

Bar-Shavit Z (2007) The osteoclast: a multinucleated, hematopoietic-origin, bone-resorbing osteoimmune cell. *J Cell Biochem* 102: 1130–1139

Behmoaras J (2021) The versatile biochemistry of iron in macrophage effector functions. *FEBS J* 288: 6972–6989

Bowman EJ, Siebers A, Altendorf K (1988) Bafilomycins: a class of inhibitors of membrane ATPases from microorganisms, animal cells, and plant cells. *Proc Natl Acad Sci USA* 85: 7972–7976

Boyle WJ, Simonet WS, Lacey DL (2003) Osteoclast differentiation and activation. *Nature* 423: 337–342

Braune J, Lindhorst A, Froba J, Hobusch C, Kovacs P, Bluher M, Eilers J, Bechmann I, Gericke M (2021) Multinucleated Giant cells in adipose tissue are specialized in adipocyte degradation. *Diabetes* 70: 538–548

Brooks PJ, Glogauer M, McCulloch CA (2019) An overview of the derivation and function of multinucleated Giant cells and their role in pathologic processes. *Am J Pathol* 189: 1145–1158

Brukman NG, Uygur B, Podbilewicz B, Chernomordik LV (2019) How cells fuse. *J Cell Biol* 218: 1436–1451

 © 2023 The Authors

Chapoval AI, Ni J, Lau JS, Wilcox RA, Flies DB, Liu D, Dong H, Sica GL, Zhu G, Tamada K *et al* (2001) B7-H3: a costimulatory molecule for T cell activation and IFN-gamma production. *Nat Immunol* 2: 269 – 274

Chen Y, Jiang H, Xiong J, Shang J, Chen Z, Wu A, Wang H (2022) Insight into the molecular characteristics of Langhans Giant cell by combination of laser capture microdissection and RNA sequencing. *J Inflamm Res* 15: 621 – 634

Chung L, Maestas DR Jr, Lebid A, Mageau A, Rosson GD, Wu X, Wolf MT, Tam AJ, Vanderzee I, Wang X *et al* (2020) Interleukin 17 and senescent cells regulate the foreign body response to synthetic material implants in mice and humans. *Sci Transl Med* 12: eaax3799

Damsky W, Wang A, Kim DJ, Young BD, Singh K, Murphy MJ, Daccache J, Clark A, Ayasun R, Ryu C *et al* (2022) Inhibition of type 1 immunity with tofacitinib is associated with marked improvement in longstanding sarcoidosis. *Nat Commun* 13: 3140

Das BK, Wang L, Fujiwara T, Zhou J, Aykin-Burns N, Krager KJ, Lan R, Mackintosh SG, Edmondson R, Jennings ML *et al* (2022) Transferrin receptor 1-mediated iron uptake regulates bone mass in mice via osteoclast mitochondria and cytoskeleton. *Elife* 11: e73539

De Klerck B, Carpentier I, Lories RJ, Habraken Y, Piette J, Carmeliet G, Beyaert R, Billiau A, Matthys P (2004) Enhanced osteoclast development in collagen-induced arthritis in interferon-gamma receptor knock-out mice as related to increased splenic $CD11b^+$ myelopoiesis. *Arthritis Res Ther* 6: R220 – R231

Dondossola E, Holzapfel BM, Alexander S, Filippini S, Hutmacher DW, Friedl P (2016) Examination of the foreign body response to biomaterials by nonlinear intravital microscopy. *Nat Biomed Eng* 1: 0007

Drose S, Bindseil KU, Bowman EJ, Siebers A, Zeeck A, Altendorf K (1993) Inhibitory effect of modified bafilomycins and concanamycins on P- and V-type adenosinetriphosphatases. *Biochemistry* 32: 3902 – 3906

Duan X, Liu J, Zheng X, Wang Z, Zhang Y, Hao Y, Yang T, Deng H (2016) Deficiency of ATP6V1H causes bone loss by inhibiting bone resorption and bone formation through the TGF-beta1 pathway. *Theranostics* 6: 2183 – 2195

Friedman AD (2007) Transcriptional control of granulocyte and monocyte development. *Oncogene* 26: 6816 – 6828

Gast CE, Silk AD, Zarour L, Riegler L, Burkhart JG, Gustafson KT, Parappilly MS, Roh-Johnson M, Goodman JR, Olson B *et al* (2018) Cell fusion potentiates tumor heterogeneity and reveals circulating hybrid cells that correlate with stage and survival. *Sci Adv* 4: eaat7828

Geissmann F, Manz MG, Jung S, Sieweke MH, Merad M, Ley K (2010) Development of monocytes, macrophages, and dendritic cells. *Science* 327: 656 – 661

Gharun K, Senges J, Seidl M, Losslein A, Kolter J, Lohrmann F, Fliegauf M, Elgizouli M, Alber M, Vavra M *et al* (2017) Mycobacteria exploit nitric oxide-induced transformation of macrophages into permissive giant cells. *EMBO Rep* 18: 2144 – 2159

Gideon HP, Hughes TK, Tzouanas CN, Wadsworth MH 2nd, Tu AA, Gierahn TM, Peters JM, Hopkins FF, Wei JR, Kummerlowe C *et al* (2022) Multimodal profiling of lung granulomas in macaques reveals cellular correlates of tuberculosis control. *Immunity* 55: 827 – 846.10

Gordon S (2003) Alternative activation of macrophages. *Nat Rev Immunol* 3: 23 – 35

Gordon S, Cohn Z (1970) Macrophage-melanocyte heterokaryons. I. Preparation and properties. *J Exp Med* 131: 981 – 1003

Gordon S, Cohn Z (1971) Macrophage-melanocyte heterokaryons. II. The activation of macrophage DNA synthesis. Studies with inhibitors of RNA synthesis. *J Exp Med* 133: 321 – 338

Grigoriadis AE, Wang ZQ, Cecchini MG, Hofstetter W, Felix R, Fleisch HA, Wagner EF (1994) c-Fos: a key regulator of osteoclast-macrophage lineage determination and bone remodeling. *Science* 266: 443 – 448

ten Harkel B, Schoenmaker T, Picavet DI, Davison NL, de Vries TJ, Everts V (2015) The foreign body Giant cell cannot resorb bone, but dissolves hydroxyapatite like osteoclasts. *PLoS One* 10: e0139564

Heinz S, Benner C, Spann N, Bertolino E, Lin YC, Laslo P, Cheng JX, Murre C, Singh H, Glass CK (2010) Simple combinations of lineage-determining transcription factors prime cis-regulatory elements required for macrophage and B cell identities. *Mol Cell* 38: 576 – 589

Helming L, Gordon S (2007) Macrophage fusion induced by IL-4 alternative activation is a multistage process involving multiple target molecules. *Eur J Immunol* 37: 33 – 42

Helming L, Gordon S (2009) Molecular mediators of macrophage fusion. *Trends Cell Biol* 19: 514 – 522

Herrtwich L, Nanda I, Evangelou K, Nikolova T, Horn V, Sagar ED, Stefanowski J, Rogell L, Klein C *et al* (2016) DNA damage signaling instructs Polyploid macrophage fate in granulomas. *Cell* 167: 1264 – 1280.e18

Hughes CE, Coody TK, Jeong MY, Berg JA, Winge DR, Hughes AL (2020) Cysteine toxicity drives age-related mitochondrial decline by altering iron homeostasis. *Cell* 180: 296 – 310.e18

Hume DA, MacDonald KP (2012) Therapeutic applications of macrophage colony-stimulating factor-1 (CSF-1) and antagonists of CSF-1 receptor (CSF-1R) signaling. *Blood* 119: 1810 – 1820

Ioannou GN, Haigh WG, Thorning D, Savard C (2013) Hepatic cholesterol crystals and crown-like structures distinguish NASH from simple steatosis. *J Lipid Res* 54: 1326 – 1334

Ishii KA, Fumoto T, Iwai K, Takeshita S, Ito M, Shimohata N, Aburatani H, Taketani S, Lelliott CJ, Vidal-Puig A *et al* (2009) Coordination of PGC-1beta and iron uptake in mitochondrial biogenesis and osteoclast activation. *Nat Med* 15: 259 – 266

Itoh M, Kato H, Suganami T, Konuma K, Marumoto Y, Terai S, Sakugawa H, Kanai S, Hamaguchi M, Fukaishi T *et al* (2013) Hepatic crown-like structure: a unique histological feature in non-alcoholic steatohepatitis in mice and humans. *PLoS One* 8: e82163

Ivashkiv LB (2018) IFNgamma: signalling, epigenetics and roles in immunity, metabolism, disease and cancer immunotherapy. *Nat Rev Immunol* 18: 545 – 558

Jacome-Galarza CE, Percin GI, Muller JT, Mass E, Lazarov T, Eitler J, Rauner M, Yadav VK, Crozet L, Bohm M *et al* (2019) Developmental origin, functional maintenance and genetic rescue of osteoclasts. *Nature* 568: 541 – 545

Jin Z, Wei W, Yang M, Du Y, Wan Y (2014) Mitochondrial complex I activity suppresses inflammation and enhances bone resorption by shifting macrophage-osteoclast polarization. *Cell Metab* 20: 483 – 498

Joffin N, Gliniak CM, Funcke JB, Paschoal VA, Crewe C, Chen S, Gordillo R, Kusminski CM, Oh DY, Geldenhuys WJ *et al* (2022) Adipose tissue macrophages exert systemic metabolic control by manipulating local iron concentrations. *Nat Metab* 4: 1474 – 1494

Kanamori Y, Tanaka M, Itoh M, Ochi K, Ito A, Hidaka I, Sakaida I, Ogawa Y, Suganami T (2021) Iron-rich Kupffer cells exhibit phenotypic changes during the development of liver fibrosis in NASH. *iScience* 24: 102032

Kang H, Kerloc'h A, Rotival M, Xu X, Zhang Q, D'Souza Z, Kim M, Scholz JC, Ko JH, Srivastava PK *et al* (2014) Kcnn4 is a regulator of macrophage multinucleation in bone homeostasis and inflammatory disease. *Cell Rep* 8: 1210 – 1224

Kao WJ, McNally AK, Hiltner A, Anderson JM (1995) Role for interleukin-4 in foreign-body giant cell formation on a poly(etherurethane urea) *in vivo*. *J Biomed Mater Res* 29: 1267 – 1275

© 2023 The Authors

Lacombe J, Karsenty G, Ferron M (2013) Regulation of lysosome biogenesis and functions in osteoclasts. *Cell Cycle* 12: 2744–2752

Lavin Y, Winter D, Blecher-Gonen R, David E, Keren-Shaul H, Merad M, Jung S, Amit I (2014) Tissue-resident macrophage enhancer landscapes are shaped by the local microenvironment. *Cell* 159: 1312–1326

Lee SH, Rho J, Jeong D, Sul JY, Kim T, Kim N, Kang JS, Miyamoto T, Suda T, Lee SK *et al* (2006) v-ATPase V0 subunit d2-deficient mice exhibit impaired osteoclast fusion and increased bone formation. *Nat Med* 12: 1403–1409

Lemma S, Sboarina M, Porporato PE, Zini N, Sonveaux P, Di Pompo G, Baldini N, Avnet S (2016) Energy metabolism in osteoclast formation and activity. *Int J Biochem Cell Biol* 79: 168–180

Losslein AK, Lohrmann F, Scheuermann L, Gharun K, Neuber J, Kolter J, Forde AJ, Kleimeyer C, Poh YY, Mack M *et al* (2021) Monocyte progenitors give rise to multinucleated giant cells. *Nat Commun* 12: 2027

Ma F, Hughes TK, Teles RMB, Andrade PR, de Andrade Silva BJ, Plazyo O, Tsoi LC, Do T, Wadsworth MH 2nd, Oulee A *et al* (2021) The cellular architecture of the antimicrobial response network in human leprosy granulomas. *Nat Immunol* 22: 839–850

MacParland SA, Liu JC, Ma XZ, Innes BT, Bartczak AM, Gage BK, Manuel J, Khuu N, Echeverri J, Linares I *et al* (2018) Single cell RNA sequencing of human liver reveals distinct intrahepatic macrophage populations. *Nat Commun* 9: 4383

Madel MB, Ibanez L, Wakkach A, de Vries TJ, Teti A, Apparailly F, Blin-Wakkach C (2019) Immune function and diversity of osteoclasts in Normal and pathological conditions. *Front Immunol* 10: 1408

Madel MB, Ibanez L, Ciucci T, Halper J, Rouleau M, Boutin A, Hue C, Duroux-Richard I, Apparailly F, Garchon HJ *et al* (2020) Dissecting the phenotypic and functional heterogeneity of mouse inflammatory osteoclasts by the expression of Cx3cr1. *Elife* 9: e54493

McCaffrey EF, Donato M, Keren L, Chen Z, Delmastro A, Fitzpatrick MB, Gupta S, Greenwald NF, Baranski A, Graf W *et al* (2022) The immunoregulatory landscape of human tuberculosis granulomas. *Nat Immunol* 23: 318–329

McDonald MM, Khoo WH, Ng PY, Xiao Y, Zamerli J, Thatcher P, Kyaw W, Pathmanandavel K, Grootveld AK, Moran I *et al* (2021) Osteoclasts recycle via osteomorphs during RANKL-stimulated bone resorption. *Cell* 184: 1940

McNally AK, Anderson JM (1995) Interleukin-4 induces foreign body giant cells from human monocytes/macrophages. Differential lymphokine regulation of macrophage fusion leads to morphological variants of multinucleated giant cells. *Am J Pathol* 147: 1487–1499

McNally AK, Anderson JM (2005) Multinucleated giant cell formation exhibits features of phagocytosis with participation of the endoplasmic reticulum. *Exp Mol Pathol* 79: 126–135

McNally AK, Anderson JM (2011) Foreign body-type multinucleated giant cells induced by interleukin-4 express select lymphocyte co-stimulatory molecules and are phenotypically distinct from osteoclasts and dendritic cells. *Exp Mol Pathol* 91: 673–681

Milde R, Ritter J, Tennent GA, Loesch A, Martinez FO, Gordon S, Pepys MB, Verschoor A, Helming L (2015) Multinucleated Giant cells are specialized for complement-mediated phagocytosis and large target destruction. *Cell Rep* 13: 1937–1948

Mulder K, Patel AA, Kong WT, Piot C, Halitzki E, Dunsmore G, Khalilnezhad S, Irac SE, Dubuisson A, Chevrier M *et al* (2021) Cross-tissue single-cell landscape of human monocytes and macrophages in health and disease. *Immunity* 54: 1883–1900.e5

Novack DV, Teitelbaum SL (2008) The osteoclast: friend or foe? *Annu Rev Pathol* 3: 457–484

Oh Y, Park R, Kim SY, Park SH, Jo S, Kim TH, Ji JD (2021) B7-H3 regulates osteoclast differentiation via type I interferon-dependent IDO induction. *Cell Death Dis* 12: 971

Olona A, Mukhopadhyay S, Hateley C, Martinez FO, Gordon S, Behmoaras J (2021) Adipoclast: a multinucleated fat-eating macrophage. *BMC Biol* 19: 246

Pagan AJ, Ramakrishnan L (2018) The formation and function of granulomas. *Annu Rev Immunol* 36: 639–665

Papathanassiu AE, Ko JH, Imprialou M, Bagnati M, Srivastava PK, Vu HA, Cucchi D, McAdoo SP, Ananieva EA, Mauro C *et al* (2017) BCAT1 controls metabolic reprogramming in activated human macrophages and is associated with inflammatory diseases. *Nat Commun* 8: 16040

Pereira M, Petretto E, Gordon S, Bassett JHD, Williams GR, Behmoaras J (2018) Common signalling pathways in macrophage and osteoclast multinucleation. *J Cell Sci* 131: jcs216267

Pereira M, Chen TD, Buang N, Olona A, Ko JH, Prendecki M, Costa ASH, Nikitopoulou E, Tronci L, Pusey CD *et al* (2019) Acute iron deprivation reprograms human macrophage metabolism and reduces inflammation In vivo. *Cell Rep* 28: 498–511.e5

Pereira M, Ko JH, Logan J, Protheroe H, Kim KB, Tan ALM, Croucher PI, Park KS, Rotival M, Petretto E *et al* (2020) A trans-eQTL network regulates osteoclast multinucleation and bone mass. *Elife* 9: e55549

Petrany MJ, Swoboda CO, Sun C, Chetal K, Chen X, Weirauch MT, Salomonis N, Millay DP (2020) Single-nucleus RNA-seq identifies transcriptional heterogeneity in multinucleated skeletal myofibers. *Nat Commun* 11: 6374

Qin A, Cheng TS, Pavlos NJ, Lin Z, Dai KR, Zheng MH (2012) V-ATPases in osteoclasts: structure, function and potential inhibitors of bone resorption. *Int J Biochem Cell Biol* 44: 1422–1435

Ramstein J, Broos CE, Simpson LJ, Ansel KM, Sun SA, Ho ME, Woodruff PG, Bhakta NR, Christian L, Nguyen CP *et al* (2016) IFN-gamma-producing T-helper 17.1 cells are increased in sarcoidosis and are more prevalent than T-helper type 1 cells. *Am J Respir Crit Care Med* 193: 1281–1291

Roodman GD (1996) Paget's disease and osteoclast biology. *Bone* 19: 209–212

Rotival M, Ko JH, Srivastava PK, Kerloc'h A, Montoya A, Mauro C, Faull P, Cutillas PR, Petretto E, Behmoaras J (2015) Integrating phosphoproteome and transcriptome reveals new determinants of macrophage multinucleation. *Mol Cell Proteomics* 14: 484–498

Roy S, Schmeier S, Kaczkowski B, Arner E, Alam T, Ozturk M, Tamgue O, Parihar SP, Kawaji H, Itoh M *et al* (2018) Transcriptional landscape of mycobacterium tuberculosis infection in macrophages. *Sci Rep* 8: 6758

Sakai H, Okafuji I, Nishikomori R, Abe J, Izawa K, Kambe N, Yasumi T, Nakahata T, Heike T (2012) The CD40-CD40L axis and IFN-gamma play critical roles in Langhans giant cell formation. *Int Immunol* 24: 5–15

Saunders BM, Britton WJ (2007) Life and death in the granuloma: immunopathology of tuberculosis. *Immunol Cell Biol* 85: 103–111

Sheikh Z, Brooks PJ, Barzilay O, Fine N, Glogauer M (2015) Macrophages, foreign body Giant cells and their response to implantable biomaterials. *Materials (Basel)* 8: 5671–5701

Sommerfeld SD, Cherry C, Schwab RM, Chung L, Maestas DR Jr, Laffont P, Stein JE, Tam A, Ganguly S, Housseau F *et al* (2019) Interleukin-36gamma-producing macrophages drive IL-17-mediated fibrosis. *Sci Immunol* 4: eaax4783

Stanley ER, Chitu V (2014) CSF-1 receptor signaling in myeloid cells. *Cold Spring Harb Perspect Biol* 6: a021857

Sutton JS, Weiss L (1966) Transformation of monocytes in tissue culture into macrophages, epithelioid cells, and multinucleated giant cells. An electron microscope study. *J Cell Biol* 28: 303–332

 © 2023 The Authors

Takayanagi H (2021) RANKL as the master regulator of osteoclast differentiation. *J Bone Miner Metab* 39: 13 – 18

Takegahara N, Kim H, Mizuno H, Sakaue-Sawano A, Miyawaki A, Tomura M, Kanagawa O, Ishii M, Choi Y (2016) Involvement of receptor activator of nuclear factor-kappaB ligand (RANKL)-induced incomplete cytokinesis in the Polyploidization of osteoclasts. *J Biol Chem* 291: 3439 – 3454

Takegahara N, Kim H, Choi Y (2022) RANKL biology. *Bone* 159: 116353

Taylor PR, Martinez-Pomares L, Stacey M, Lin HH, Brown GD, Gordon S (2005) Macrophage receptors and immune recognition. *Annu Rev Immunol* 23: 901 – 944

Teitelbaum SL (2011) The osteoclast and its unique cytoskeleton. *Ann N Y Acad Sci* 1240: 14 – 17

Tsukasaki M, Huynh NC, Okamoto K, Muro R, Terashima A, Kurikawa Y, Komatsu N, Pluemsakunthai W, Nitta T, Abe T *et al* (2020) Stepwise cell fate decision pathways during osteoclastogenesis at single-cell resolution. *Nat Metab* 2: 1382 – 1390

Van Hove H, Martens L, Scheyltjens I, De Vlaminck K, Pombo Antunes AR, De Prijck S, Vandamme N, De Schepper S, Van Isterdael G, Scott CL *et al* (2019) A single-cell atlas of mouse brain macrophages reveals unique transcriptional identities shaped by ontogeny and tissue environment. *Nat Neurosci* 22: 1021 – 1035

Vignery A (2000) Osteoclasts and giant cells: macrophage-macrophage fusion mechanism. *Int J Exp Pathol* 81: 291 – 304

Walsh MC, Choi Y (2014) Biology of the RANKL-RANK-OPG system in immunity, bone, and beyond. *Front Immunol* 5: 511

Weber RA, Yen FS, Nicholson SPV, Alwaseem H, Bayraktar EC, Alam M, Timson RC, La K, Abu-Remaileh M, Molina H *et al* (2020) Maintaining iron homeostasis is the key role of lysosomal acidity for cell proliferation. *Mol Cell* 77: 645 – 655.e7

Weinberg JB, Hobbs MM, Misukonis MA (1984) Recombinant human gamma-interferon induces human monocyte polykaryon formation. *Proc Natl Acad Sci USA* 81: 4554 – 4557

Wouters CH, Maes A, Foley KP, Bertin J, Rose CD (2014) Blau syndrome, the prototypic auto-inflammatory granulomatous disease. *Pediatr Rheumatol Online J* 12: 33

Yagi M, Miyamoto T, Sawatani Y, Iwamoto K, Hosogane N, Fujita N, Morita K, Ninomiya K, Suzuki T, Miyamoto K *et al* (2005) DC-STAMP is essential for cell-cell fusion in osteoclasts and foreign body giant cells. *J Exp Med* 202: 345 – 351

Yagi M, Miyamoto T, Toyama Y, Suda T (2006) Role of DC-STAMP in cellular fusion of osteoclasts and macrophage giant cells. *J Bone Miner Metab* 24: 355 – 358

Yambire KF, Rostosky C, Watanabe T, Pacheu-Grau D, Torres-Odio S, Sanchez-Guerrero A, Senderovich O, Meyron-Holtz EG, Milosevic I, Frahm J *et al* (2019) Impaired lysosomal acidification triggers iron deficiency and inflammation *in vivo*. *Elife* 8: e51031

License: This is an open access article under the terms of the Creative Commons Attribution License, which permits use, distribution and reproduction in any medium, provided the original work is properly cited.

Expanded View Figures

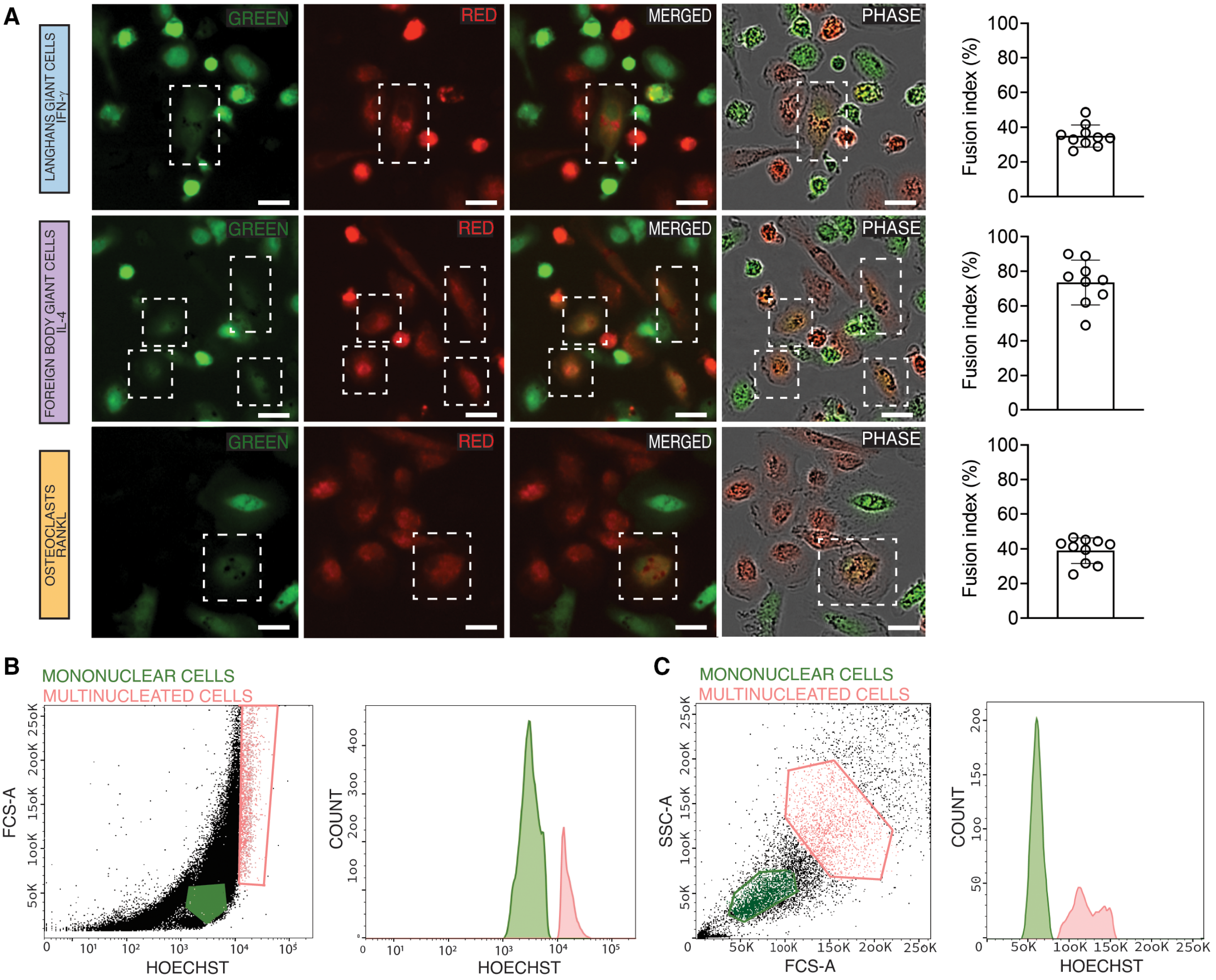

Figure EV1. Multinucleated giant cells are generated through cell–cell fusion.

A Isolated monocytes were labeled with either green or red dyes and stimulated with IFN-γ (LGCs), IL-4 (FBGC), or RANKL (osteoclasts) for giant cell formation. Cell–cell fusion was monitored with live cell imaging system (Incucyte). Orange dye-labeled giant cells (fusion between red and green) are shown within dotted white boxes. The fusion index is calculated for each cell type (right panel). The data are representative of three biological replicates (donors) and 3–4 technical replicates per donor.

B FACS sorting strategy for mononuclear and multinucleated (> 2 nuclei) LGCs and FBGCs based on size and Hoechst (DNA content as a readout of multinucleation).

C FACS sorting strategy for mononuclear and multinucleated (> 2 nuclei) osteoclasts based on size and Hoechst (DNA content as a readout of multinucleation).

Data information: Scale bar, 200 μm (A).

 © 2023 The Authors

Figure EV2. Fusion and multinucleation maintain lineage-specific pathways and cause down-regulation of a shared macrophage gene signature.

A Venn diagram showing the trancriptomic comparison between genes upregulated in mononucleated IFN-γ vs. IL-4 stimulated cells (designated as UP [IFN-γ_{mono} vs. IL-4_{mono}]) and in multinucleated LGCs vs. FBGCs (designated as *UP* [IFN-γ_{multi} vs. IL-4_{multi}]). The most significant group-specific pathways (BioPlanet 2019) are shown with arrows. The right panel shows the 5 most significant pathways for commonly upregulated 1,286 transcripts. Relevant pathways to IFN- γ are shown in red.

B Venn diagram showing the trancriptomic comparison between genes upregulated in mononucleated IL-4 vs. IFN-γ stimulated cells (designated as *UP* [IL-4_{mono} vs. IFN-γ_{mono}]) and in multinucleated FBGCs vs. LGCs (designated as *UP* [IL-4_{multi} vs. IFN-γ_{multi}]). The most significant group-specific pathways are shown with arrows whereas. The right panel shows the 5 most significant pathways for commonly upregulated 1,027 transcripts. Relevant pathways to IL-4 are shown in red. Data information: $n = 6$ donors (LGCs), $n = 7$ donors (FBGCs).

C *MRC1*, *CSF1R*, *TLR2*, *STAB1 and SUCNR1* relative expression measured by qRT–PCR in LGCs (upper), FBGCs (middle) and osteoclasts (lower), in sorted mononuclear and multinucleated cells; at least $n = 6$ donors. Error bars are mean ± SD; significance tested by paired t-test; *$P < 0.05$; **$P < 0.01$; ***$P < 0.001$; ****$P < 0.0001$.

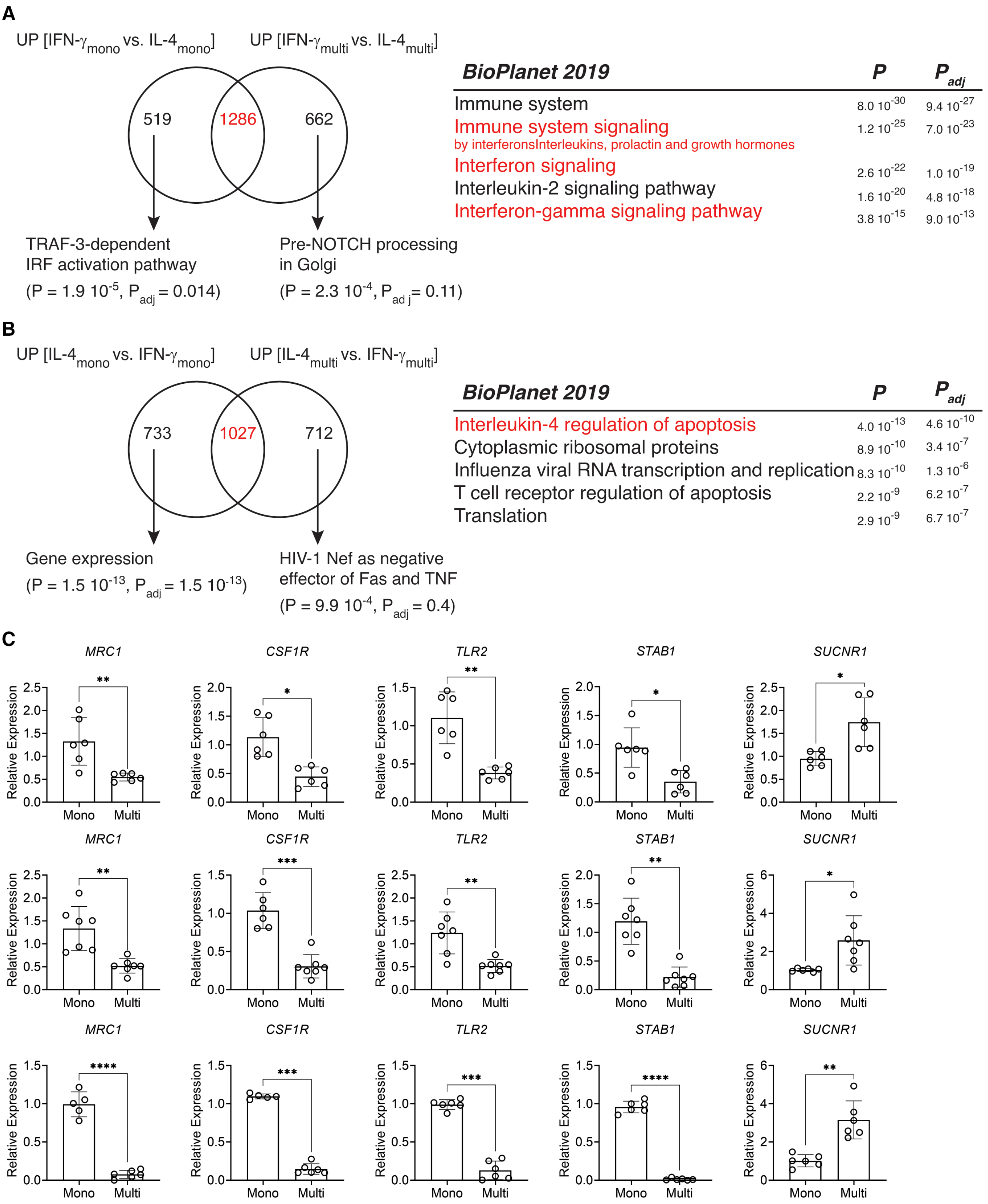

Figure EV2.

 © 2023 The Authors

Figure EV3. Fusion and multinucleation causes the downregulation of a shared macrophage gene signature between LGCs, FBGCs and osteoclasts.

A Protein–protein interaction (PPI) network of 191 commonly downregulated genes in LGCs, FBGCs and osteoclasts illustrated by STRING (high confidence score = 0.9, only connected nodes are shown).

B ImageStream gating for mononuclear and multinucleated cells shown in LGCs. The gating strategy for FBGCs and osteoclasts is similar.

C ImageStream showing bright field, nuclei staining (Hoechst), and MRC1 (red) staining in mononuclear and multinucleated LGCs, FBGCs and osteoclasts.

D MRC1 and CSF1R immunofluorescence quantification in mononuclear and multinucleated LGCs (upper), FBGCs (middle) and osteoclasts (lower); $n = 2$ donors (biological replicates), $n > 48$ technical replicates per cell type, condition (mono or multi) and surface marker (MRC1 or CSF1R).

E DC-STAMP expression following its knockdown in LGCs (left) and FBGCs (right). si-Ctrl, scrambled siRNA; si-DC-STAMP, DC-STAMP siRNA; $n = 7$ donors.

F Fusion index following DC-STAMP knockdown in human LGCs (upper panel) and FBGCs (lower panel); $n = 4$ donors. Fusion was measured in cells stained with Giemsa in both conditions (right panel).

Data information: Error bars are mean ± SD; significance tested by unpaired (D) and paired (E, F) *t*-test; $^{**}P < 0.01$; $^{****}P < 0.0001$; scale bar, 20 μm (C), 100 μm (F).

© 2023 The Authors

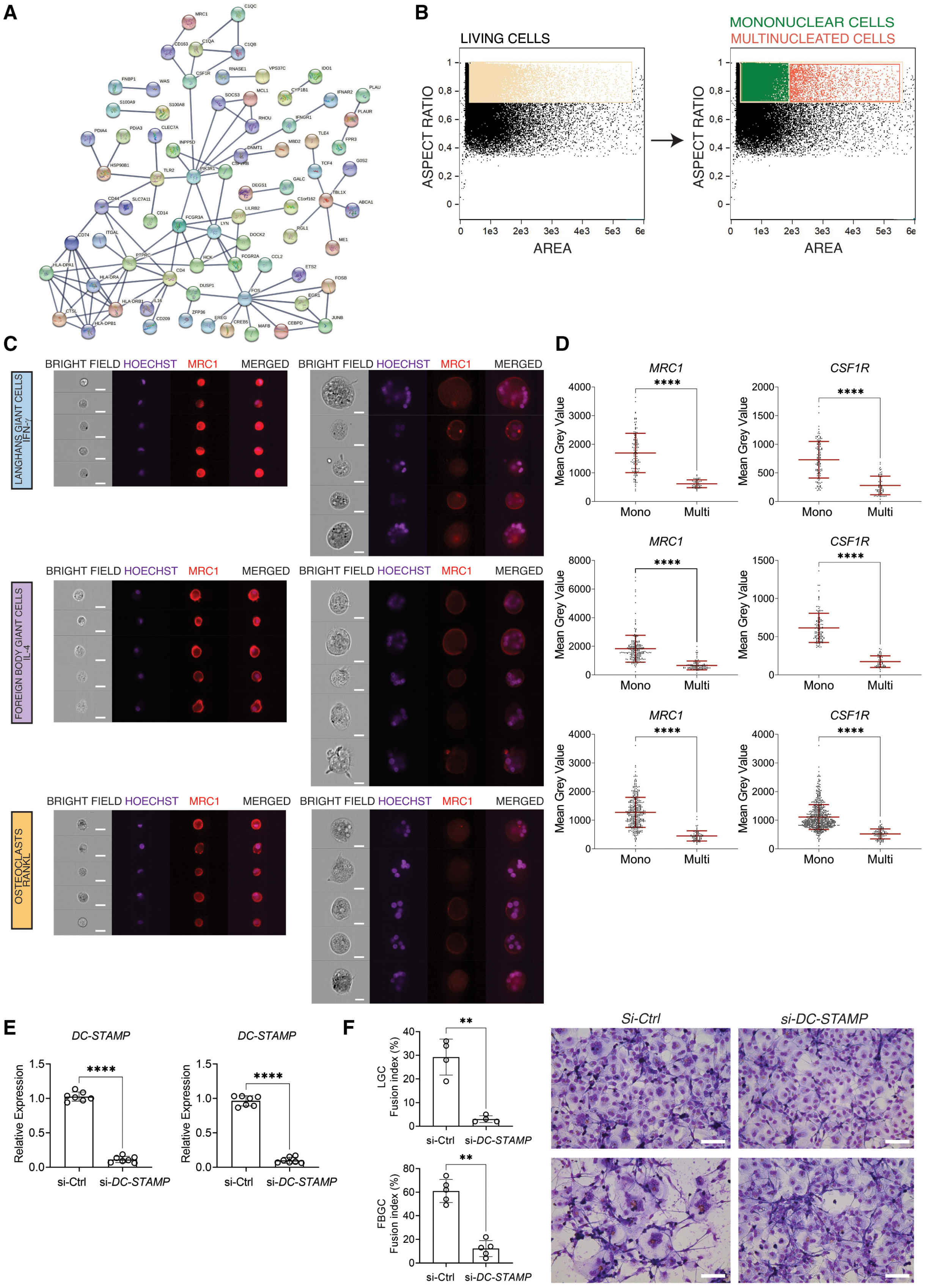

Figure EV3.

 © 2023 The Authors

Figure EV4. Multinucleation confers cluster-forming properties to LGCs *in vitro*.

A CD3 immunofluorescence in PBMC-derived FBGCs (upper panel) and osteoclasts (lower panel). Hoechst (gray) and phalloidin (red) staining show the nuclei and cytoskeleton, respectively.

B LGCs show increased membrane expression of B7-H3. ImageStream showing bright field, nuclei staining (Hoechst), and B7-H3 (red) staining in mononuclear and multinucleated LGCs, FBGCs and osteoclasts.

Data information: Scale bar, 20 μm.

© 2023 The Authors

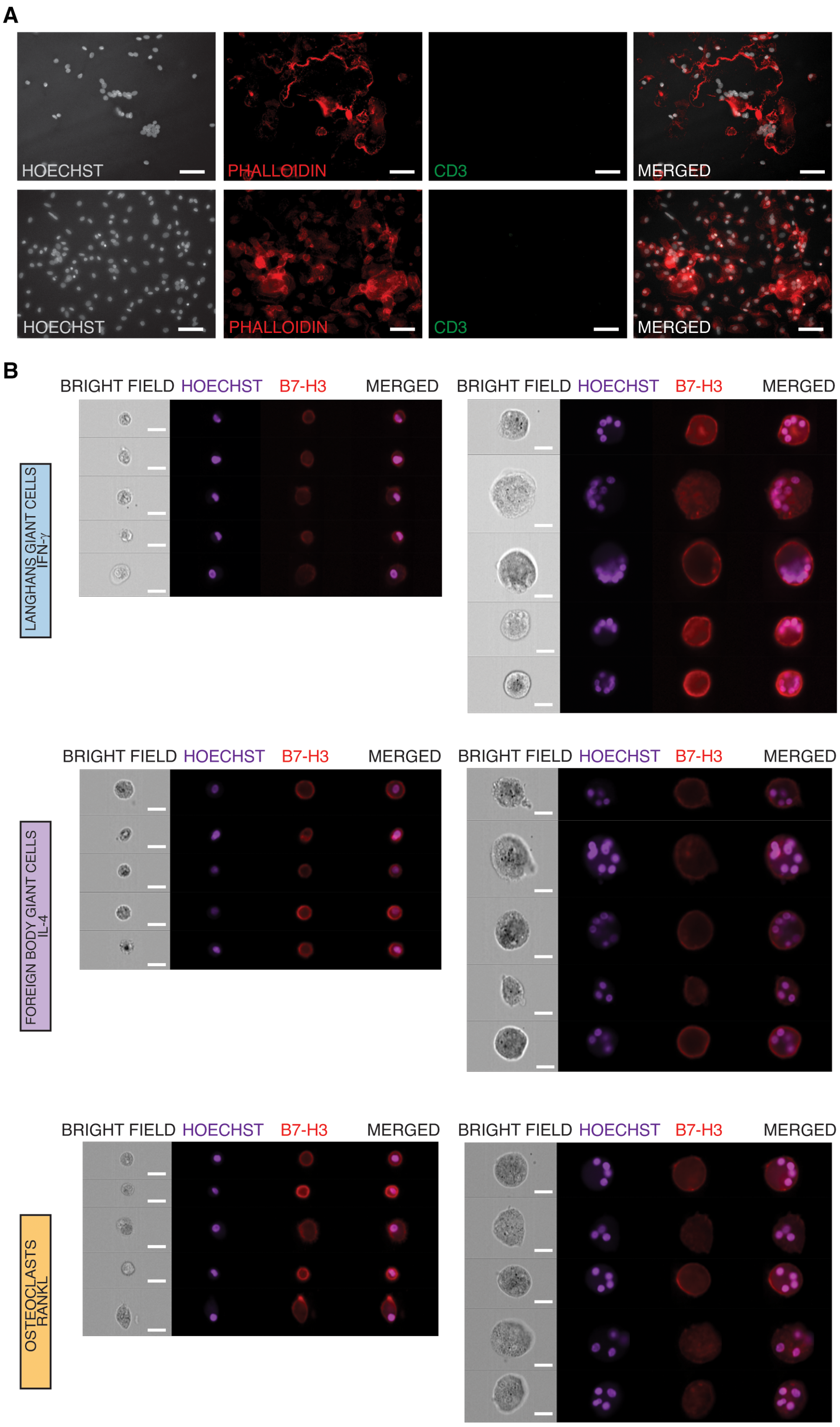

Figure EV4.

 © 2023 The Authors

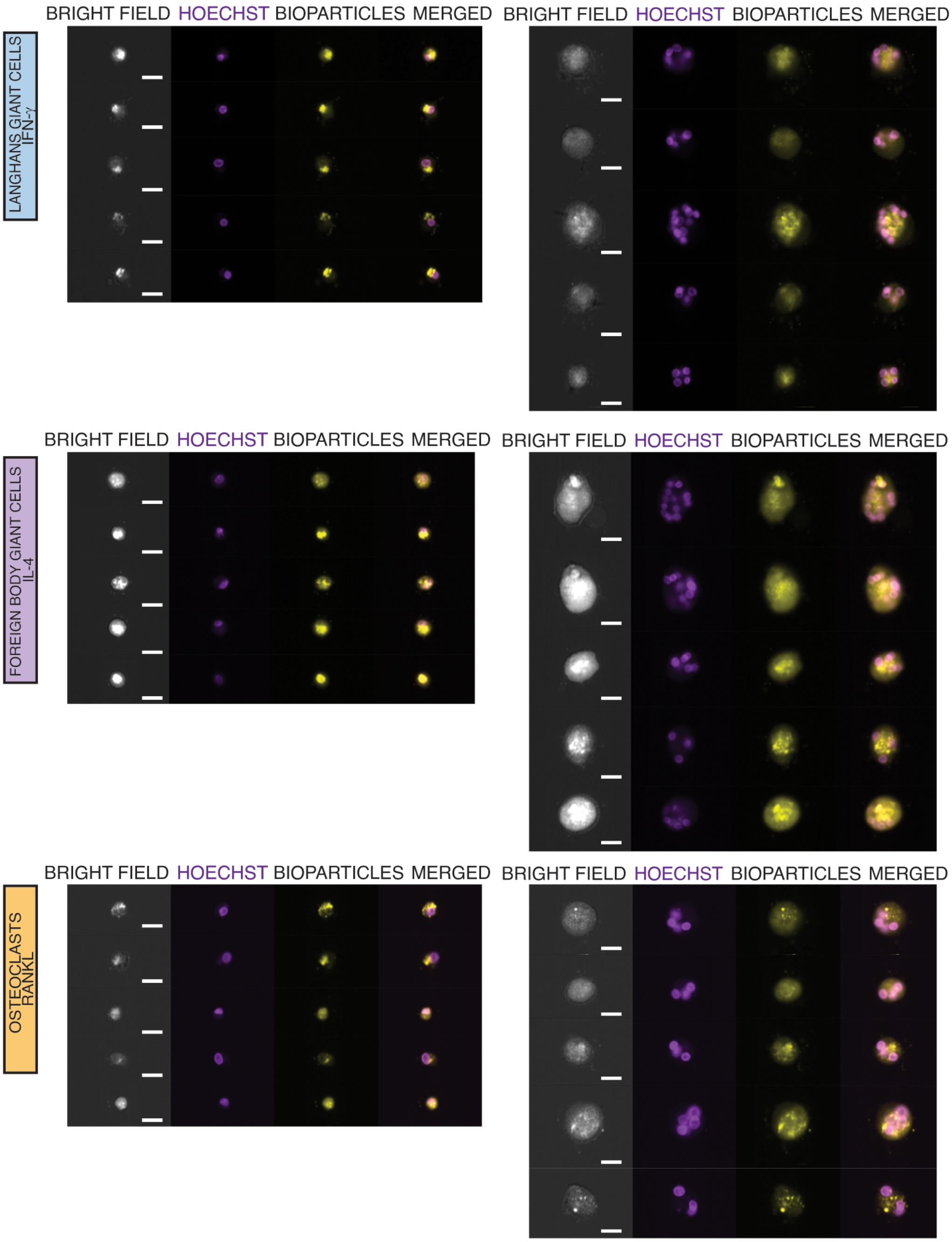

Figure EV5. Multinucleation causes enhanced phagocytosis in FBGCs.

FBGCs show a distinctively enhanced phagocytic capacity. ImageStream showing bright field, nuclei staining (Hoechst), and *S. aureus*-coated bioparticles (yellow) staining in mononuclear and multinucleated LGCs, FBGCs and osteoclasts. Scale bar, 20 μm.

© 2023 The Authors
