# Supplementary figures and images for: Multinucleation resets human macrophages for specialized functions at the expense of their identity
Source: EMBO Rep. 2023 Jan 4;24(3):e56310. doi: 10.15252/embr.202256310 (PMC9986822; doi:10.15252/embr.202256310)

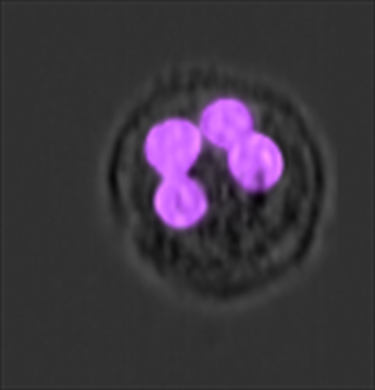

Supplement: Supplementary file 10 — Source Data for Figure 1 [file EMBR-24-e56310-s003.zip › Figure 1B/FBGC/FBGC_multi.png]

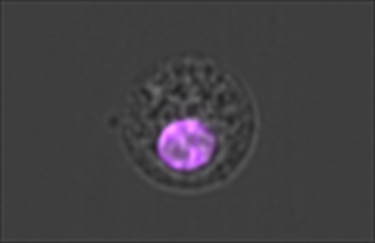

Supplement: Supplementary file 10 — Source Data for Figure 1 [file EMBR-24-e56310-s003.zip › Figure 1B/FBGC/IL-4_mono.png]

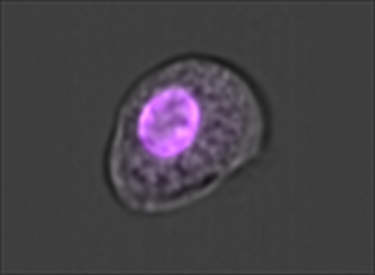

Supplement: Supplementary file 10 — Source Data for Figure 1 [file EMBR-24-e56310-s003.zip › Figure 1B/LGC/IFNg_mono.png]

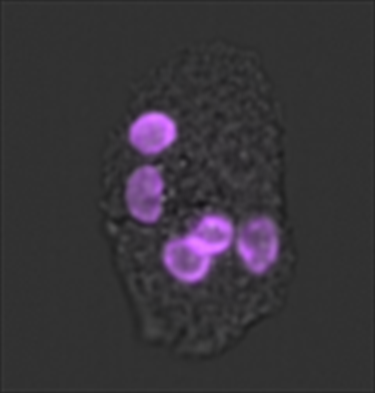

Supplement: Supplementary file 10 — Source Data for Figure 1 [file EMBR-24-e56310-s003.zip › Figure 1B/LGC/LGC_multi.png]

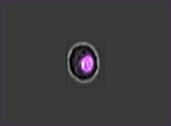

Supplement: Supplementary file 10 — Source Data for Figure 1 [file EMBR-24-e56310-s003.zip › Figure 1B/Osteoclast/Osteo_mono.png]

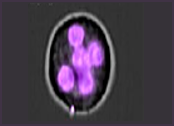

Supplement: Supplementary file 10 — Source Data for Figure 1 [file EMBR-24-e56310-s003.zip › Figure 1B/Osteoclast/Osteo_multi.png]

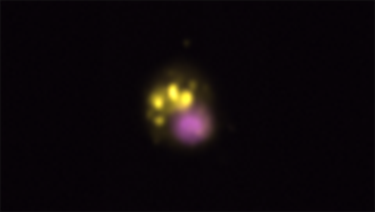

Supplement: Supplementary file 14 — Source Data for Figure 5 [file EMBR-24-e56310-s011.zip › Figure 5C/FBGC/IL4_mono.png]

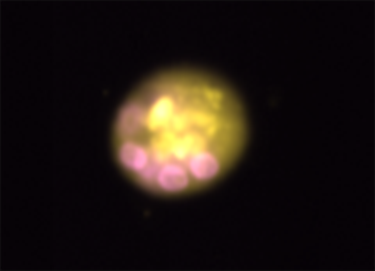

Supplement: Supplementary file 14 — Source Data for Figure 5 [file EMBR-24-e56310-s011.zip › Figure 5C/FBGC/IL4_multi.png]

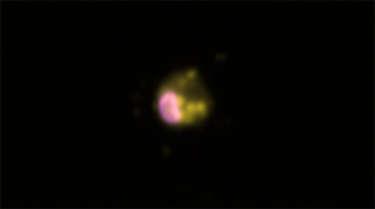

Supplement: Supplementary file 14 — Source Data for Figure 5 [file EMBR-24-e56310-s011.zip › Figure 5C/LGC/IFNg_mono.png]

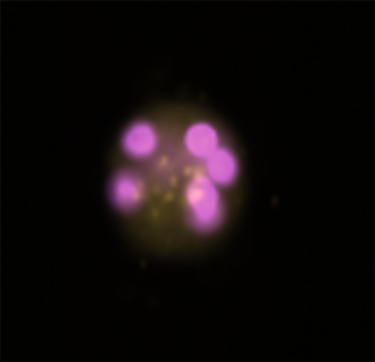

Supplement: Supplementary file 14 — Source Data for Figure 5 [file EMBR-24-e56310-s011.zip › Figure 5C/LGC/IFNg_multi.png]

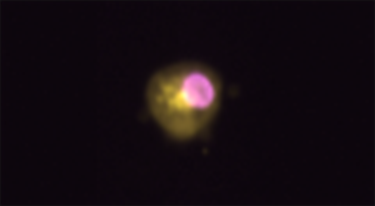

Supplement: Supplementary file 14 — Source Data for Figure 5 [file EMBR-24-e56310-s011.zip › Figure 5C/Osteoclast/Osteoclast_mono.png]

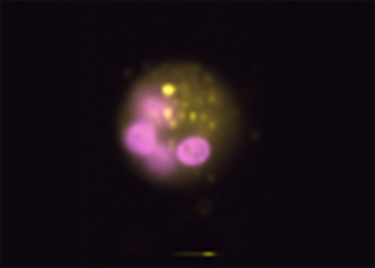

Supplement: Supplementary file 14 — Source Data for Figure 5 [file EMBR-24-e56310-s011.zip › Figure 5C/Osteoclast/Osteoclast_multi.png]
