# Supplementary material for: Multinucleation resets human macrophages for specialized functions at the expense of their identity
Source: EMBO Rep. 2023 Jan 4;24(3):e56310. doi: 10.15252/embr.202256310 (PMC9986822; doi:10.15252/embr.202256310)
Supplement: Supplementary file 16 [file EMBR-24-e56310-s007.pdf]

EMBO reports

Multinucleation resets human macrophages for specialized functions at the expense of their identity

Kourosh Ahmadzadeh, Marie Pereira, Margot Vanoppen, Eline Bernaerts, Jeong-Hun Ko, Tania Mitera, Christy Maksoudian, Bella Manshian, Stefaan Soenen, Carlos Rose, Patrick Matthys, Carine Wouters, and Jacques Behmoaras
DOI: 10.15252/embr.202256310

Corresponding author(s): Jacques Behmoaras (jacquesb@duke-nus.edu.sg) , Patrick Matthys (patrick.matthys@kuleuven.be), Carine Wouters (carine.wouters@uzleuven.be)

Review Timeline:

Transfer from Review Commons:	17th Oct 22
Editorial Decision:	7th Nov 22
Revision Received:	5th Dec 22
Editorial Decision:	7th Dec 22
Revision Received:	8th Dec 22
Accepted:	9th Dec 22

Editor: Achim Breiling

Review COMMONS

Transaction Report: This manuscript was transferred to EMBO Reports following peer review at Review Commons.

(Note: With the exception of the correction of typographical or spelling errors that could be a source of ambiguity, letters and reports are not edited. Depending on transfer agreements, referee reports obtained elsewhere may or may not be included in this compilation. Referee reports are anonymous unless the Referee chooses to sign their reports.)

Review #1

1. Evidence, reproducibility and clarity:

Evidence, reproducibility and clarity (Required)

Summary:

The authors compared the various multinucleated cells, osteoclasts, LCG and FBGC. Overall, the manuscript shows rigor in the analyses, and also very interesting approaches for retrieving mononuclear cells, for instance using DC-STAMP siRNA. This work adds very much to understanding the biological differences, as summarized in figure 6h. A lot of work in osteoclast field with for instance qPCR is hampered because, inevitably, a mix of mononuclear and multinucleated cells is always measured. Here, a solid attempt to separate those mixes with cell sorting and subsequent analysis on the mononuclear and multinucleated isolates, really adds. Choice of figures is good, also the extra info of the supplementary figures is relevant and makes it easy to read.

Major and minor concerns:

For osteoclasts, various markers exist for their biological characterization, for instance the ability to resorb bone. What, apart from the arrangement and number of nuclei, were the biological parameters that confirmed that the cells made by addition of IFN or IL-4 were LCG and FBGC?
In fig 2c: did the authors perform stainings with isotype control antibodies? In my experience, quite often, antibodies stain mononuclear cells much intenser, since the cytoplasm is much more condense, less spread over a large area.
Resorption assay in 6 is not clear. It is weird that osteoclasts apparently display so limited resorption? Also the traces are not typical for osteoclasts. Please explain.
Provide a better image Supplementary 2A, even at 250% the lettering is vague. What do the colours in 2A mean?

CROSS-CONSULTATION COMMENTS

I have read the comments of the other two reviewers, and together. I absolutely agree with their additions, Indeed, supplementary tables are lacking, as well as there could be a bit more emphasis on the fact that it is all in vitro work. Together, I think the three of us are complementary in our comments, with good overlap as well. Any effort to stain for instance pathology material with the markers that have been found, would be great, especially for the LGC and the FBGC, that are much less studied in the field of MNGs. Having said that ,I can also live without this addition, but then it could be highlighted in the discussion that these are the future avenues that should be considered. Collaborate with Pathology!

2. Significance:

Significance (Required)

This manuscript is particularly interesting to those who are interested in the BIOLOGY of MNCs. In essence, three types of MNCs were cultured and compared, with each of them a specific function.

I am an osteoclast expert (76 publications), and have two publications on FBGCs

3. How much time do you estimate the authors will need to complete the suggested revisions:

Estimated time to Complete Revisions (Required)

(Decision Recommendation)

Less than 1 month

4. Review Commons values the work of reviewers and encourages them to get credit for their work. Select 'Yes' below to register your reviewing activity at [Publons]; note that the content of your review will not be visible on Publons.

Reviewer Publons

Yes

Review #2

1. Evidence, reproducibility and clarity:

Evidence, reproducibility and clarity (Required)

Summary:

In this manuscript, the authors performed a comparative transcriptome analysis of mononuclear and multinuclear human osteoclasts, LGCs and FBGCs. They found that multinucleation triggers a significant downregulation of macrophage identity in all three types of MGCs. Furthermore, RNA-seq data and in-vitro functional analysis of multinucleated cells showed that macrophage cell-cell fusion and multinucleation enhance phagocytosis and contribute to lysosome-dependent

intracellular iron homeostasis. Furthermore, multinucleation of osteoclasts promoted mitochondrial activity and oxidative phosphorylation, resulting in maximal respiration. This unique and interesting study addresses the fundamental question of how cell-cell fusion and multinucleation contribute to cellular activity and biological homeostasis.

Major comments:

1. The authors generated mature multinucleated cells by stimulating human PBMC-derived macrophages with either IFN-g, IL-4, or RANKL. However, no quantitative data have been presented to determine how effectively IL-4, IFN-g, and RANKL can induce multinucleated giant cells from mononuclear macrophages. Quantitative data showing induction efficiency would provide a more detailed picture of the overall experiment.

2. The authors mentioned, "The distinct morphological appearance of these three types of MGCs (Figure 1B) suggested cell type-specific functional properties and shared mechanisms underlying macrophage multinucleation". However, there is no discussion or data showing how the nuclear arrangement and intracellular location affect the biological function of multinucleated cells.

3. Based on the results of DC-stamp knockdown experiments, the authors concluded that cell-cell fusion and multinucleation suppress the mononuclear phagocytic gene signature. However, to strengthen this hypothesis, it would be necessary to provide at least data showing that DC-stamp knockdown reduces the number of multinucleated cells.

4. In Figure4, the authors showed that transcripts in LGCs were enriched for antigen presentation and adaptive immune system pathways. In addition, multinucleation of LGCs increased the surface expression of B7-H3 (CD276) and colocalized with CD3+ cells, suggesting that LGC multinucleation potentiates T cell activation. However, the authors did not present enough data to demonstrate the antigen-presenting ability of LGCs or their specific T cell activating capacity.

5. Figure 6 clearly shows that mature multinucleated osteoclasts exhibit increased ATP production and maximal respiration. However, the glycolytic pathway did not differ between mononuclear and multinuclear osteoclasts. No explanation for this observation has been provided. It is easy to understand that osteoclasts acquire ATP through aerobic respiration during multinucleation. But how NADPH, which is essential for its redox reaction, is produced? Is it by acquiring αKG from the glutamine pathway?

Minor comments:

1. Supplementary tables 1-6 were not provided.

2. Figure 2D right panel, difficult to see DAPI+ nuclei.

2. Significance:

Significance (Required)

Although it is well known that multinucleation of cells constantly occurs, especially in osteoclasts, skeletal muscle, and trophoblasts of the placenta, the biological significance of multinucleation and the intracellular functions of multinucleation are not well understood. In this unique study, three types of multinucleated cells were generated from human peripheral blood to elucidate the genetic and functional differences between mononucleated and multinucleated cells. Furthermore, by demonstrating the possibility that the morphological peculiarity of multinucleation can regulate cell function, this paper provides clues to understanding the underlying biology of multinucleated cells and how they maintain cell function in homeostatic and pathological settings.

3. How much time do you estimate the authors will need to complete the suggested revisions:

Estimated time to Complete Revisions (Required)

(Decision Recommendation)

Between 1 and 3 months

4. Review Commons values the work of reviewers and encourages them to get credit for their work. Select 'Yes' below to register your reviewing activity at Publons; note that the content of your review will not be visible on Publons.

Reviewer Publons

Yes

Review #3

1. Evidence, reproducibility and clarity:

Evidence, reproducibility and clarity (Required)

Summary:

The manuscript of Ahmadzadeh and Pereira et al is an interesting study of the fusion process key to the formation of multinucleated giant cells (MGCs). Our current ability to discriminate between different types of MGCs is limited, and there are gaps in our understanding of the

molecular determinants of cell fusion. In this study, the authors isolated different MGC variants - osteoclasts, Langhans giant cells (LGCs) and foreign body giant cells (FBGCs) and identified common, as well as MGC-specific genes and pathways involved in the process of cell fusion. The approach of isolating and comparing different types of MGCs is novel, and the manuscript is well presented and written. However, due to the in vitro nature of the study, the physiological significance of the findings is unclear. I have further minor and major points for the authors to address, as detailed below.

Minor comments:

• The approach to isolate the different MGCs using FACS and imaging technique is highly novel. However the difference between MGC subtypes isolated isn't immediately apparent beyond the morphological comparisons. In my opinion some of the results of MGC-specific assays from Figures 4, 5 and 6 can be included in Figure 1, e.g. TRAP staining and hydroxyapatite resorption for osteoclasts, to provide evidence of purity and specificity of each MGC subtype early on in the manuscript. Classical or canonical genes associated with each MGC subtype can also be highlighted in the volcano plots in Figure 1C, e.g ACP5, CTSK, TNFRSF11A for osteoclasts.

• The overall decrease in phagocytic identity of all the MGCs, and the specific upregulation of phagocytic pathways in the FBGCs are conflicting. Are there subsets of phagocytic pathways that were down and upregulated during the formation of FBGCs?

• What are the identities of the mononuclear cells in each of the MGC experiment? They appeared to be quite heterogeneous based on the DEGs identified, beyond the common phagocyte signature. Can the authors comment on the difference between the mononuclear cells and whether this will affect the DEG analysis?

• The authors should also frame/discuss the findings in the context of diagnostic and therapeutic potentials to highlight the clinical significance of this study

Major comments:

• As mentioned before, the physiological significance of the findings is unclear. Some form of in vivo data is needed to support some of the key conclusions of the study, e.g validating some of the markers of the pathways identified (common and MGC subtype-specific), and the role of lysosome-mediated iron homeostasis in multinucleation. The authors can make use of the FACs and imaging approaches they developed to look at MGCs in relevant tissues.

2. Significance:

Significance (Required)

Significance:

• The approach of isolating and comparing different types of MGCs is novel, and the findings

certainly improved our understanding of the fusion processes of MGCs. However the physiological role of these processes in health and disease that involve MGCs is still unclear due to the lack of in vivo data. The findings were discussed in quite a bit of detail in the context of current literature, though clinical impact was not explored.

• My background is bone biology with a very keen interest in osteoclast biology so arguably my knowledge on other MGCs eg LGCs and FBGCs is limited.

3. How much time do you estimate the authors will need to complete the suggested revisions:

Estimated time to Complete Revisions (Required)

(Decision Recommendation)

Between 3 and 6 months

4. Review Commons values the work of reviewers and encourages them to get credit for their work. Select 'Yes' below to register your reviewing activity at Publons; note that the content of your review will not be visible on Publons.

Reviewer Publons

Yes

Full Revision

Review COMMONS

Manuscript number: #RC-2022-01638
Corresponding author(s): Jacques Behmoaras

[Please use this template only if the submitted manuscript should be considered by the affiliate journal as a full revision in response to the points raised by the reviewers.

If you wish to submit a preliminary revision with a revision plan, please use our "Revision Plan" template. ***It is important to use the appropriate template to clearly inform the editors of your intentions.****]*

1. General Statements [optional]

[Authors' reply]*. We thank all the reviewers for their very constructive comments. We note that our findings were unanimously received as novel and unique. The reviewers highlighted the rigor in data analysis (Reviewer 1) and the improvement of our understanding of the fusion process and the underlying biology (Reviewer 1, 2 and 3) – a characteristic of macrophages among all other immune cells. We have addressed all the points raised by the reviewers and provide a fully revised version of our manuscript.*

2. Point-by-point description of the revisions

Reviewer #1 (Evidence, reproducibility and clarity (Required)):

Summary: The authors compared the various multinucleated cells, osteoclasts, LCG and FBGC. Overall, the manuscript shows rigor in the analyses, and also very interesting approaches for retrieving mononuclear cells, for instance using DC-STAMP siRNA. This work adds very much to understanding the biological differences, as summarized in figure 6h. A lot of work in osteoclast field with for instance qPCR is hampered because, inevitably, a mix of mononuclear and multinucleated cells is always measured. Here, a solid attempt to separate those mixes with cell sorting and subsequent analysis on the mononuclear and multinucleated isolates, really adds. Choice of figures is good, also the extra info of the supplementary figures is relevant and makes it easy to read.

Major and minor concerns:

1. For osteoclasts, various markers exist for their biological characterization, for instance the ability to resorb bone. What, apart from the arrangement and number of nuclei, were the biological parameters that confirmed that the cells made by addition of IFN or IL-4 were LCG and FBGC?

[Authors' reply]. *In order to address this point, we focused on gene sets that characterize LCGs and FBGCs. By doing so, we aimed to identify (i) lineage dependent factors and (ii) markers of*

LGCs and FBGCs. (See new Supplementary Figure 1B and C, New Supplementary Table 1 and highlighted text in Results). As expected, and in line with the lineage-determining factors, the transcriptomics comparison between mononucleated/multinucleated IFN-γ and IL-4-differentiated macrophages showed predominance of IFN-γ and IL-4-related pathways, respectively (Supplementary Figure 1B and C and Supplementary Table 1). Among known LGC and FBGC markers, we confirmed up-regulation of CCL7 [1] and CD86 [2], respectively. As per the biological parameters, we indeed confirm that FBGCs show enhanced phagocytosis properties (Figure 5C) while LGCs can form granuloma-like clusters in vitro (Figure 4D and E). Altogether, we characterize LGCs and FBGCs with (i) polykaryon-specific nuclear arrangement, (ii) polykaryon-specific gene expression markers, (iii) previously shown and new phenotypic characteristics such as LGCs' unique ability to form in vitro clusters containing CD3+ cells.

2. In fig 2c: did the authors perform stainings with isotype control antibodies? In my experience, quite often, antibodies stain mononuclear cells much intenser, since the cytoplasm is much more condense, less spread over a large area.

[Authors' reply]. *According to the reviewer's suggestion, we provide isotype control staining for MRC1 in IFN-γ-stimulated mononucleated/multinucleated cells by ImageStream (left panel) and immunofluorescence in LGCs, FBGCs and osteoclasts (right panel). There was negligible staining with the isotype control antibody for MRC1 in both settings (see below).*

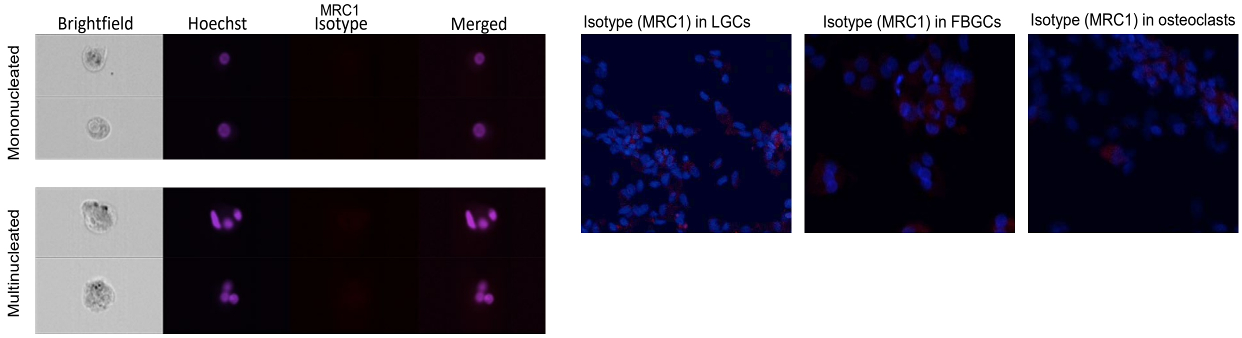

We did not observe a potential artefact of staining in multinucleated cells when compared to mononuclear cells. In fact, some markers of multinucleation such as B7-H3 is augmented in LGCs (Figure 4E).

3. Resorption assay in 6 is not clear. It is weird that osteoclasts apparently display so limited resorption? Also the traces are not typical for osteoclasts. Please explain.

[Authors' reply]. *Human osteoclasts are cultured for 2 days on hydroxyapatite-coated plates and the amount of resorption is dependent on the healthy donor the peripheral blood is derived from. In addition to genetic variability, the support (hydroxyapatite) is different from dentine, which is also widely used for measuring osteoclast resorptive activity. The visualization of the human osteoclast resorption is made by transparency (area not coated by hydroxyapatite due to its resorption) on image J.*

4. Provide a better image Supplementary 2A, even at 250% the lettering is vague. What do the colours in 2A mean?

[Authors' reply]. *According to the reviewer's suggestion, we now provide the Supplementary Figure 2A with better resolution. In STRING protein-protein interaction analysis, there is no particular meaning of the node color itself.*

CROSS-CONSULTATION COMMENTS

I have read the comments of the other two reviewers, and together. I absolutely agree with their additions, Indeed, supplementary tables are lacking, as well as there could be a bit more emphasis on the fact that it is all in vitro work. Together, I think the three of us are complementary in our comments, with good overlap as well. Any effort to stain for instance pathology material with the markers that have been found, would be great, especially for the LGC and the FBGC, that are much less studied in the field of MNGs. Having said that ,I can also live without this addition, but then it could be highlighted in the discussion that these are the future avenues that should be considered. Collaborate with Pathology!

[Authors' reply]. *We appreciate that the reviewer provides cross-consultation comments which we address in our revised manuscript. As such, we discuss future avenues regarding the translatability of these results to human pathology involving MGCs.*

Reviewer #1 (Significance (Required)):

This manuscript is particularly interesting to those who are interested in the BIOLOGY of MNCs. In essence, three types of MNCs were cultured and compared, with each of them a specific function.

I am an osteoclast expert (76 publications), and have two publications on FBGCs

[Authors' reply]. *We sincerely thank the reviewer for his/her pertinent comments, enthusiasm for our findings and for providing us an overall summary of our findings in view of all other reviewer comments.*

Reviewer #2 (Evidence, reproducibility and clarity (Required)):

Summary:

In this manuscript, the authors performed a comparative transcriptome analysis of mononuclear and multinuclear human osteoclasts, LGCs and FBGCs. They found that multinucleation triggers a significant downregulation of macrophage identity in all three types of MGCs. Furthermore, RNA-seq data and in-vitro functional analysis of multinucleated cells showed that macrophage cell-cell fusion and multinucleation enhance phagocytosis and contribute to lysosome-dependent intracellular iron homeostasis. Furthermore, multinucleation of osteoclasts promoted mitochondrial activity and oxidative phosphorylation, resulting in maximal respiration. This unique

and interesting study addresses the fundamental question of how cell-cell fusion and multinucleation contribute to cellular activity and biological homeostasis.

Major comments

#1 The authors generated mature multinucleated cells by stimulating human PBMC-derived macrophages with either IFN-g, IL-4, or RANKL. However, no quantitative data have been presented to determine how effectively IL-4, IFN-g, and RANKL can induce multinucleated giant cells from mononuclear macrophages. Quantitative data showing induction efficiency would provide a more detailed picture of the overall experiment.

[Authors' reply]. *According to the reviewer's suggestion, quantitative data showing the efficiency of these cytokines to induce multinucleation (i.e. fusion index) is now provided as part of the revised Supplementary Figure 1A (right panel).*

#2 The authors mentioned, "The distinct morphological appearance of these three types of MGCs (Figure 1B) suggested cell type-specific functional properties and shared mechanisms underlying macrophage multinucleation". However, there is no discussion or data showing how the nuclear arrangement and intracellular location affect the biological function of multinucleated cells.

[Authors' reply]. *This is good point and is now discussed in the revised manuscript (see highlighted text in revised manuscript and below).*

Whether MGC-specific nuclear arrangements and/or numbers are indicative of specialized function is currently unclear. Intracellular nuclei arrangement is likely to be important for the sealing zone formation in a polarized bone-resorbing osteoclast. Furthermore, whether distinct transcriptional activities are assigned to different nuclei of the MGC also remain to be tested. Recent elegant work performed in multinucleated skeletal myofibers suggest transcriptional heterogeneity among the different nuclei of the polykaryon [3].

#3 Based on the results of DC-stamp knockdown experiments, the authors concluded that cell-cell fusion and multinucleation suppress the mononuclear phagocytic gene signature. However, to strengthen this hypothesis, it would be necessary to provide at least data showing that DC-stamp knockdown reduces the number of multinucleated cells.

[Authors' reply]. *According to the reviewer's suggestion, we provide data showing that DCSTAMP knockdown reduces multinucleation in LGCs and FBGCs (see below and new Supplementary Figure 2F). For human osteoclasts, the data was included in our previously published paper ([4] and below).*

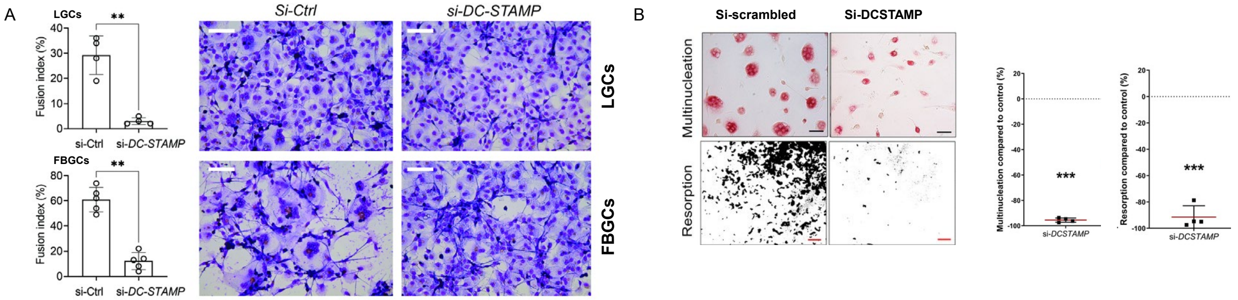

Figure. **A**. Fusion index following DC-STAMP knockdown in human LGCs (upper panel) and FBGCs (lower panel); n=4 donors; significance tested by paired t-test. Fusion was measured in cells stained with Giemsa. **B**. Human osteoclast multinucleation (upper panel, TRAP staining) and resorption (lower panel, hydroxyapatite) in si-Scrambled and si-DCSTAMP conditions. Quantification is shown on the right panel. Data presented in panel **B** are from Pereira et al., Elife 2020 [PMID:32553114].

#4 In Figure4, the authors showed that transcripts in LGCs were enriched for antigen presentation and adaptive immune system pathways. In addition, multinucleation of LGCs increased the surface expression of B7-H3 (CD276) and colocalized with CD3+ cells, suggesting that LGC multinucleation potentiates T cell activation. However, the authors did not present enough data to demonstrate the antigen-presenting ability of LGCs or their specific T cell activating capacity.

[Authors' reply]. *We agree with the reviewer that our data on a potential role of LGCs' on T cell activation is based on increased surface expression of B7-H3 and the unique CD3+ cluster forming ability of LGCs. In order to check for further markers of antigen presentation, we have performed MHC-1 and MHC-2 quantification by ImageStream in 3 types of MGCs (see below).*

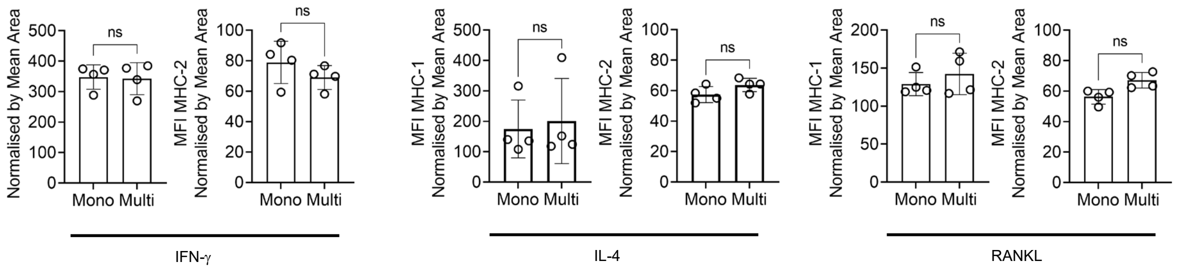

Although there was no difference in MHC-I/MHC-2 between the mononucleated and multinucleated macrophages, the mean fluorescent intensity (MFI) range was the highest in IFN-γ-stimulated macrophages, suggesting that LGCs may be better equipped for antigen presentation than the other 2 types of MGCs. A more comprehensive analysis of antigen presentation requires enzymatic digestion and isolation and phenotyping of LGCs from clusters in vitro and human tissues in vivo. This is a program of research that we have initiated as part of a separate study, which will focus on the in vivo relevance of the current findings such as the unique Ag presentation ability of LGCs in a non-sterile tissue environment.

5 Figure 6 clearly shows that mature multinucleated osteoclasts exhibit increased ATP production and maximal respiration. However, the glycolytic pathway did not differ between mononuclear and multinuclear osteoclasts. No explanation for this observation has been provided. It is easy to understand that osteoclasts acquire ATP through aerobic respiration during multinucleation. But how NADPH, which is essential for its redox reaction, is produced? Is it by acquiring αKG from the glutamine pathway?

[Authors' reply]. *This is a point worth expending (see also discussion; highlighted text). Osteoclast multinucleation is characterized by increased mitochondrial gene expression which also translates into increased spare respiratory capacity (SRC or maximal respiration). This metabolic rewiring does not modify glycolysis and basal respiration rate. As the reviewer correctly states, increased SRC may be a way to supply more ATP to the energy-demanding polykaryon.*

As per the production of NAD(P)H as an electron source for ETC, it could indeed be through glutamine rather than glucose usage in multinucleated osteoclasts. Furthermore, as iron is an essential cofactor for ETC activity through activity of iron-sulfur clusters, the mitochondrial

concentration of iron is likely to be critical for the mitochondrial activity of multinucleated osteoclasts (see also discussion).

Minor comments:

#6 Supplementary tables 1-6 were not provided.

[Authors' reply]. *We apologize for this. The revised versions of supplementary tables are provided as part of the revised manuscript.*

#7 Figure 2D right panel, difficult to see DAPI+ nuclei.

[Authors' reply]. *Thanks for pointing this out. We have now replaced Figure 2D with a more pronounced DAPI+ nuclei.*

Reviewer #2 (Significance (Required)):

Although it is well known that multinucleation of cells constantly occurs, especially in osteoclasts, skeletal muscle, and trophoblasts of the placenta, the biological significance of multinucleation and the intracellular functions of multinucleation are not well understood. In this unique study, three types of multinucleated cells were generated from human peripheral blood to elucidate the genetic and functional differences between mononucleated and multinucleated cells. Furthermore, by demonstrating the possibility that the morphological peculiarity of multinucleation can regulate cell function, this paper provides clues to understanding the underlying biology of multinucleated cells and how they maintain cell function in homeostatic and pathological settings.

[Authors' reply]. *We thank the reviewer for finding our study unique and biologically meaningful. We also thank the reviewer for all the suggestions that improved significantly the overall message of the manuscript.*

Reviewer #3 (Evidence, reproducibility and clarity (Required)):

Summary:

The manuscript of Ahmadzadeh and Pereira et al is an interesting study of the fusion process key to the formation of multinucleated giant cells (MGCs). Our current ability to discriminate between different types of MGCs is limited, and there are gaps in our understanding of the molecular determinants of cell fusion. In this study, the authors isolated different MGC variants - osteoclasts, Langhans giant cells (LGCs) and foreign body giant cells (FBGCs) and identified common, as well as MGC-specific genes and pathways involved in the process of cell fusion. The approach of isolating and comparing different types of MGCs is novel, and the manuscript is well presented and written. However, due to the in vitro nature of the study, the physiological significance of the findings is unclear. I have further minor and major points for the authors to address, as detailed below.

Minor comments:

1. The approach to isolate the different MGCs using FACS and imaging technique is highly novel. However the difference between MGC subtypes isolated isn't immediately apparent beyond the morphological comparisons. In my opinion some of the results of MGC-specific assays from Figures 4, 5 and 6 can be included in Figure 1, e.g. TRAP staining and hydroxyapatite resorption for osteoclasts, to provide evidence of purity and specificity of each MGC subtype early on in the manuscript. Classical or canonical genes associated with each MGC subtype can also be highlighted in the volcano plots in Figure 1C, e.g ACP5, CTSK, TNFRSF11A for osteoclasts.

[Authors' reply]. *We thank the reviewer for this point and we agree it is important to highlight markers for each polykaryon early in the manuscript. In accordance with this reviewers' comment (and also with Reviewer 1's point), we first verified existence of lineage-dependent factors and markers of LGCs and FBGCs as these cells are relatively less well-defined compared to osteoclasts. (New Supplementary Figure 1B and C and New Supplementary Table 1). As expected, and in line with the lineage-determining effects, the transcriptomics comparison between mononucleated/multinucleated IFN-γ and IL-4-differentiated macrophages showed predominance of IFN-γ and IL-4-related pathways, respectively (New Supplementary Figure 1B and C and New Supplementary Table 1). Among known LGC and FBGC markers, we confirmed up-regulation of CCL7 [1] and CD86 [2], respectively (New Supplementary Table 1). We have added this information in the revised manuscript (see highlighted text). Osteoclast phenotyping is provided by TRAP staining and resorption assay (Figure 6C) and we also confirm that CTSK is indeed significantly up-regulated upon multinucleation (LogFc=1.69; P=9.2 x 10E-6; highlighted in the revised manuscript).*

2. The overall decrease in phagocytic identity of all the MGCs, and the specific upregulation of phagocytic pathways in the FBGCs are conflicting. Are there subsets of phagocytic pathways that were down and upregulated during the formation of FBGCs?

[Authors' reply]. *This is a very good point. As the reviewer indicates, the results suggest that subsets of phagocytic pathways are changed upon multinucleation. All three types of MGCs show a downregulation of transcripts that belong to Fc receptors and complement C1Q family. However only FBGCs show an up-regulation of S. Aureus bioparticle-mediated phagocytosis. Hence the exact surface receptors responsible for this pathogen clearance remain to be identified. FBGC phagocytosis is a complex process including non-canonical phagocytosis pathways and participation of increased membrane area and endoplasmic reticulum [5, 6]. Whether these pathways are specifically induced in human FBGCs remain to be identified. We now discuss this point in the revised manuscript (see highlighted text in Discussion).*

3. What are the identities of the mononuclear cells in each of the MGC experiment? They appeared to be quite heterogeneous based on the DEGs identified, beyond the common phagocyte signature. Can the authors comment on the difference between the mononuclear cells and whether this will affect the DEG analysis?

[Authors' reply]. *This is also a very relevant point that we now address in the revised manuscript (New Supplementary Figure 1B and C; New Supplementary Table 1 and highlighted revised text in Results). The reviewer is correct that MGC-specific pathways are in line with the known function of each polykaryon (Figure 4A, 5A and 6A). To what extent lineage-dependent effects (e.g. IFN-γ and IL-4) are conserved between the mononucleated and multinucleated state is yet to be determined. In order to address this point, we compared DEG in IFN-γ and IL-4-differentiated mononucleated macrophages to the ones obtained in multinucleated macrophages (New Supplementary Figure 1B and C; New Supplementary Table 1). The results showed that the multinucleated cell state preserves the majority of the lineage-dependent pathways which are very significantly represented at the mononucleated cell state (e.g. IFN-γ and IL-4-related pathways). Interestingly, although less significant, this analysis also showed pathways that were specific to the mononucleated or multinucleated state in IFN-γ-differentiated macrophages when compared to IL-4-differentiated ones and vice versa. (Supplementary Figure 1B and C). For instance, TRAF3-dependent IRF activation pathway is specific to mononucleated IFN-γ-differentiated macrophages (Supplementary Figure 1B).*

4. The authors should also frame/discuss the findings in the context of diagnostic and therapeutic potentials to highlight the clinical significance of this study.

[Authors' reply]. *We thank the reviewer for this point and we now discuss our results from a clinical/diagnostic perspective (see highlighted text in the Discussion and below).*

From a clinical perspective, since lysosome-regulated intracellular iron homeostasis appears to be a general condition for macrophage multinucleation across different tissues, its blockade may hold therapeutic potential. However, it is still unclear whether granulomatous disease can benefit from targeting LGC fusion. For non-granulomatous inflammatory diseases, inhibiting MGC formation by targeting lysosomes may be a therapeutic avenue. This approach would avoid FBGC-related adverse effects during foreign body reaction or inhibit the formation of MGCs of white adipose tissue during obesity. v-ATPase inhibitors have been previously proposed to inhibit osteoclast activity and bone resorption [7] so their selective targeting in the lysosomal compartment may be generalized to other MGCs such as FBGCs. In addition to potential clinical translation, the results presented in this study require confirmation in tissues originating from human pathology involving MGCs.

Major comments:

• As mentioned before, the physiological significance of the findings is unclear. Some form of in vivo data is needed to support some of the key conclusions of the study, e.g validating some of the markers of the pathways identified (common and MGC subtype-specific), and the role of lysosome-mediated iron homeostasis in multinucleation. The authors can make use of the FACs and imaging approaches they developed to look at MGCs in relevant tissues.

[Authors' reply]. *This is an important point that we would like to explore in a comprehensive way. We have initiated a 2-year program to undertake a Multiplexed Immunohistochemistry (mIHC)*

using MILAN (Multiple Iterative Labeling by Antibody Neodeposition) https://www.lpcm.be/multiplex-ihc-milan approach in human biopsies using >100 antibodies. The current study is pivotal in selecting the gene targets (i.e. common and MGC-specific markers) for prioritization. We foresee to gain critical pathophysiological information about the tissue characteristics of MGCs. The reviewer would acknowledge that these high-throughput and biopsy-based initiatives are lengthy and not the primary scope of our current findings which set the foundation of major cellular events governing multinucleation in macrophages.

Reviewer #3 (Significance (Required)):

Significance:

• The approach of isolating and comparing different types of MGCs is novel, and the findings certainly improved our understanding of the fusion processes of MGCs. However the physiological role of these processes in health and disease that involve MGCs is still unclear due to the lack of in vivo data. The findings were discussed in quite a bit of detail in the context of current literature, though clinical impact was not explored.

[Authors' reply]. *We are grateful to Reviewer 3 for raising relevant and constructive points regarding the main findings. His/her review significantly improved the clarity of the overall manuscript.*

We recognize our study lacks human clinical association but we highlight the prospective translatability of our findings and the usage of donor-based human macrophages throughout the manuscript. As also recommended by Reviewer 1 in his/her cross-consultation, we discuss the potential clinical impact of our findings in the Discussion of our revised manuscript.

• My background is bone biology with a very keen interest in osteoclast biology so arguably my knowledge on other MGCs eg LGCs and FBGCs is limited.

References

1. Chen Y, Jiang H, Xiong J, Shang J, Chen Z, Wu A, Wang H: **Insight into the Molecular Characteristics of Langhans Giant Cell by Combination of Laser Capture Microdissection and RNA Sequencing**. *J Inflamm Res* 2022, **15**:621-634.
2. McNally AK, Anderson JM: **Foreign body-type multinucleated giant cells induced by interleukin-4 express select lymphocyte co-stimulatory molecules and are phenotypically distinct from osteoclasts and dendritic cells**. *Exp Mol Pathol* 2011, **91**(3):673-681.
3. Petrany MJ, Swoboda CO, Sun C, Chetal K, Chen X, Weirauch MT, Salomonis N, Millay DP: **Single-nucleus RNA-seq identifies transcriptional heterogeneity in multinucleated skeletal myofibers**. *Nat Commun* 2020, **11**(1):6374.
4. Pereira M, Ko JH, Logan J, Protheroe H, Kim KB, Tan ALM, Croucher PI, Park KS, Rotival M, Petretto E *et al*: **A trans-eQTL network regulates osteoclast multinucleation and bone mass**. *Elife* 2020, **9**.
5. McNally AK, Anderson JM: **Multinucleated giant cell formation exhibits features of phagocytosis with participation of the endoplasmic reticulum**. *Exp Mol Pathol* 2005, **79**(2):126-135.
6. Milde R, Ritter J, Tennent GA, Loesch A, Martinez FO, Gordon S, Pepys MB, Verschoor A, Helming L: **Multinucleated Giant Cells Are Specialized for Complement-Mediated Phagocytosis and Large Target Destruction**. *Cell Rep* 2015, **13**(9):1937-1948.
7. Qin A, Cheng TS, Pavlos NJ, Lin Z, Dai KR, Zheng MH: **V-ATPases in osteoclasts: structure, function and potential inhibitors of bone resorption**. *Int J Biochem Cell Biol* 2012, **44**(9):1422-1435.

1st Editorial Decision **7th Nov 2022**

Dear Prof. Behmoaras,

Thank you for transferring your revised manuscript from Review Commons to EMBO reports. I have now received the reports from the three referees that had assessed your study at RC that were asked to re-evaluate the manuscript. Please find their comments below. As you will see, the referees now fully support the publication of your study.

Before I can proceed with formal acceptance, the manuscript needs formatting according to our journal style, which I ask you to do in a final revision. Please contact me to discuss the revision (also by video chat) if you have questions or comments.

Please carefully review the instructions that follow below.

PLEASE NOTE THAT upon resubmission revised manuscripts are subjected to an initial quality control prior to exposition to re-review. Upon failure in the initial quality control, the manuscripts are sent back to the authors, which may lead to delays. Frequent reasons for such a failure are the lack of the data availability section (please see below) and the presence of statistics based on n=2 (the authors are then asked to present scatter plots or provide more data points).

When submitting your final revised manuscript, we will require:

1) a .docx formatted version of the final manuscript text (including legends for main figures, EV figures and tables), but without the figures included. Figure legends should be compiled at the end of the manuscript text.

2) individual production quality figure files as .eps, .tif, .jpg (one file per figure), of main figures and EV figures. Please upload these as separate, individual files upon re-submission.

The Expanded View format, which will be displayed in the main HTML of the paper in a collapsible format, has replaced the Supplementary information. You can submit up to 5 images as Expanded View. Please follow the nomenclature Figure EV1, Figure EV2 etc. The figure legend for these should be included in the main manuscript document file in a section called Expanded View Figure Legends after the main Figure Legends section. Additional Supplementary material should be supplied as a single pdf file labeled Appendix. The Appendix should have page numbers and needs to include a table of content on the first page (with page numbers) and legends for all content. Please follow the nomenclature Appendix Figure Sx, Appendix Table Sx etc. throughout the text, and also label the figures and tables according to this nomenclature.

For more details, please refer to our guide to authors:
http://www.embopress.org/page/journal/14693178/authorguide#manuscriptpreparation

Please consult our guide for figure preparation:
http://wol-prod-cdn.literatumonline.com/pb-assets/embo-site/EMBOPress_Figure_Guidelines_061115-1561436025777.pdf

See also the guidelines for figure legend preparation:
https://www.embopress.org/page/journal/14693178/authorguide#figureformat

3) a complete author checklist, which you can download from our author guidelines (https://www.embopress.org/page/journal/14693178/authorguide). Please insert page numbers in the checklist to indicate where the requested information can be found in the manuscript. The completed author checklist will also be part of the RPF.

Please also follow our guidelines for the use of living organisms, and the respective reporting guidelines:
http://www.embopress.org/page/journal/14693178/authorguide#livingorganisms

4) that primary datasets produced in this study (e.g. RNA-seq, ChIP-seq, structural and array data) are deposited in an appropriate public database. If no primary datasets have been deposited, please also state this in a dedicated section (e.g. 'No primary datasets have been generated and deposited'), see below.

See also: http://embor.embopress.org/authorguide#datadeposition

Please remember to provide a reviewer password if the datasets are not yet public.

The accession numbers and database should be listed in a formal "Data Availability" section (placed after Materials & Methods) that follows the model below. This is now mandatory (like the COI statement). Please note that the Data Availability Section is restricted to new primary data that are part of this study. This section is mandatory. As indicated above, if no primary datasets have been deposited, please state this in this section

Data availability

The datasets produced in this study are available in the following databases:

- RNA-Seq data: Gene Expression Omnibus GSE46843 (https://www.ncbi.nlm.nih.gov/geo/query/acc.cgi?acc=GSE46843)
- [data type]: [name of the resource] [accession number/identifier/doi] ([URL or identifiers.org/DATABASE:ACCESSION])

*** Note - All links should resolve to a page where the data can be accessed. ***

Moreover, I have these editorial requests:

6) We now request the publication of original source data with the aim of making primary data more accessible and transparent to the reader. Our source data coordinator will contact you to discuss which figure panels we would need source data for and will also provide you with helpful tips on how to upload and organize the files.

7) Our journal encourages inclusion of *data citations in the reference list* to directly cite datasets that were re-used and obtained from public databases. Data citations in the article text are distinct from normal bibliographical citations and should directly link to the database records from which the data can be accessed. In the main text, data citations are formatted as follows: "Data ref: Smith et al, 2001" or "Data ref: NCBI Sequence Read Archive PRJNA342805, 2017". In the Reference list, data citations must be labeled with "[DATASET]". A data reference must provide the database name, accession number/identifiers and a resolvable link to the landing page from which the data can be accessed at the end of the reference. Further instructions are available at: http://www.embopress.org/page/journal/14693178/authorguide#referencesformat

8) Regarding data quantification and statistics, please make sure that the number "n" for how many independent experiments were performed, their nature (biological versus technical replicates), the bars and error bars (e.g. SEM, SD) and the test used to calculate p-values is indicated in the respective figure legends (also for potential EV figures and all those in the final Appendix). Please also check that all the p-values are explained in the legend, and that these fit to those shown in the figure. Please provide statistical testing where applicable. Please add complete statistical testing to all diagrams (for main, EV figures). Please also indicate (e.g. with n.s.) if testing was performed, but the differences are not significant. Please avoid the phrase 'independent experiment', but clearly state if these were biological or technical replicates. In case n=2, please show the data as separate datapoints without error bars and statistics. See also:
http://www.embopress.org/page/journal/14693178/authorguide#statisticalanalysis

9) Please also note our reference format:
http://www.embopress.org/page/journal/14693178/authorguide#referencesformat

10) For all microscopic images, please add scale bars of similar style and thickness to all the microscopic images, using clearly visible black or white bars (depending on the background). Please place these in the lower right corner of the images. Please do not write on or near the bars in the image but define the size in the respective figure legend.

11) We updated our journal's competing interests policy in January 2022 and request authors to consider both actual and perceived competing interests. Please review the policy https://www.embopress.org/competing-interests and update your competing interests if necessary. Please name this section 'Disclosure and Competing Interests Statement' and put it after the Acknowledgements section.

12) Please make sure that all the funding information is entered into the online submission system and is complete and similar to the one in the acknowledgements section of the manuscript text file.

13) We now use CRediT to specify the contributions of each author in the journal submission system. CRediT replaces the author contribution section. Please use the free text box to provide more detailed descriptions. See also guide to authors:
https://www.embopress.org/page/journal/14693178/authorguide#authorshipguidelines

14) Please provide a title with not more than 100 characters (including spaces).

15) Please provide the abstract with not more than 175 words.

16) Please add up to 5 keywords to the title page (below the abstract).

17) Please make sure that the manuscript sections are ordered like this:
Title page - Abstract - Introduction - Results - Discussion - Materials and Methods - Data availability section (DAS) - Acknowledgements - Disclosure and Competing Interests Statement - References - Figure legends - Expanded View Figure legends

18) The supplementary tables S1-S7 are datasets. Please upload these as Dataset EVx as original Excel file. Please put a title and a legend on the first TAB of the Excel file. Finally, please also change the callouts for these tables to Dataset EVx.

19) Please upload Supplementary Table S8 as main Table (named Table 1) or add this to the main text file with a title and a legend and a callout in the methods section.

In addition, I would need from you:
- a short, two-sentence summary of the manuscript (not more than 35 words).
- three to four short one sentence bullet points highlighting the key findings of your study.
- a schematic summary figure (synopsis image) in jpeg or tiff format with the exact width of 550 pixels and a height of not more than 400 pixels that can be used as a visual synopsis on our website.

I look forward to seeing a revised version of your manuscript when it is ready. Please let me know if you have questions or comments regarding the revision.

Please use this link to submit your revision: https://embor.msubmit.net/cgi-bin/main.plex

Best,

Achim Breiling
Senior Editor
EMBO Reports

Referee #1:

The authors have addressed the reviewers' concerns. Thus, the revised manuscript is acceptable for publication. I do not have further comments.

Referee #2:

The authors have done a great job addressing all the comments. I'm in support of this manuscript going ahead for publication in EMBO reports.

Referee #3:

For me, the authors have answered all remaining issues.

Dear Achim,

Thank you for your decision letter (EMBOR-2022-56310V1| [RC-2022-01638] [REV]) whereby you informed us all 3 reviewers accepted our manuscript. For the formal acceptance, please find our revised manuscript as well as all the supporting documents required in your letter.

With best wishes,

Jacques Behmoaras (for the authors)

1st Revision - Editorial Decision 7th Dec 2022

Dear Prof. Behmoaras,

Thank you for the submission of your revised manuscript to our editorial offices. We are nearly done. Before I will proceed with formal acceptance, I have these further editorial requests I also ask you to address in a final revised manuscript:

- We updated our journal's competing interests policy in January 2022 and request authors to consider both actual and perceived competing interests. Please review the policy https://www.embopress.org/competing-interests and update your competing interests if necessary. Please name this section 'Disclosure and Competing Interests Statement' and put it after the Acknowledgements section.

- Then, please order the manuscript sections like this:
Title page - Abstract - Keywords - Introduction - Results - Discussion - Materials and Methods - Data availability section - Acknowledgements (including funding information) - Disclosure and Competing Interests Statement - References - Figure legends - Expanded View Figure legends - Tables

- Please move Table 1 to the end of the manuscript.

- Please make sure that the figure panels are called out separately and in a sequential manner (alphabetical, as they show up in the figure). Presently, panels EV 2A,B are called out before panel EV1B. Please check. In case, please change the order of panels in the figure.

- There are callouts for Supplementary Figures (mainly in the methods section., it seems) Please correct these to Figure EVx.

- In Figure 6C the lower panels in the first two rows (IFN-g and IL-4) are completely empty, there is no image noise. For microscopy images, there should be some image noise. The source data is exactly the same and has no image noise. Could you please comment on this?

- Please provide a completed source data checklist (attached again).

- No source data for Fig 6D was provided. Please do that or provide an explanation (in the checklist).

- Please note that all corresponding authors are required to supply an ORCID ID for their name upon submission of a revised manuscript. Please do that for co-corresponding author Carine Wouters. Please find instructions on how to link your ORCID ID to your account in our manuscript tracking system in our Author guidelines:
http://www.embopress.org/page/journal/14693178/authorguide#authorshipguidelines

- Please find attached a word file of the manuscript text (provided by our publisher) with changes we ask you to include in your final manuscript text. Please use the attached file as basis for the revision and provide your final manuscript file with track changes, in order that I can see any modifications done.

I look forward to seeing the final revised version of your manuscript when it is ready. Please let me know if you have questions regarding the revision.

Please use this link to submit your revision: https://embor.msubmit.net/cgi-bin/main.plex

Achim Breiling
Senior Editor
EMBO Reports

The authors have addressed all minor editorial requests.

Dear Achim,

Many thanks. All completed. Here are the specific responses (in red):

- We updated our journal's competing interests policy in January 2022 and request authors to consider both actual and perceived competing interests. Please review the policy https://www.embopress.org/competing-interests<https://ddec1-0-en-ctp.trendmicro.com:443/wis/clicktime/v1/query?url=https%3a%2f%2fwww.embopress.org%2fcompeting%2dinterests&umid=af20d579-8b43-4d42-b71b-4a1d96d2f94b&auth=3eee6d57317a38631073652579c10dc620ca2b41-cdf465e1a0ab7640b4b7b4c422bdc9bc28669f50> and update your competing interests if necessary. Please name this section 'Disclosure and Competing Interests Statement' and put it after the Acknowledgements section.

DONE

- Then, please order the manuscript sections like this:
Title page - Abstract - Keywords - Introduction - Results - Discussion - Materials and Methods - Data availability section - Acknowledgements (including funding information) - Disclosure and Competing Interests Statement - References - Figure legends - Expanded View Figure legends - Tables

DONE

- Please move Table 1 to the end of the manuscript.

DONE

- Please make sure that the figure panels are called out separately and in a sequential manner (alphabetical, as they show up in the figure). Presently, panels EV 2A,B are called out before panel EV1B. Please check. In case, please change the order of panels in the figure.

I changed the order of panels in the figure. I corrected the corresponding call outs and also corrected the expanded view figure legends - all indicated as track changes

- There are callouts for Supplementary Figures (mainly in the methods section., it seems) Please correct these to Figure EVx.

Corrected.

- In Figure 6C the lower panels in the first two rows (IFN-g and IL-4) are completely empty, there is no image noise. For microscopy images, there should be some image noise. The source data is exactly the same and has no image noise. Could you please comment on this?

These are high-contrast images and show the resorbed areas by osteoclasts in black against a white background. The output are binary images of each individual well that is subjected to automated analysis. A total white background represents no resorptive activity of the cell which is the case for IFN-g (LGCs) and IL-4 (FBGCs).

- Please provide a completed source data checklist (attached again).

DONE

- No source data for Fig 6D was provided. Please do that or provide an explanation (in the checklist).

Apologies for the mistake. Uploaded now.

- Please note that all corresponding authors are required to supply an ORCID ID for their name upon submission of a revised manuscript. Please do that for co-corresponding author Carine Wouters. Please find instructions on how to link your ORCID ID to your account in our manuscript tracking system in our Author guidelines: http://www.embopress.org/page/journal/14693178/authorguide#authorshipguidelines<https://ddec1-0-en-ctp.trendmicro.com:443/wis/clicktime/v1/query?url=http%3a%2f%2fwww.embopress.org%2fpage%2fjournal%2f14693178%2fauthorguide%23authorshipguidelines&umid=af20d579-8b43-4d42-b71b-4a1d96d2f94b&auth=3eee6d57317a38631073652579c10dc620ca2b41-0acc92e0ce3472ecde99d4ca5b66b0c9be3158b7>

OK. Carine Wouters ORCID ID is 0000-0002-6426-8845 https://orcid.org/0000-0002-6426-8845?lang=en
I have told the author to do this on the manuscript tracking system - if you can link the above ID to her name/email, that would be great too

- Please find attached a word file of the manuscript text (provided by our publisher) with changes we ask you to include in your final manuscript text. Please use the attached file as basis for the revision and provide your final manuscript file with track changes, in order that I can see any modifications done.

OK. This is now uploaded. I also responded to specific queries regarding biological/technical replicates - all changes indicated as track changes.

Many thanks,

Jacques

2nd Revision - Editorial Decision **9th Dec 2022**

Prof. Jacques Behmoaras
Duke NUS Singapore
8 College Road
Singapore 169857
Singapore

Dear Prof. Behmoaras,

I am very pleased to accept your manuscript for publication in the next available issue of EMBO reports. Thank you for your contribution to our journal.

At the end of this email I include important information about how to proceed. Please ensure that you take the time to read the information and complete and return the necessary forms to allow us to publish your manuscript as quickly as possible.

As part of the EMBO publication's Transparent Editorial Process, EMBO reports publishes online a Review Process File to accompany accepted manuscripts. As you are aware, this File will be published in conjunction with your paper and will include the referee reports, your point-by-point response and all pertinent correspondence relating to the manuscript.

If you do NOT want this File to be published, please inform the editorial office within 2 days, if you have not done so already, otherwise the File will be published by default [contact: emboreports@embo.org]. If you do opt out, the Review Process File link will point to the following statement: "No Review Process File is available with this article, as the authors have chosen not to make the review process public in this case."

Should you be planning a Press Release on your article, please get in contact with emboreports@wiley.com as early as possible, in order to coordinate publication and release dates.

Thank you again for your contribution to EMBO reports and congratulations on a successful publication. Please consider us again in the future for your most exciting work.

Yours sincerely,

Achim Breiling
Senior Editor
EMBO Reports

****

THINGS TO DO NOW:

You will receive proofs by e-mail approximately 2-3 weeks after all relevant files have been sent to our Production Office; you should return your corrections within 2 days of receiving the proofs.

EMBO Press participates in many Publish and Read agreements that allow authors to publish Open Access with reduced/no publication charges. Check your eligibility: https://authorservices.wiley.com/author-resources/Journal-Authors/open-access/affiliation-policies-payments/index.html

Please inform us if there is likely to be any difficulty in reaching you at the above address at that time. Failure to meet our deadlines may result in a delay of publication, or publication without your corrections.

All further communications concerning your paper should quote reference number EMBOR-2022-56310V3 and be addressed to emboreports@wiley.com.

Should you be planning a Press Release on your article, please get in contact with emboreports@wiley.com as early as possible, in order to coordinate publication and release dates.

***

Rev_Com_number: RC-2022-01638
New_manu_number: EMBOR-2022-56310V3
Corr_author: Behmoaras
Title: Multinucleation resets human macrophages for specialized functions at the expense of their identity

EMBO Press Author Checklist

Corresponding Author Name: Jacques Behmoaras	
Journal Submitted to: EMBO Reports	
Manuscript Number: EMBOR-2022-56310V1	

USEFUL LINKS FOR COMPLETING THIS FORM
The EMBO Journal - Author Guidelines
EMBO Reports - Author Guidelines
Molecular Systems Biology - Author Guidelines
EMBO Molecular Medicine - Author Guidelines

Reporting Checklist for Life Science Articles (updated January 2022)
This checklist is adapted from Materials Design Analysis Reporting (MDAR) Checklist for Authors. MDAR establishes a minimum set of requirements in transparent reporting in the life sciences (see Statement of Task: 10.31222/osf.io/9sm4x). Please follow the journal's guidelines in preparing your manuscript.
Please note that a copy of this checklist will be published alongside your article.

Abridged guidelines for figures
1. Data
The data shown in figures should satisfy the following conditions:
- the data were obtained and processed according to the field's best practice and are presented to reflect the results of the experiments in an accurate and unbiased manner.
- ideally, figure panels should include only measurements that are directly comparable to each other and obtained with the same assay.
- plots include clearly labeled error bars for independent experiments and sample sizes. Unless justified, error bars should not be shown for technical replicates.
- if n<5, the individual data points from each experiment should be plotted. Any statistical test employed should be justified.
- Source Data should be included to report the data underlying figures according to the guidelines set out in the authorship guidelines on Data Presentation.

2. Captions
Each figure caption should contain the following information, for each panel where they are relevant:
- a specification of the experimental system investigated (eg cell line, species name).
- the assay(s) and method(s) used to carry out the reported observations and measurements.
- an explicit mention of the biological and chemical entity(ies) that are being measured.
- an explicit mention of the biological and chemical entity(ies) that are altered/varied/perturbed in a controlled manner.
- the exact sample size (n) for each experimental group/condition, given as a number, not a range;
- a description of the sample collection allowing the reader to understand whether the samples represent technical or biological replicates (including how many animals, litters, cultures, etc.).
- a statement of how many times the experiment shown was independently replicated in the laboratory.
- definitions of statistical methods and measures:
  - common tests, such as t-test (please specify whether paired vs. unpaired), simple χ2 tests, Wilcoxon and Mann-Whitney tests, can be unambiguously identified by name only, but more complex techniques should be described in the methods section;
  - are tests one-sided or two-sided?
  - are there adjustments for multiple comparisons?
  - exact statistical test results, e.g., P values = x but not P values < x;
  - definition of 'center values' as median or average;
  - definition of error bars as s.d. or s.e.m.

Please complete ALL of the questions below.
Select "Not Applicable" only when the requested information is not relevant for your study.

Materials

Newly Created Materials	Information included in the manuscript?	In which section is the information available? (Reagents and Tools Table, Materials and Methods, Figures, Data Availability Section)
New materials and reagents need to be available; do any restrictions apply?	Not Applicable	

Antibodies	Information included in the manuscript?	In which section is the information available? (Reagents and Tools Table, Materials and Methods, Figures, Data Availability Section)
For **antibodies** provide the following information:<br>- Commercial antibodies: RRID (if possible) or supplier name, catalogue number and or/clone number<br>- Non-commercial: RRID or citation	Yes	Material and Methods (p13)

DNA and RNA sequences	Information included in the manuscript?	In which section is the information available? (Reagents and Tools Table, Materials and Methods, Figures, Data Availability Section)
**Short novel DNA or RNA including primers, probes**: provide the sequences.	Yes	Material and Methods (p14)

Cell materials	Information included in the manuscript?	In which section is the information available? (Reagents and Tools Table, Materials and Methods, Figures, Data Availability Section)
**Cell lines:** Provide species information, strain. Provide accession number in repository **OR** supplier name, catalog number, clone number, and/**OR** RRID.	Not Applicable	
**Primary cultures:** Provide species, strain, sex of origin, genetic modification status.	Yes	Material and Methods (p11)
Report if the cell lines were recently **authenticated** (e.g., by STR profiling) and tested for mycoplasma contamination.	Not Applicable	

Experimental animals	Information included in the manuscript?	In which section is the information available? (Reagents and Tools Table, Materials and Methods, Figures, Data Availability Section)
**Laboratory animals or Model organisms:** Provide species, strain, sex, age, genetic modification status. Provide accession number in repository **OR** supplier name, catalog number, clone number, **OR** RRID.	Not Applicable	
**Animal observed in or captured from the field:** Provide species, sex, and age where possible.	Not Applicable	
Please detail **housing and husbandry conditions**.	Not Applicable	

Plants and microbes	Information included in the manuscript?	In which section is the information available? (Reagents and Tools Table, Materials and Methods, Figures, Data Availability Section)
**Plants:** provide species and strain, ecotype and cultivar where relevant, unique accession number if available, and source (including location for collected wild specimens).	Not Applicable	
**Microbes:** provide species and strain, unique accession number if available, and source.	Not Applicable	

Human research participants	Information included in the manuscript?	In which section is the information available? (Reagents and Tools Table, Materials and Methods, Figures, Data Availability Section)
If collected and within the bounds of privacy constraints report on age, sex and gender or ethnicity for all study participants.	Not Applicable	

Core facilities	Information included in the manuscript?	In which section is the information available? (Reagents and Tools Table, Materials and Methods, Figures, Data Availability Section)
If your work benefited from core facilities, was their service mentioned in the acknowledgments section?	Not Applicable	

Design

Study protocol	Information included in the manuscript?	In which section is the information available? (Reagents and Tools Table, Materials and Methods, Figures, Data Availability Section)
If study protocol has been **pre-registered, provide DOI in the manuscript**. For clinical trials, provide the trial registration number **OR** cite DOI.	Not Applicable	
Report the **clinical trial registration number** (at ClinicalTrials.gov or equivalent), where applicable.	Not Applicable	

Laboratory protocol	Information included in the manuscript?	In which section is the information available? (Reagents and Tools Table, Materials and Methods, Figures, Data Availability Section)
Provide DOI OR other citation details if **external detailed step-by-step protocols** are available.	Not Applicable	

Experimental study design and statistics	Information included in the manuscript?	In which section is the information available? (Reagents and Tools Table, Materials and Methods, Figures, Data Availability Section)
Include a statement about **sample size** estimate even if no statistical methods were used.	Yes	Figure Legends (p17-19)
Were any steps taken to minimize the effects of subjective bias when allocating animals/samples to treatment (e.g. **randomization procedure**)? If yes, have they been described?	Yes	Material and Methods (p11)
Include a statement about **blinding** even if no blinding was done.	Yes	Material and Methods (p14)
Describe **inclusion/exclusion criteria** if samples or animals were excluded from the analysis. Were the criteria pre-established?<br><br>If sample or data points were omitted from analysis, report if this was due to attrition or intentional exclusion and provide justification.	Not Applicable	
For every figure, are **statistical tests** justified as appropriate? Do the data meet the assumptions of the tests (e.g., normal distribution)? Describe any methods used to assess it. Is there an estimate of variation within each group of data? Is the variance similar between the groups that are being statistically compared?	Yes	Figure Legends and Material and Methods (p16)

Sample definition and in-laboratory replication	Information included in the manuscript?	In which section is the information available? (Reagents and Tools Table, Materials and Methods, Figures, Data Availability Section)
In the figure legends: state number of times the experiment was **replicated** in laboratory.	Yes	Figure Legends (p17-19)
In the figure legends: define whether data describe **technical or biological replicates**.	Yes	Figure Legends (p17-19)

Ethics

Ethics	Information included in the manuscript?	In which section is the information available? (Reagents and Tools Table, Materials and Methods, Figures, Data Availability Section)
Studies involving **human participants**: State details of **authority granting ethics approval** (IRB or equivalent committee(s), provide reference number for approval.	Yes	Material and Methods (p11)
Studies involving **human participants**: Include a statement confirming that **informed consent** was obtained from all subjects and that the experiments conformed to the principles set out in the WMA Declaration of Helsinki and the Department of Health and Human Services Belmont Report.	Yes	Material and Methods (p11)
Studies involving **human participants:** For publication of **patient photos**, include a statement confirming that consent to publish was obtained.	Not Applicable	
Studies involving experimental **animals**: State details of **authority granting ethics approva**l (IRB or equivalent committee(s), provide reference number for approval. Include a statement of compliance with ethical regulations.	Not Applicable	
Studies involving **specimen and field samples:** State if relevant **permits** obtained, provide details of authority approving study; if none were required, explain why.	Not Applicable	

Dual Use Research of Concern (DURC)	Information included in the manuscript?	In which section is the information available? (Reagents and Tools Table, Materials and Methods, Figures, Data Availability Section)
Could your study fall under dual use research restrictions? Please check biosecurity documents and list of **select agents and toxins** (CDC): https://www.selectagents.gov/sat/list.htm	Not Applicable	
If you used a select agent, is the security level of the lab appropriate and reported in the manuscript?	Not Applicable	
If a study is subject to dual use research of concern regulations, is the name of the **authority granting approval and reference number** for the regulatory approval provided in the manuscript?	Not Applicable	

Reporting

The MDAR framework recommends adoption of discipline-specific guidelines, established and endorsed through community initiatives. Journals have their own policy about requiring specific guidelines and recommendations to complement MDAR.

Adherence to community standards	Information included in the manuscript?	In which section is the information available? (Reagents and Tools Table, Materials and Methods, Figures, Data Availability Section)
State if relevant guidelines or checklists (e.g., **ICMJE, MIBBI, ARRIVE, PRISMA**) have been followed or provided.	Not Applicable	
For **tumor marker prognostic studies**, we recommend that you follow the **REMARK** reporting guidelines (see link list at top right). See author guidelines, under 'Reporting Guidelines'. Please confirm you have followed these guidelines.	Not Applicable	
For **phase II and III randomized controlled trials**, please refer to the **CONSORT** flow diagram (see link list at top right) and submit the CONSORT checklist (see link list at top right) with your submission. See author guidelines, under 'Reporting Guidelines'. Please confirm you have submitted this list.	Not Applicable	

Data Availability

Data availability	Information included in the manuscript?	In which section is the information available? (Reagents and Tools Table, Materials and Methods, Figures, Data Availability Section)
Have **primary datasets** been deposited according to the journal's guidelines (see 'Data Deposition' section) and the respective accession numbers provided in the Data Availability Section?	Yes	Data Availibility Section (p16)
Were **human clinical and genomic datasets** deposited in a public access-controlled repository in accordance to ethical obligations to the patients and to the applicable consent agreement?	Not Applicable	
Are **computational models** that are central and integral to a study available without restrictions in a machine-readable form? Were the relevant accession numbers or links provided?	Not Applicable	
If publicly available data were reused, provide the respective **data citations in the reference list**.	Not Applicable	
